# Mapping nocturnal arousal across sleep and pain disorders

**DOI:** 10.1038/s41598-026-42639-0

**Published:** 2026-03-05

**Authors:** Nazanin Biabani, Fábio Mendonça, Carlotta Mutti, Panagis Drakatos, Fernando Morgado-Dias, Antonio G. Ravelo-García, Gulcin Benbir Senel, Heidur Gretarsdottir, Peter J. Goadsby, Robert J. Thomas, Oliviero Bruni, Raffaele Ferri, Liborio Parrino, Ivana Rosenzweig

**Affiliations:** 1 Sleep and Brain Plasticity Centre, Department of Neuroimaging, Institute of Psychiatry, Psychology and Neuroscience (IoPPN), King’s College London (KCL), De Crespigny Park, Box 089, London, SE5 8AF UK; 2 Faculty of Exact Sciences and Engineering, University of Madeira, Funchal, Portugal; 3 Interactive Technologies Institute (ITI/LARSyS), ARDITI, Funchal, Portugal; 4 Sleep Disorders Centre, Department of General and Specialized Medicine, University of Parma, Parma, Italy; 5 School of Basic and Medical Biosciences, Faculty of Life Science and Medicine, King’s College London, London, UK; 6 Sleep Disorders Centre, Guy’s and St Thomas’ NHS Foundation Trust, London, UK; 7 Institute for Technological Development and Innovation in Communications, Universidad de Las Palmas de Gran Canaria, Las Palmas de Gran Canaria, Spain; 8 Department of Neurology, Cerrahpaşa Faculty of Medicine, Istanbul University-Cerrahpaşa, Istanbul, Turkey; 9 Reykjavik University Sleep Institute, School of Technology, Reykjavik University, Reykjavik, Iceland; 10 NIHR-Wellcome Trust King’s Clinical Research Facility, King’s College London, London, UK; 11 Division of Pulmonary, Critical Care & Sleep Medicine, Department of Medicine, Beth Israel Deaconess Medical Center, 330 Brookline Avenue, Boston, MA 02215 USA; 12 Department of Human Neuroscience, Sapienza University, Rome, Italy; 13 Sleep Research Centre, Oasi Research Institute-IRCCS, Troina, Italy; 14 Division of Biomedical Sciences, King Abdullah University of Science and Technology, Thuwal, Saudi Arabia

**Keywords:** Sleep disorders, IRBD, NREM parasomnia, Cyclic alternating pattern (CAP), A‑phase index (API), Longitudinal sleep microstructure, Arousal dysregulation, Diseases, Neuroscience

## Abstract

Sleep’s microstructure emerges from the continual coupling of intrinsic arousal with the homeostatic descent of the night. To ask how this coupling unfolds across disorders, we mapped the nocturnal trajectory of phasic events in idiopathic REM sleep behaviour disorder (iRBD; *n* = 19), narcolepsy type 1 (NT1; *n* = 19), NREM parasomnia (*n* = 18), fibromyalgia (*n* = 13) and healthy controls (*n* = 40). Using an automated, cyclic alternating pattern (CAP)- inspired, A‑phase index (API), a B‑phase–agnostic, 60‑s read‑out of phasic burden, we profiled subtype‑specific activity across normalised nights in retrospective polysomnography. In controls, early‑night slow‑wave–dominant phasic responses rose steeply and decayed with dissipating pressure, consistent with thalamocortical homeostasis. In iRBD and NT1, early-night A1 prominence was attenuated, and in fibromyalgia it was broadly reduced across the night. NREM parasomnias showed a distinctive pattern: transient amplification of phasic drive in early, with later attenuation in deep NREM sleep. Stage‑wise comparisons showed the largest group separations in N2 for A1/A2; between group differences were described from normalised time curves without inferential testing. These trajectories suggest that disorders may differ in how intrinsic arousal couples to homeostatic drift across the night. However, findings are hypothesis‑generating given unmatched controls, small groups and lack of adjustment for additional confounds. Descriptive prospective work combining A- and B-phase estimation is warranted.

**Supplementary Information:**

The online version contains supplementary material available at 10.1038/s41598-026-42639-0.

## Introduction

Sleep stability is an achievement, not a default^1^. In non‑rapid eye‑movement (NREM) sleep the cortex remains quaveringly responsive, punctuated by brief phasic surges of activity that mark the brain’s intrinsic arousal system at work^2^. The cyclic alternating pattern (CAP) formalises this alternation between A‑phases (transient activation) and B‑phases (relative quiescence) into a recognisable grammar of sleep microstructure. Within the A‑phases, canonical subtypes A1–A3 scale with increasing cortical and autonomic activation, with A1 closely allied to slow‑oscillatory synchronisation and A3 to near‑arousal states^3^. This view, shaped by foundational work from Terzano, Parrino and Halász, and many others, over the last fifty years, places CAP at the heart of sleep’s stability–instability balance rather than at its periphery^3–7^.

Recent guidance has refined scoring conventions and emphasised CAP’s role as a dynamic, state‑regulating phenomenon, complementary to stage‑based macro‑architecture^8^. What matters is not only how much A1, A2 or A3 occurs, but when and how these phasic events are threaded through the night’s descent and ascent in sleep depth. The 2001 atlas and subsequent updates formalised A/B alternation and subtype features^7^; our study adheres to these definitions while focusing specifically on the temporal burden of A‑phases across the night^7,9,10^.

Arousal, in this framework, is not noise but a regulator. Halász and colleagues have long argued that micro‑arousals and CAP subtypes orchestrate transitions in sleep depth and sensory gating, with A1 supporting slow‑wave consolidation and A2/A3 opening the gate toward REM or wake^2^. That arousal‑sleep interplay is thought to express homeostatic and ultradian pressures over the night, with phasic events waxing and waning as sleep pressure dissipates^11^.

A complementary, physics‑inflected working model situates these processes near critical regimes of network excitability: sleep maintains itself by flirting with instability^1^. In such a view, CAP A‑phases instantiate brief excursions along an attractor landscape, and their subtype‑specific prevalence across the night may index how close the system runs to a bifurcation. This is a working hypothesis that links microstructure to computation and metastability^1^. It should be noted that our analysis is designed to remain descriptive, hypothesis generating, rather than causal.

Clinically, the timing of A‑phases may be as informative as their totals. Most prior work aggregates CAP into nightly indices or stage‑wise means, potentially obscuring disorder‑specific trajectories across the night^3,5^. Automated approaches now make these trajectories tractable at scale, yet most pipelines target full CAP cycle reconstruction; comparatively few quantify longitudinal A1–A3 burden as a standalone arousal signature^9^.

Here we present a pilot analysis that maps the nocturnal course of A‑phase subtypes in four conditions that sample distinct axes of arousal dysregulation: idiopathic REM sleep behaviour disorder (iRBD), narcolepsy type 1 (NT1), NREM parasomnias, and fibromyalgia. iRBD offers a prodromal neurodegenerative window; NREM parasomnias index state‑boundary lability; NT1 exemplifies dysregulated sleep–wake gating; fibromyalgia syndrome anchors pain‑related sleep instability. Prior CAP work in iRBD suggests microstructural alterations with potential prognostic significance^12–15^; our aim is not to replicate those indices but to characterise the overnight patterning of A‑phase subtypes across disorders.

Crucially, the A-phase index (API) quantifies the proportion of NREM time labelled A1, A2 or A3 in non-overlapping 60-s windows^9^. API does not model B‑phases nor reconstruct A–B sequences and must not be interpreted as a CAP index; rather, it provides a time‑resolved read‑out of arousal burden within NREM. We view API as complementary to cycle‑level CAP metrics defined by the atlas and a pragmatic stepping‑stone towards joint A‑ and B‑phase estimation in future prospective work.

By focusing on when A1–A3 rise and fall across the night, instead of solely how much, this study tests whether different disorders exhibit distinct nocturnal arousal fingerprints. Such longitudinal phenotypes could sharpen biomarker work, help stratify mechanistic subtypes, and ultimately guide target selection for neuromodulatory or pharmacological interventions that aim to tune the arousal–stability balance rather than merely suppress it.

## Methods and materials

### Study design

We conducted a retrospective, exploratory, cross-sectional study of 109 adults (≥ 18 years) spanning five diagnostic categories: idiopathic REM sleep behavior disorder (iRBD; *n* = 19), narcolepsy type 1 (NT1; *n* = 19), non-REM parasomnia (NREMP; *n* = 18), fibromyalgia syndrome (FM; *n* = 13)^16^, and healthy controls (*n* = 40; from The Montreal Archive of Sleep Studies [MASS])^16^. Clinical diagnoses followed the International Classification of Sleep Disorders, Third Edition (ICSD-3), and were confirmed by board‑certified sleep physicians^17,18^. Inclusion required age ≥ 18 years; exclusion criteria comprised major neurological or psychiatric comorbidity, substance dependence, and medications known to alter sleep architecture.

### Ethical approval

for the study was granted by the institutional GSTT Research Ethics Committee (Project No. 12436). The analysis was conducted on fully anonymized retrospective data, in compliance with the UK Data Protection Act and the General Data Protection Regulation (GDPR) (Regulation (EU) 2016/679)^19,20^. Informed consent was not required due to the retrospective design and the use of non-identifiable data^19,20^. The study was carried out in accordance with the Declaration of Helsinki (WMA, 2013).

All overnight polysomnographic (PSG) recordings included standard six-channel EEG using a 10–20 montage (F3, F4, C3, C4, O1, O2). Sleep staging was performed manually in accordance with American Academy of Sleep Medicine (AASM) criteria^10^. Recordings were sampled at 512 Hz and band-pass filtered according to local clinical protocols (typically 0.3–35 Hz for EEG). Sleep stages were scored in 30-s epochs following the AASM manual (version 3)^10^. Referencing schemes were harmonised during preprocessing by re-referencing all EEG channels to the linked mastoids, ensuring a consistent montage across MASS and clinical studies. Definitions follow the CAP atlas and later clarifications; we quantify A‑phases only and do not reconstruct A–B sequences^9^. All patients underwent routine clinical evaluation including PSG-derived apnoea–hypopnoea index (AHI) and periodic limb movement indices. Dedicated pre-study screening nights were not performed. Individuals with moderate-to-severe sleep-disordered breathing or periodic limb movement disorder were not included; residual mild respiratory or limb movement findings (Table 1) were allowed and summarised descriptively but not used as covariates. Summary socio demographic and sleep architecture metrics are provided in Table 1.

**Table 1:** Cohort characteristics and sleep macro‑architecture. Values are mean ± SD; Female, % is the proportion of women in each group.

Metric	Control	iRBD	NT1	NREM parasomnia	Fibromyalgia
n	40	19	19	18	13
Female, %	50.0	10.5	52.6	55.6	92.3
Age (years)	46.2 ± 16.4	60.5 ± 9.7	29.9 ± 10.9	39.6 ± 6.9	44.5 ± 6.1
TST (mins)	420.7 ± 35.6	364.2 ± 55.7	413.4 ± 45.3	385.4 ± 42.8	348.6 ± 84.6
SE (%)	84.4 ± 8.2	76.4 ± 11.9	86.1 ± 5.7	86.1 ± 8.0	72.9 ± 15.6
WASO (mins)	59.3 ± 39.1	91.4 ± 47.6	60.1 ± 27.2	54.4 ± 41.7	101.7 ± 63.7
Arousal Index (ev/hr)	11.6 ± 4.0	20.5 ± 8.0	16.8 ± 5.1	15.4 ± 6.4	20.7 ± 9.8
AHI (Ev/Hr)	1.9 ± 0.7	3.5 ± 3.0	1.4 ± 1.3	1.2 ± 1.8	1.4 ± 1.6
N1% of TST	8.8 ± 4.1	12.0 ± 5.5	10.2 ± 5.7	8.7 ± 2.8	14.0 ± 12.5
N2% of TST	47.2 ± 7.0	42.4 ± 8.8	43.1 ± 7.1	48.1 ± 8.4	40.0 ± 7.9
N3% of TST	21.2 ± 6.0	24.8 ± 6.7	26.1 ± 6.6	21.6 ± 8.2	24.9 ± 7.5
REM % of TST	22.7 ± 5.0	20.8 ± 6.4	20.6 ± 5.2	21.6 ± 4.6	21.0 ± 6.8

TST, total sleep time; SE, sleep efficiency; WASO, wake after sleep onset; AHI, apnoea–hypopnoea index. Controls were drawn from MASS; patient cohorts were recruited at a tertiary sleep centre. See Supplementary Methods for inclusion/exclusion criteria and scoring rules.

### Automatic detection of CAP A phases and definition of the A phase index (API)

**Fig. 1:**
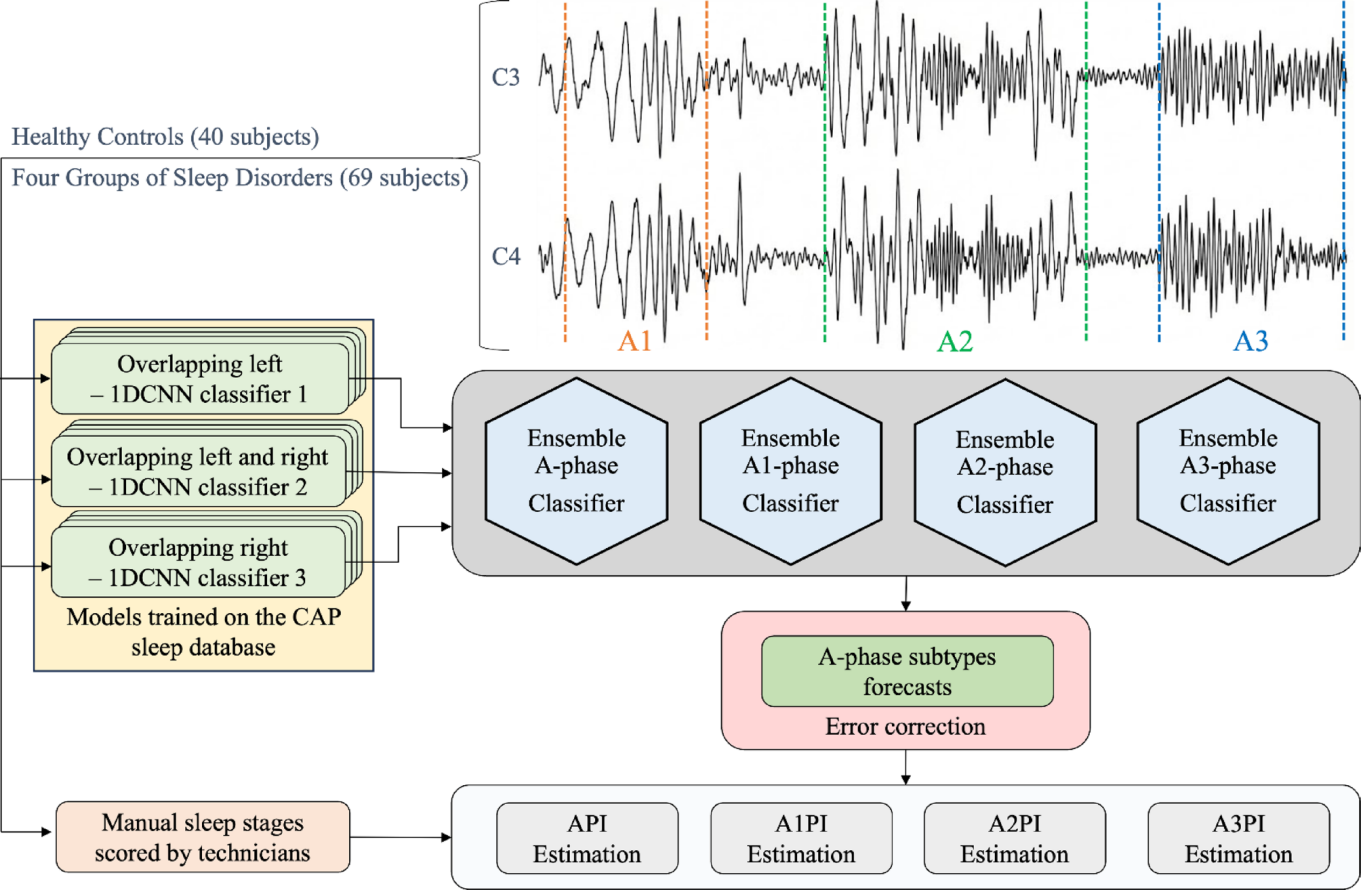
Automated CAP A-phase detection and A-phase index (API) pipeline^9,21^. Overnight EEG and accompanying PSG signals are recorded using standard clinical montages. (**a**) Central EEG derivations (C3 and C4) are fed to the validated automated CAP A-phase detector of Mendonça et al. (2023), which assigns per-sample labels A1, A2 or A3^2^. The outputs from the subtype models were combined through an ensemble forecast. This forecast, at each second, determined the class with the highest probabilistic output from the three subtype models. To align with the CAP scoring rules, a correction procedure was applied. It involved discarding estimations lower than 2 s and converting annotations to the most prevalent subtype during sequences of alternating subtypes. For illustration, schematic waveforms on the top show stylised examples of A1 (slow, high-amplitude), A2 (mixed), and A3 (faster, lower-amplitude) events; these are idealised motifs based on CAP atlas descriptions rather than individual patient traces. (**b**) Detector outputs are restricted to NREM sleep and aggregated within non-overlapping 60-s windows. For each window and subtype k (A1, A2, A3), the A-phase index API_k_ is defined as time-in-A_k_ divided by time-in-NREM within that window, yielding a stage-aware, channel-averaged measure of A-phase burden across the night. (**c**) Windowed API values are then analysed at the level of sleep stages (N1, N2, N3) and as trajectories over normalised time-of-night, and summarised as early–late differences (ΔAPI). The API does not model B-phases or full A–B CAP cycles and is therefore not a CAP index; it provides a time-resolved read-out of A-phase subtype burden within NREM sleep. CAP, cyclic alternating pattern; API, A‑phase index; AASM, American Academy of Sleep Medicine; NREM, non‑rapid eye movement; C3/C4, central EEG derivations.

We estimated an A‑phase index (API; A1/A2/A3), defined as the proportion of NREM sleep time labelled as A‑phase within non‑overlapping 60‑s windows; B‑phases were not detected, and CAP index (sequences·h⁻¹) was not computed (see Fig. 1)^9,21^. Throughout, we use ‘burden’ as a neutral term for the proportion of NREM time spent in A-phases (API), without implying pathological load. CAP A-phases (A1, A2, A3) were detected using the validated automated classifier of Mendonça et al. (2023) without modification. That model was trained and cross-validated on expert-scored CAP according to the European Sleep Research Society (ESRS) consensus rules, and we used its per-sample A1/A2/A3 labels as inputs to all subsequent API computations^21^. The model’s outputs were filtered based on manual sleep scoring to eliminate A-phase annotations during epochs classified as non-NREM sleep^21^. Automated detection of A phases from the cyclic alternating pattern (CAP) was performed using electroencephalographic (EEG) signals collected from channel C3 (representative of the left hemisphere) and channel C4 (representative of the right hemisphere)^21^. In addition, potential interhemispheric differences were also assessed. The analysis focused on three principal CAP subtypes: A1, A2, and A3 (Fig. 1), as previously published, see Fig. 1 for schematic^9,21^.

API for A1, A2 and A3 was calculated separately in 60-s windows across each subject’s entire night and within each NREM sleep stage^21^. Subsequently, group averages were computed and visualised with the x‑axis as a normalised night coordinate [0–1] (lights‑out to lights‑on) and the y‑axis as API^21^. Hemispheric differences (C3 vs. C4) were assessed; unless specified, group values average C3/C4.

For each channel (C3, C4) and non‑overlapping 60‑s window w, the classifier outputs sample‑level labels A1, A2 or A3^9,22^. For subtype k ∈ {A1, A2, A3}, the window‑level A‑phase index is:

$${API}_{k}\left(w\right)=\frac{{T}_{A\_k,w}}{{T}_{NREM,w}}$$

where T_{A_k, w} is the cumulative duration (s) of A‑phase k within w and T_{NREM, w} is the NREM duration (s) within w^9,22^.

**Fig. 2:**
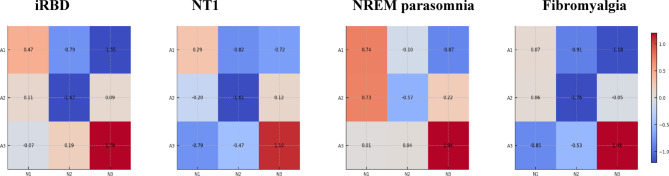
Control‑normalised, stage‑stratified deviation in A‑phase burden (A1–A3). Each panel displays a heat map for a clinical group (iRBD, NT1, NREM parasomnia, Fibromyalgia) showing the deviation of the A‑phase index (API) from controls for each NREM stage (columns: N1, N2, N3) and A‑phase subtype (rows: A1, A2, A3). Values are Glass’s Δ effect sizes computed per cell as the group mean minus the control mean, divided by the control standard deviation, i.e., Δ = (µ_group − µ_control)/σ_control. The colour map is zero‑centred and diverging (cooler tones = lower than control; warmer tones = higher than control; near‑white ≈ no difference). *What the figure shows*. Relative to controls, iRBD and NT1 exhibit lower A1/A2 in N2 (negative Δ), with iRBD also showing lower A1 in N3. NREM parasomnia shows higher A1/A2 in N1 together with lower A1 in N3, and fibromyalgia shows lower A1/A2 in N2 and lower A1 in N3. Because Δ is scaled by the control variability per cell, magnitudes reflect both mean shifts and the stability of the control distribution. *Scope and caveats*. API quantifies A‑phase burden only; CAP B‑phases and CAP A–B cyclicity were not modelled, so Δ values should not be read as CAP index. This figure averages hemispheres; channel‑specific statistics and hypothesis tests are provided in the main text and Supplement. API, A‑phase index; CAP, cyclic alternating pattern; NREM, non‑rapid eye movement; iRBD, idiopathic REM sleep behaviour disorder; NT1, narcolepsy type 1; Δ (Glass’s Δ), standardised difference using the control standard deviation; C3/C4, central EEG derivations.

For stage‑wise summaries and all inferential tests, windows with T_{NREM, w} = 0 were omitted (Fig. 2). For the longitudinal trajectories and ΔAPI/ΔΔAPI summaries, windows outside NREM sleep were retained with API_k(w) = 0 (i.e., zero‑filled) to preserve the lights‑out to lights‑on timeline^21^.

We define the early–late night contrasts as:

$$\Delta API={mean\left(API\right)}_{T1}-{mean\left(API\right)}_{T3}$$

$$\Delta \Delta API={\Delta API}_{group}-{\Delta API}_{control}$$

API reflects A‑phase burden and should not be interpreted as CAP index; B‑phases and A–B sequences were not modelled^9,21^. Because A1 activity often has a frontal maximum and our analysis uses only central derivations (C3/C4), topographic generalisation beyond these sites should be made cautiously.

### Normalised time‑of‑night and group‑level trajectories

**Fig. 3:**
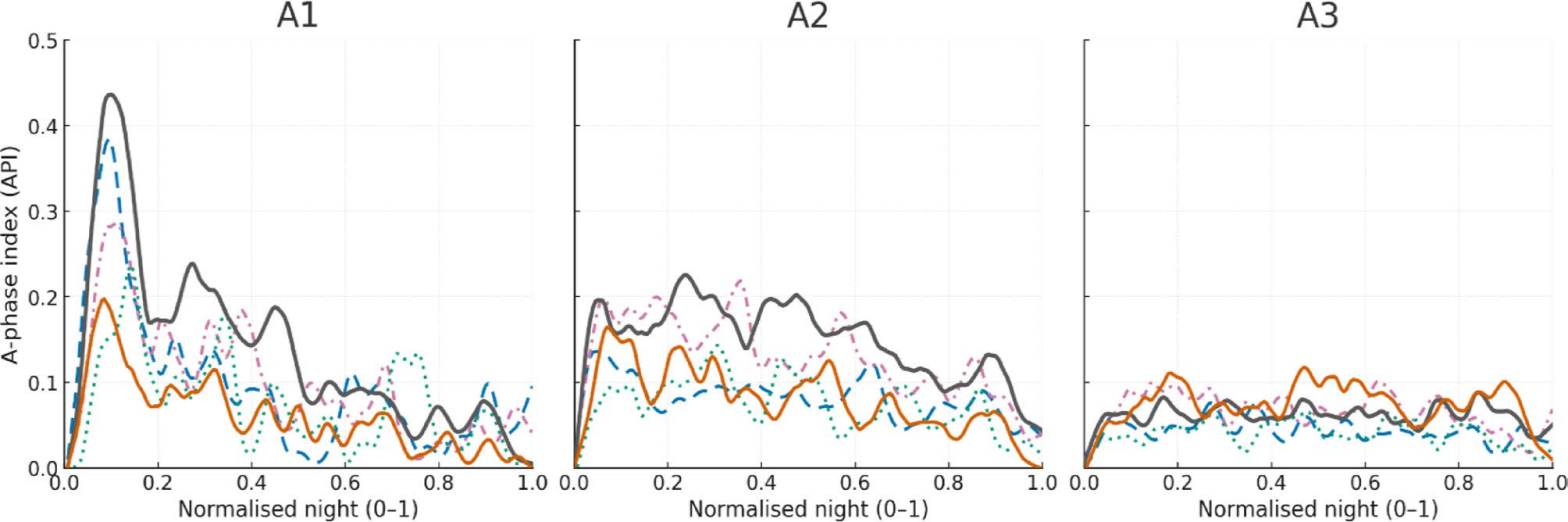
Nocturnal trajectories of CAP A‑phase burden by subtype. Group mean A phase index (API) trajectories across the normalised night (0–1; lights out to lights on). Curves are lightly smoothed for display (LOESS span = 0.10) with 95% bootstrap confidence ribbons and are interpreted descriptively. Colours/line styles: Control (grey, solid, thicker), idiopathic REM sleep behaviour disorder: iRBD (orange, solid), narcolepsy type 1: NT1 (blue, dashed), NREM parasomnia (magenta, dash dot), fibromyalgia syndrome: FMS (teal, dotted). Across groups, A1 shows early night prominence with progressive decline, A2 is flatter, and A3 remains low amplitude. Relative to controls, iRBD and NT1 exhibit depressed A1/A2 burden over much of the night; NREM parasomnia shows early night A1/A2 excess; fibromyalgia presents broadly reduced A phase burden, consistent with disorder specific microstructural instability. Interpretation note: API reflects A phase burden and must not be read as CAP index (no B phase or A–B cyclicity was modelled). Stage wise and hemispheric analyses are given in the companion figures and Supplementary Tables. Curves are qualitative visual summaries; no time×group inference was performed. API, A phase index; CAP, cyclic alternating pattern; NREM/REM, non/rapid eye movement sleep; iRBD, idiopathic REM sleep behaviour disorder; NT1, narcolepsy type 1; FMS, fibromyalgia syndrome; C3/C4, central EEG derivations.

For longitudinal analyses, we aligned each subject’s windows to a unit interval [0,1] from lights‑out to lights‑on, thereby allowing night‑to‑night duration differences to be collapsed onto a common time axis. The subject‑level trajectory for subtype k is the sequence of window‑wise API_k across the night. For group visualisations we averaged these trajectories at each time point and displayed curves with pointwise 95% confidence ribbons (see Fig. 3). Channel‑wise API values were averaged (C3/C4) unless hemispheric asymmetry was assessed explicitly.

**Fig. 4:**
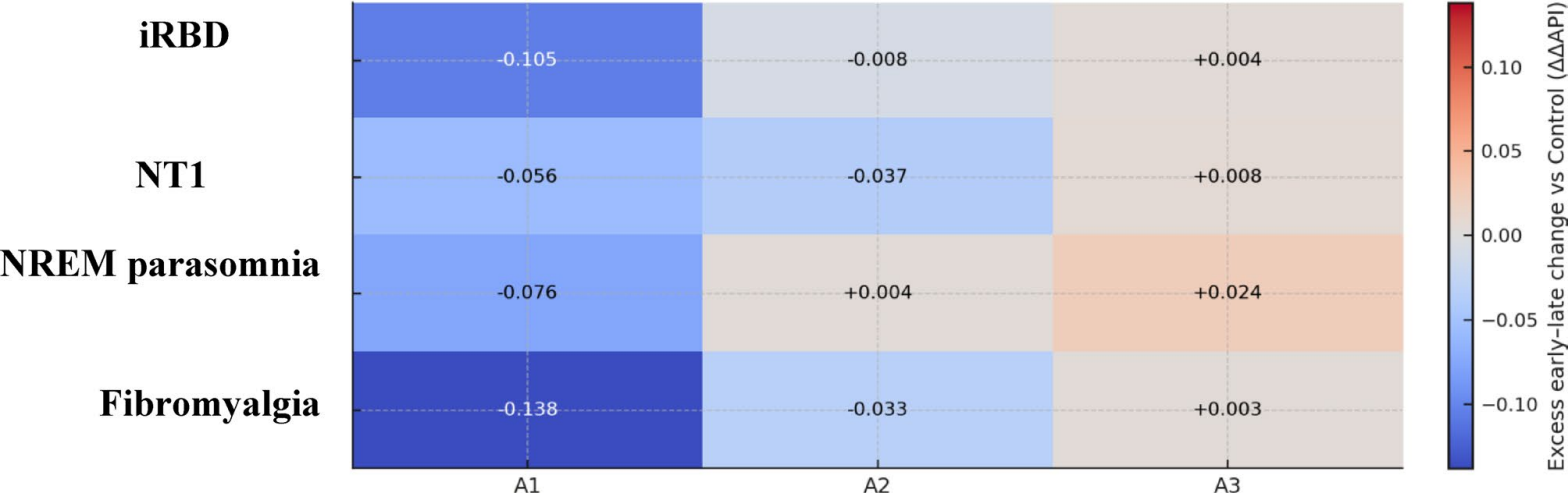
Control‑normalised early–late change in A‑phase burden (ΔΔAPI) across disorders. Heat map summarising group‑wise early–late nocturnal change in API relative to controls. ΔAPI is early vs. late night within each subject; ΔΔAPI is the group–control difference in that change. For each subject, ΔAPI is the mean difference between the first and last quartiles of the normalised night (Q1: t_norm ≤ 0.25; Q4: t_norm ≥ 0.75): ΔAPI = mean(API)_Q1 − mean(API)_Q4. Group values are averaged across subjects and hemispheres; ΔΔAPI is the group–control difference for the same subtype. Units are in API proportion (dimensionless). A diverging blue–white–red colour map is centred at zero: cool (blue) tones denote negative ΔΔAPI (flatter overnight decline than controls), warm (red) tones denote positive ΔΔAPI (steeper decline), and near‑white indicates values close to zero. *Interpretation*. Consistent with the trajectories (Fig. 3), A1 shows the largest group–control separations with the greatest attenuation in iRBD and FMS; NT1 is reduced mainly for A2, and NREM parasomnia shows a modestly steeper A3 decline. ΔΔAPI summaries are descriptive and complement stage‑wise statistics. API, A‑phase index; ΔAPI, early–late change in API; ΔΔAPI, group–control difference in ΔAPI; Q1/Q4, first/last quartile of the normalised night; CAP, cyclic alternating pattern; NREM, non‑rapid eye movement; iRBD, idiopathic REM sleep behaviour disorder; NT1, narcolepsy type 1; FMS, fibromyalgia syndrome; C3/C4, central EEG derivations.

For each subject and subtype (A1–A3), we summarised the nocturnal change in NREM A-phase burden as an early–late difference. Early and late night were defined as the first and last quartiles of the normalised night (t_norm ≤ 0.25 and t_norm ≥ 0.75). At each minute, API values were first averaged across EEG channels (C3, C4). ΔAPIₖ was then defined as the difference between early-night and late-night API for subtype k: ΔAPIₖ = mean_early(APIₖ) − mean_late(APIₖ). We defined ΔΔAPIₖ as the group–control difference in ΔAPIₖ for the same subtype (Fig. 4).

Unsmoothed group‑mean trajectories underlying Fig. 3 are provided as *Supplementary Data*. Trajectories were computed from unsmoothed 60-s window API vectors; per-subject curves were linearly interpolated to a common 0–1 grid (400 points) before group averaging; no LOESS or other smoothing was applied to the source data.

### Statistical analysis

Group contrasts are based on non-parametric tests that respect the study’s cross-sectional design. For each subtype and stage we compared patient groups with controls using two‑sided Mann–Whitney U tests. Adjusted p-values use Bonferroni correction across A1/A2/A3 within each stage family (Stage N1 (N1), Stage N2 (N2), Stage N3 (N3)), and separately for aggregated NREM sleep. For primary group contrasts (patient vs. control within each stage), we report the larger of the two adjusted p-values across hemispheres (C3 vs. C4). Where lateralised patterns were plausible or observed, we also report channel-specific results explicitly labelled by channel.

Longitudinal differences in trajectory shapes and ΔΔAPI were summarised descriptively without inferential testing. To provide a simple inferential summary of longitudinal group differences, we compared ΔAPI across diagnostic groups using Kruskal–Wallis tests for each subtype (A1–A3). Where appropriate, we explored pairwise group–control differences in ΔAPI using Mann–Whitney U tests with rank-biserial effect sizes. Given three global tests (one per subtype), we used Bonferroni correction (α${}_{\mathrm{corrected}}$ = 0.0167) for the Kruskal–Wallis p-values. Pairwise tests were considered exploratory and are reported uncorrected in the Supplement.

### Sensitivity models (age/sex adjustment and hemispheric contrasts)

**Fig. 5:**
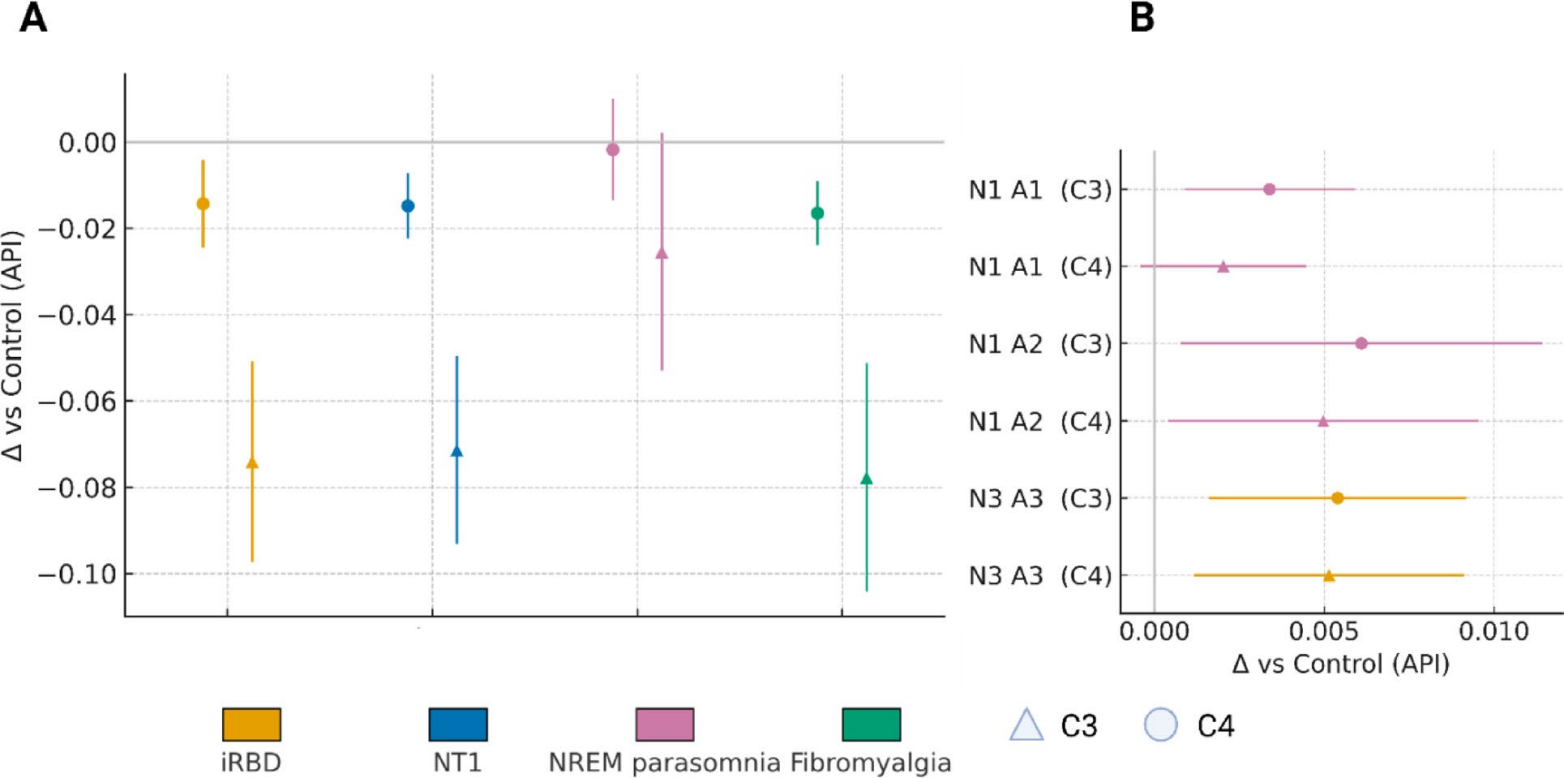
Sensitivity snapshot of CAP A‑phase burden contrasts. (**a**) and (**b**) summarise group–control differences in the A‑phase index (API) derived from automated CAP‑A detection in 60‑s windows restricted to NREM sleep (C3, C4; Methods). API quantifies A‑phase burden as time‑in‑A1/A2/A3 per NREM window and is not a CAP index; B‑phases and A–B cyclicity were not modelled. In (**A**), stage‑specific effects in N2 for subtypes A1 and A2 are depicted. Points denote the mean difference from controls (Δ vs. Control; API units) for each disorder; error bars indicate 95% confidence intervals. Marker shape encodes channel (circle = C3; triangle = C4). Colour encodes diagnostic group: iRBD (orange), NT1 (blue), NREM parasomnia (magenta), Fibromyalgia (teal). The horizontal grey line marks zero (no difference). Negative values indicate lower A‑phase burden than controls; positive values indicate higher burden. Across cohorts, the largest separations occur in N2 A2 (iRBD, NT1 < Control) and N2 A1 (iRBD, Fibromyalgia < Control); NREM parasomnia shows small or mixed effects. In (**B**) (illustrative, no inference), channel‑specific highlights illustrating our reporting convention for hemispheric analyses are shown. The panel shows contrasts selected a priori from the main analysis: NREM parasomnia in light sleep (N1) with higher A1/A2 relative to controls, most apparent in C3, and iRBD in deep sleep (N3) with higher A3 in C3. Each estimate is expressed as Δ vs. Control (API); shapes and colours as in (a). These examples demonstrate how channel‑wise reporting is handled when hemisphere‑specific effects are plausible. *Scope and caveats*. Estimates are descriptive summaries of stage‑wise group means versus controls; inferential statistics for all stage/subtype/channel contrasts are reported in Supplementary Tables. Because API reflects only A‑phase burden, results should not be interpreted as CAP index or as evidence about CAP cyclicity that includes A–B sequencing.

To complement the prespecified Mann–Whitney group–control comparisons (Bonferroni adjusted within stage families) and to illustrate hemispheric examples, we fitted compact covariate adjusted linear models at the subject level (Fig. 5). For each CAP subtype and stage, the outcome was the subject’s stage specific API (C3/C4 averaged unless channel effects were examined), and the predictors were diagnostic group (controls as reference), age (years; mean centred) and sex (female vs. male). Models used ordinary least squares with Huber–White robust standard errors; coefficients are interpreted as Δ vs. control in API proportion, with 95% compatibility intervals shown in Fig. 5a. Because API values were modest and residual diagnostics were acceptable, linear models provided estimates concordant with the non parametric results; inference for the manuscript remains anchored to the latter.

For Fig. 5b we fitted channel wise models including factors channel (C3 vs. C4) and a group×channel interaction, and we extracted contrasts for two a priori exemplars motivated by the stage wise findings: NREM parasomnia: N1 A1/A2; iRBD: N3 A3. These are presented descriptively (point estimate ± 95% CI) to illustrate our reporting convention for hemispheric analyses; multiplicity adjustment was not applied because the panel is expository. All other analysis choices follow those described above (windowing, staging, API definition, and primary tests).

#### Choice of effect size metrics

To avoid mixing metrics, we prespecified one effect size per comparison type. For two-sample Mann–Whitney tests, the primary effect size is the rank-biserial correlation (*r* < sub> rb), computed from the U statistic as *r* < sub> rb</sub > = 2U/(n < sub> group·n < sub> control) − 1 (controls coded as the reference). We report *r* < sub> rb alongside U and Bonferroni-adjusted two-sided *p* values. For control-referenced visual summaries (e.g., the stage-stratified heat maps), we use Glass’s Δ = (µ < sub> group − µ < sub> control)/σ < sub> control. For the covariate-adjusted linear models (Fig. 5), we present unstandardised differences in API (Δ vs. control) with robust 95% CIs and do not introduce an additional effect-size metric. We use Glass’s Δ here as a visual normalisation that scales differences by control variability; it is not intended as a formal meta-analytic effect size.

### Visualisation standards

*Window length*: We chose non-overlapping 60-s windows because CAP A-phases are sparse and tend to occur in bursts; 60-s aggregation provides a stable estimate of A-phase burden within each epoch while preserving the coarse temporal evolution across the night. This window length was therefore chosen to prioritise stability over sub-minute temporal precision.

*Trajectories*: Per-subject trajectories were constructed by mapping window-level API_k values (k = A1, A2, A3) onto the normalised time-of-night axis (t_norm ∈ [0,1]) and, where relevant, stratifying by NREM sleep stage. Group summaries were obtained as the mean trajectory across subjects with 95% confidence bands. Where smoothing is shown, we applied LOESS with span 0.10 to the group-mean trajectories for display only; all statistical analyses were performed on unsmoothed API values.

*Stage-wise contrasts and heatmaps*: Stage-stratified group–control differences in API_k were visualised as heatmaps of Glass’s Δ, defined as (µ_group − µ_control)/σ_control, for each combination of subtype (A1–A3) and NREM sleep stage (N1, N2, N3). These maps are intended as descriptive summaries of effect size patterns; formal inference relies on the Mann–Whitney tests and linear models described in Sect. 2.4.

*Classifier validation*: Event-level performance metrics (precision, recall, F1) for the automated CAP A-phase detector, including validation on NREM sleep subsets, are reported in detail in Mendonça et al. (2023)^21^ and are thus not repeated here.

## Results

We analysed adults across five diagnostic groups: iRBD, NT1, NREM parasomnias, fibromyalgia syndrome (FMS) and healthy controls. Cohort characteristics and macro‑architecture are summarised in Table 1. Controls were not matched on age/sex to each clinical group (Table 1); between‑group inferences are interpreted cautiously. Controls were younger on average than some clinical groups; sleep efficiency and wake after sleep onset showed the expected degradation in iRBD, whereas stage proportions were broadly comparable otherwise (Table 1). In this tertiary-care sample, N3% in the fibromyalgia group was comparable to controls (Table 1), consistent with reports that macro-architectural slow-wave proportions can be preserved even when sleep is subjectively non-restorative.

Overnight trajectories of A‑phase burden, expressed as the A‑phase index (API), revealed a reproducible dissipation of A1 from early to late night in all groups (Fig. 3). The early‑night A1 prominence was blunted in each clinical cohort relative to controls, most strikingly in iRBD and NT1. A2 trajectories were flatter and of lower amplitude than A1 but again showed an early‑night excess in controls that was attenuated in iRBD, NT1 and FMS. A3 remained low throughout the night with modest late‑night increases in some groups; between‑group separations were small. A simple early–late summary (ΔAPI; Fig. 4) confirmed these qualitative impressions. Controls showed the largest A1 dissipation (mean ΔAPI${}_{A1}$≈0.20), whereas all clinical cohorts exhibited reduced early–late A1 decline (fibromyalgia ≈ 0.07, RBD ≈ 0.09, NT1 ≈ 0.16, NREM parasomnia ≈ 0.13). In contrast, ΔAPI for A2 and A3 did not differ significantly across groups (*p* = 0.071 and *p* = 0.305, respectively), consistent with a primary longitudinal alteration in the slow-wave–linked A1 component.

Stage‑stratified heatmaps (Fig. 2) corroborated these patterns. Relative to controls, iRBD showed a marked reduction of A1 and A2 in N2 and a selective reduction of A1 in N3; NT1 exhibited reductions of A1 and A2 in N2 and a decrease of A3 in N1; NREM parasomnias displayed an increase of A1/A2 in N1 with a concomitant reduction of A1 in N3; and FMS demonstrated reductions of A1/A2 in N2 and a reduction of A1 in N3. Full statistics are provided below and in Supplementary Tables.

*Sensitivity snapshot (*Fig. 5*).* Seen through the CAP lens, Fig. 5a localises the main group separations to the N2 regime where A1 and A2 ordinarily scaffold continuity, and Fig. 5b demonstrates, by example, how channel-wise estimates can be displayed without over-interpreting hemispheric differences. Age- and sex-adjusted estimates for the stage N2 contrasts (Fig. 5) reproduced the main pattern without sign reversals: A1 and A2 were lower than controls in iRBD, NT1 and FMS, whereas estimates for NREM parasomnia lay closer to zero. Magnitudes and uncertainty closely paralleled the stage wise non parametric results, indicating that the principal differences were not explained by the age/sex imbalance between cohorts (notably older iRBD and a higher proportion of women in FMS). These adjustments are therefore supportive rather than probative; formal inference remains with the primary tests.

*Hemispheric highlights* (Fig. 5b). Channel specific examples, presented for exposition, aligned with the channel averaged findings. In NREM parasomnia, C3 (left central) showed higher N1 A1/A2 than controls with wider intervals at C4, echoing the stage wise N1 increases (especially for A1 at C3). In iRBD, C3 showed higher N3 A3 than controls, consistent with the observed N3 A3 increase in C3 and near threshold signals elsewhere. Paired C3–C4 tests across the full analysis set did not reveal systematic hemispheric asymmetry; these panel b contrasts are therefore illustrative of this reporting convention rather than a claim of hemispheric pathophysiology.

*Idiopathic REM sleep behaviour disorder*. Across NREM, iRBD showed lower overall A1 (U = 654, z = 4.445, η² = 0.335, adjusted *p* = 2.74 × 10⁻⁵) and A2 (U = 621, z = 3.910, η² = 0.259, adjusted *p* = 2.87 × 10⁻⁴) than controls; A3 did not differ (adjusted *p* ≈ 1.0). Stage‑wise, A1 and A2 were reduced in N2 (A1: U = 596, z = 3.504, adjusted *p* = 0.002; A2: U = 672, z = 4.737, adjusted *p* = 6.78 × 10⁻⁶) and A1 was reduced in N3 (U = 653, z = 4.429, adjusted *p* = 2.95 × 10⁻⁵). A3 in N3 showed a non significant trend after hemisphere aggregation (max adjusted *p* = 0.052); a C3 only comparison yielded adjusted *p* = 0.011 (Fig. 2; Table 2; *Supplementary Tables 14–16)*.

**Table 2:** Whole‑night non‑REM A‑phase index (API) by subtype and group, and adjusted p‑values for group–control contrasts. Means reflect API averaged over C3/C4 and over all NREM epochs; p‑values are Bonferroni‑adjusted values taken from the validated pairwise tests in the Supplement (conservative maximum of C3/C4).

Subtype	Control	iRBD	NT1	NREM Parasomnia	Fibromyalgia	adj *p* (iRBD vs. Ctrl)	adj *p* (NT1 vs. Ctrl)	adj *p* (NREMP vs. Ctrl)	adj *p* (FMS vs. Ctrl)
A1	0.139	0.061	0.098	0.099	0.075	2.74e-05	0.044	0.045	0.002
A2	0.149	0.082	0.085	0.129	0.075	0.000287	0.001	0.923	0.001
A3	0.063	0.074	0.045	0.069	0.045	1	0.183	1	0.489

API, A‑phase index; NT1, narcolepsy type 1; NREMP, non‑REM parasomnia; FMS, fibromyalgia syndrome. Note that API quantifies A‑phase burden and should not be interpreted as CAP index.

*Narcolepsy type 1*. Overall NREM A‑phase burden was decreased for A1 (0.098 vs. 0.138 in controls; U = 531, z = 2.450, adjusted *p* = 0.044) and A2 (0.080 vs. 0.151; U = 637, z = 4.169, max adjusted *p* = 0.001); A3 showed no difference. By stage, N2 reductions were evident for A1 (0.013 vs. 0.028; U = 594, z = 3.472, adjusted *p* = 0.002) and A2 (0.053 vs. 0.129; U = 684, z = 4.932, max adjusted *p* = 5.33 × 10⁻⁶). In N1, A3 was decreased (0.012 vs. 0.028; U = 603, z = 3.618, max adjusted *p* = 0.010), whereas N3 trends did not meet multiplicity‑corrected thresholds *(*Fig. 2; Table 2; *Supplementary Tables 6–8)*.

*NREM parasomnias*. Overall NREM A1 was lower than in controls (U = 480, z = 2.442, adjusted *p* < 0.05), with no differences in A2 or A3. Stage‑wise, C3 showed an increase of A1 and A2 in N1 (A1: U = 141, z = − 3.471, adjusted *p* = 0.001; A2: U = 180, z = − 2.791, adjusted *p* = 0.016), whereas C4 did not meet multiplicity corrected thresholds (A1 adjusted *p* = 0.167; A2 adjusted *p* = 0.073). A1 was reduced in N3 for C4 (adjusted *p* = 0.003) and showed a weaker effect in C3 (adjusted *p* = 0.068) *(*Fig. 2; Table 2; *Supplementary Tables 10–12)*.

*Fibromyalgia syndrome*. FMS showed widespread attenuation of A‑phases: overall NREM A1 (0.076 vs. 0.138; U = 437, z = 3.659, adjusted *p* = 0.001) and A2 (0.073 vs. 0.151; U = 452, z = 3.969, adjusted *p* = 2.26 × 10⁻⁴) were reduced relative to controls, with no difference in A3. By stage, N2 A1 and A2 were both reduced (A1: 0.014 vs. 0.028, adjusted *p* = 0.001; A2: 0.055 vs. 0.129, max adjusted *p* = 7.77 × 10⁻5)), and A1 was lower in N3 (adjusted *p* = 0.008). In N1, A3 was lower in C3 (0.012 vs. 0.027, max adjusted *p* = 0.008)(Fig. 2; Table 2; Supplementary Tables 2–4).

*Hemispheric comparisons and validation*. Paired C3–C4 analyses did not reveal systematic hemispheric asymmetry in API across groups; a single near‑threshold trend was observed for A3 in parasomnias (C3 < C4, unadjusted *p* ≈ 0.05). In small manual subsets (parasomnia, iRBD), NREM‑level manual vs. automated API did not show a consistent direction of difference (see *Supplementary Table S18*,* S20*). Event‑level detection performance is not reported here. Comprehensive statistics for all stage‑wise contrasts and hemispheric tests are provided in the Supplementary Tables; for channel-specific details see *Supplementary Figs. S1–S8*, and for subtype stage profiles *Supplementary Figs. S9–S14.*

## Discussion

This exploratory analysis describes how intrinsic arousal, indexed by CAP A‑phase subtypes, unfolds across the night in four clinical cohorts^2,7,9^. By concentrating on the A‑phase index (API) rather than the full CAP sequence, we isolate the burden of phasic events that sculpt NREM stability and its interaction with sleep pressure, without conflating inferences with unobserved B‑phases^9^. Two broad themes emerge. First, iRBD and narcolepsy type 1 (NT1) show attenuated A1/A2 burden in stage N2 (with A1 also reduced in N3 for iRBD), consistent with impaired slow‑wave recruitment and intermediate arousal control. Second, NREM parasomnia and fibromyalgia exhibit dissociable alterations: parasomnia shows early‑night increases in A1/A2 within N1, followed by reduced A1 in N3, whereas fibromyalgia presents a widespread attenuation of A1/A2, particularly in N2, with A1 lowered in N3. These patterns recapitulate the stage‑stratified contrasts and the overnight trajectories in Figs. 2 and 3.

The covariate adjusted estimates confirm that the N2 attenuation of A1/A2 in iRBD and NT1, and the broader A1/A2 reduction in fibromyalgia, persist after accounting for age and sex, strengthening the reading that these differences reflect microstructural regulation rather than demographic composition. Neurophysiologically, reduced A1 (the slow wave linked, anti arousal subtype) and A2 (intermediate activation) in N2 possibly indicate weaker thalamo cortical recruitment of stabilising transients under typical homeostatic pressure, whereas the illustrative channel contrasts (Fig. 5b) suggest how such effects can be sign posted without implying robust hemispheric specialisation. In parasomnia, early night N1 A1/A2 increases are arguably compatible with state boundary lability on the descent into sleep; in iRBD, higher N3 A3 may index altered near arousal transients at depth. We emphasise that primary inference remains with stage-wise non-parametric tests; Fig. 5 serves as an adjusted context and a transparent statement of our reporting convention for hemispheric comparisons. All interpretations are descriptive and compatible with multiple mechanisms; no causal claims are made.

From a systems perspective, CAP expresses a structured interplay between sleep‑promoting and arousal systems^1,3^. Subtype A1 represents a ‘slow‑wave’ anti‑arousal response that maintains continuity under high sleep pressure, whereas A2 and A3 describe progressively stronger cortical–autonomic activations^22^. The classical microstructure literature places these dynamics within a hierarchical arousal continuum and shows that A‑ and B‑phases organise NREM into a flexible, micro‑cyclic architecture^5^. Our findings, including reduced A1/A2 in iRBD and NT1, early‑night A1/A2 excess in parasomnia, attenuated A1/A2 in fibromyalgia, are all compatible with selective disturbances in thalamo‑cortical gating and the homeostatic downscaling of synapses across the night^22^.

The API trajectories also align with a physics‑influenced view of sleep regulation as a near‑critical system^1^. A1 dominance early in the night and its exponential decay thereafter reflect the dissipation of homeostatic pressure; departures from this profile may signal shifts in proximity to bifurcation points separating stable slow‑wave regimes from arousal‑prone regimes. In iRBD, reduced A1/A2 in N2 and A1 in N3 are consistent with early thalamo‑cortical dysregulation reported in prodromal α‑synucleinopathies and with clinical evidence of NREM instability^12,13^. Across the night, iRBD showed attenuation of A1/A2, particularly in N2, and a sustained reduction of A1 in N3. Clinical CAP studies report mixed macro‑patterns in iRBD, some cohorts show higher CAP rate with reduced A1 and increased A2/A3, others a reduction of overall CAP rate dominated by A1 loss, but all appear to point to fewer slow‑wave‑rich A‑events^12–15^. Our API findings isolate this A‑phase burden without assumptions about B‑phase sequencing, and therefore align with the notion that iRBD is characterised by weakened homeostatic ‘anti‑arousal’ buffering with relative preservation of higher‑arousal events.

In NT1, orexin deficiency plausibly disrupts the hierarchical organisation of NREM microstructure^23,24^, reducing slow‑wave‑linked A1 and intermediate A2 events. In our study, in keeping, narcolepsy exhibited reduced A1/A2 in N2 and reduced A3 in N1. Prior work indicates a shift towards faster transient activity and reduced slow transients during non‑REM sleep in narcolepsy with RBD features, consistent with orexin loss and unstable arousal gating^23,24^. The blunted early‑night A1 in our trajectories suggests impaired recruitment of slow‑wave‑dominated responses during periods of high sleep pressure, with consequences for consolidation of deep sleep.

In NREM parasomnia, the combination of elevated A1/A2 in light sleep and reduced A1 in deep sleep fits state‑dissociation models in which partial arousals arise from an unstable coexistence of wake‑like and sleep‑like activity^25^. We observed an overall reduction in A1 across non‑REM, alongside early‑night elevations of A1/A2 within N1. This pattern is compatible with a state of increased arousability on the descending slope of early cycles together with weaker slow‑wave buffering later at depth, echoing the concept that micro‑arousals and CAP organise the ‘reversible’ nature of sleep and can gate parasomnia expression.

In fibromyalgia^26^, a general attenuation of A1/A2 suggests impaired homeostatic slow‑wave replenishment and altered arousal control, echoing reports of non‑restorative sleep in chronic pain^27^. In this tertiary-care cohort, N3% in the fibromyalgia group was similar to that of controls (Table 1), in line with evidence that macro-level slow-wave amounts can remain intact even when sleep is experienced as non-restorative. Fibromyalgia showed reductions in A1/A2 (N2) and A1 (N3). Although detailed CAP phenotypes in chronic pain require larger series, these findings fit with an interpretation of impaired homeostatic reinforcement (fewer A1 ‘delta injections’) together with a tendency toward unstable, higher‑frequency transients. Mechanistically, reduced A1 across the night could contribute to non‑restorative sleep and heightened sensory gain.

Clinically, the longitudinal A‑phase burden complements classical CAP index metrics and offers a compact summary of arousal regulation across the night. Stage‑stratified API profiles differentiate cohorts in ways that simple nightly averages miss, and may prove useful as candidate biomarkers for phenotyping. For instance, reduced A1/A2 in N2 for iRBD and NT1 with light‑sleep A1/A2 elevation in NREM parasomnia, and broad A1/A2 attenuation in fibromyalgia syndrome. Because API does not require explicit B‑phase detection, it may scale readily to large, heterogeneous datasets as a screening tool^9^, with CAP‑sequence analysis reserved for confirmatory modelling.

Taken together, the disorder‑specific nocturnal signatures support a systems‑level account in which the balance between homeostatic slow‑wave recruitment and ascending arousal pressure is tuned differently across conditions. API provides a tractable, stage‑aware read‑out of this balance. By tracking the curve rather than the average, longitudinal profiling may help subtype patients, prioritise mechanistic trials (e.g. slow‑wave–targeting neuromodulation versus agents that dampen A2/A3‑like reactivity), and develop composite biomarkers that later integrate A‑ and B‑phase dynamics.

What is new compared with prior disorder‑specific CAP work? For instance, for iRBD, as briefly mentioned, prior studies disagree on directionality of CAP rate and subtype burden; our contribution is the first multi‑disorder, longitudinal mapping of A‑phase subtype trajectories across the night, showing N2‑selective A1/A2 attenuation and persistent A1 loss in N3, expressed as A‑phase burden rather than CAP cycles. On the other hand, for narcolepsy type 1, the stage‑specific pattern (A1/A2 ↓ in N2; A3 ↓ in N1) adds temporal resolution beyond macro‑architecture and is consistent with altered arousal gating; this time‑resolved profile is, to our knowledge, not previously reported in a single‑night trajectory framework. In NREM parasomnia, early‑night A1/A2 ↑ in N1 with later‑night A1 ↓ in N3 fits state‑instability models and demonstrates directional shifts across the night rather than static averages, again, novel at this granularity. And finally, for Fibromyalgia Syndrome, overall prior CAP‑specific evidence is sparse, our finding of reduced A1/A2 (especially in N2) potentially frames pain‑related hyperarousability as a failure to recruit synchronized slow‑wave responses across the night, arguably an interpretable, testable biomarker hypothesis.

Beyond modest cohort sizes, inference is constrained by several important limitations. The retrospective, cross-sectional design, reliance on an A-phase burden metric that does not model B-phase or CAP sequence dynamics, and incomplete control for covariates; findings should therefore be read as descriptive and hypothesis-generating. Moreover, control participants were not individually matched to each disorder by age and sex; we therefore interpret between‑group differences conservatively. Also, automated A‑phase subtyping was limited to C3/C4 EEG and may miss topographic nuances. We used fixed 60‑s windows; alternative window sizes could change absolute API values. Subset checks suggested similar qualitative patterns, but these are exploratory. We did not extract harmonised spectral slow-wave activity (SWA) metrics or epoch-wise stage-by-time hypnograms from the archival PSGs; our interpretations are therefore based on CAP A-phase subtypes and macro-architectural stage proportions, not on delta-band power trajectories. Finally, analyses adjust multiplicity within clearly defined families (per stage, across A1/A2/A3). Age/sex imbalance, potential respiratory/PLM influences, and medication effects were not controlled in this retrospective design; these are acknowledged limitations.

Because API does not model B‑phases, our analyses quantify A‑phase burden rather than CAP cyclicity. B‑phases embody the return to background activity and likely carry aperiodic (1/f‑like) content; future joint estimation of A‑ and B‑phases with explicit oscillatory–aperiodic decomposition should clarify how arousal transients modulate the aperiodic ‘backdrop’ of NREM sleep^28^. Prospective studies with age/sex‑matched controls and harmonised comorbidity measures (AHI, PLMI, medication) are needed to validate diagnostic utility. Topographic analyses should map regional A‑phase fields and examine hemispheric asymmetries. Finally, linking API trajectories to outcomes, for example a phenoconversion in iRBD, symptom burden in fibromyalgia, episode frequency and severity in NREM parasomnia, will determine whether longitudinal A‑phase burden can serve as a stratification or treatment‑response biomarker.

Our exploratory, hypothesis generating, findings suggest that nocturnal evolution of A‑phase subtypes carries recognisable, disorder‑specific signatures. By embedding these observations within a principled physiology of arousal and homeostatic plasticity^29^, API‑based profiling may offer a tractable route to microstructural biomarkers that bridge neurophysiology and clinical sleep medicine.

## Supplementary Information

Below is the link to the electronic supplementary material.

Supplementary Material 1

Supplementary Material 2

## Data Availability

Due to ethical and legal restrictions, the raw clinical EEG data cannot be shared. These data include sensitive health information and are governed by the data protection policies of Guy’s and St Thomas’ (GSTT) NHS Foundation Trust and King’s College London. Requests for access to derived data or anonymized summary metrics under institutional data-governance procedures may be considered on a case-by-case basis by the corresponding author and are subject to review by the Trust’s Research and Development Office and the institutional Data Protection Officer, in accordance with GDPR and NHS research governance frameworks. We provide aggregate, group-level, stage-wise API data (api_stage_group_means.csv) to reproduce stage-stratified figures and tables. Subject-level or window-level derived data are available upon reasonable request, subject to a new ethical application under GSTT governance and GDPR.

## References

[1] Scarpetta, S. et al. Criticality of neuronal avalanches in human sleep and their relationship with sleep macro- and micro-architecture. *iScience* **26**, 107840. 10.1016/j.isci.2023.107840 (2023). 37766992 10.1016/j.isci.2023.107840 PMC10520337

[2] Halasz, P., Terzano, M., Parrino, L. & Bodizs, R. The nature of arousal in sleep. *J. Sleep Res.* **13**, 1–23 (2004). 14996030 10.1111/j.1365-2869.2004.00388.x

[3] Parrino, L., Ferri, R., Bruni, O. & Terzano, M. G. Cyclic alternating pattern (CAP): The marker of sleep instability. *Sleep Med. Rev.* **16**, 27–45. 10.1016/j.smrv.2011.02.003 (2012). 21616693 10.1016/j.smrv.2011.02.003

[4] Halász, P., Terzano, M., Parrino, L. & Bódizs, R. The nature of arousal in sleep. *J. Sleep Res.* **13**, 1–23. 10.1111/j.1365-2869.2004.00388.x (2004). 14996030 10.1111/j.1365-2869.2004.00388.x

[5] Terzano, M. G. et al. The cyclic alternating pattern as a physiologic component of normal NREM sleep. *Sleep* **8**, 137–145 (1985). 4012156 10.1093/sleep/8.2.137

[6] Parrino, L., Smerieri, A., Rossi, M. & Terzano, M. G. Relationship of slow and rapid EEG components of CAP to ASDA arousals in normal sleep. *Sleep* **24**, 881–885 (2001). 11766157 10.1093/sleep/24.8.881

[7] Terzano, M. G. et al. Atlas, rules, and recording techniques for the scoring of cyclic alternating pattern (CAP) in human sleep. *Sleep Med.* **2**, 537–553 (2001). 14592270 10.1016/s1389-9457(01)00149-6

[8] Parrino, L. et al. Atlas and updated rules for the scoring of cyclic alternating pattern (CAP) in human sleep. a consensus report by a taskforce of the European Sleep Research Society. *J. Sleep. Res.*10.1111/jsr.70283 (2026). 10.1111/jsr.70283 PMC13357963 41560629

[9] Mendonça, F., Mostafa, S. S., Morgado-Dias, F., Ravelo-García, A. G. & Rosenzweig, I. Towards automatic EEG cyclic alternating pattern analysis: A systematic review. *Biomed. Eng. Lett.* **13**, 273–291. 10.1007/s13534-023-00303-w (2023). 37519874 10.1007/s13534-023-00303-w PMC10382419

[10] Troester, M. M. et al. *The AASM Manual for the Scoring of Sleep and Associated Events: Rules, Terminology and Technical Specifications. Version 3* (American Academy of Sleep Medicine, 2023).

[11] Lüthi, A. & Nedergaard, M. Anything but small: Microarousals stand at the crossroad between noradrenaline signaling and key sleep functions. *Neuron* **113**, 509–523. 10.1016/j.neuron.2024.12.009 (2025). 39809276 10.1016/j.neuron.2024.12.009

[12] Ferri, R. et al. Effects of long-term use of clonazepam on nonrapid eye movement sleep patterns in rapid eye movement sleep behavior disorder. *Sleep Med.* **14**, 399–406. 10.1016/j.sleep.2013.01.007 (2013). 23490738 10.1016/j.sleep.2013.01.007

[13] Dagay, A. et al. Cyclic alternating pattern dynamics in individuals at risk for developing Parkinson’s Disease. *Ann. Neurol.* **98**, 136–146. 10.1002/ana.27217 (2025). 39981867 10.1002/ana.27217 PMC12174734

[14] Kutlu, A., Işeri, P., Selekler, M., Benbir, G. & Karadeniz, D. Cyclic alternating pattern analysis in REM sleep behavior disorder. *Sleep Breath.* **17**, 209–215. 10.1007/s11325-012-0675-5 (2013). 22367462 10.1007/s11325-012-0675-5

[15] Melpignano, A. et al. Isolated rapid eye movement sleep behavior disorder and cyclic alternating pattern: Is sleep microstructure a predictive parameter of neurodegeneration?. *Sleep*10.1093/sleep/zsz142 (2019). 31323084 10.1093/sleep/zsz142

[16] O’Reilly, C., Gosselin, N., Carrier, J. & Nielsen, T. Montreal Archive of Sleep Studies: An open-access resource for instrument benchmarking and exploratory research. *J. Sleep Res.* **23**, 628–635. 10.1111/jsr.12169 (2014). 24909981 10.1111/jsr.12169

[17] Sateia, M. J. International Classification of Sleep Disorders-Third Edition: Highlights and modifications. *Chest* **146**, 1387–1394. 10.1378/chest.14-0970 (2014). 25367475 10.1378/chest.14-0970

[18] Treede, R. D. et al. Chronic pain as a symptom or a disease: The IASP Classification of Chronic Pain for the International Classification of Diseases (ICD-11). *Pain* **160**, 19–27. 10.1097/j.pain.0000000000001384 (2019). 30586067 10.1097/j.pain.0000000000001384

[19] World Medical, A. World Medical Association Declaration of Helsinki: Ethical principles for medical research involving human subjects. *JAMA* **310**, 2191–2194. 10.1001/jama.2013.281053 (2013). 24141714 10.1001/jama.2013.281053

[20] Council, E. P. a. o. t. Vol. Regulation (EU) 2016/679 (ed EU). (*Official J. Eur. Union*, (2016).

[21] Mendonça, F. et al. A-phase index: An alternative view for sleep stability analysis based on automatic detection of the A-phases from the cyclic alternating pattern. *Sleep*10.1093/sleep/zsac217 (2023). 36098558 10.1093/sleep/zsac217

[22] Parrino, L. & Vaudano, A. E. The resilient brain and the guardians of sleep: New perspectives on old assumptions. *Sleep Med. Rev.* **39**, 98–107. 10.1016/j.smrv.2017.08.003 (2018). 29054694 10.1016/j.smrv.2017.08.003

[23] Poryazova, R., Werth, E., Parrino, L., Terzano, M. G. & Bassetti, C. L. Cyclic alternating pattern in narcolepsy patients and healthy controls after partial and total sleep deprivation. *Clin. Neurophysiol.* **122**, 1788–1793. 10.1016/j.clinph.2011.02.028 (2011). 21458370 10.1016/j.clinph.2011.02.028

[24] Terzano, M. G. et al. Cyclic alternating pattern (CAP) alterations in narcolepsy. *Sleep Med.* **7**, 619–626. 10.1016/j.sleep.2005.12.003 (2006). 16740406 10.1016/j.sleep.2005.12.003

[25] Guilleminault, C., Kirisoglu, C., da Rosa, A. C., Lopes, C. & Chan, A. Sleepwalking, a disorder of NREM sleep instability. *Sleep Med.* **7**, 163–170. 10.1016/j.sleep.2005.12.006 (2006). 16459139 10.1016/j.sleep.2005.12.006

[26] Berwick, R., Barker, C. & Goebel, A. The diagnosis of fibromyalgia syndrome. *Clin. Med.* **22**, 570–574. 10.7861/clinmed.2022-0402 (2022). 10.7861/clinmed.2022-0402 PMC9761415 36427885

[27] Rizzi, M. et al. Cyclic alternating pattern: a new marker of sleep alteration in patients with fibromyalgia? *J. Rhuematol.* **31**, 1193–1199 (2004). 15170935

[28] Rosenblum, Y. et al. Fractal cycles of sleep, a new aperiodic activity-based definition of sleep cycles. *eLife*10.7554/eLife.96784 (2025). 39784706 10.7554/eLife.96784 PMC11717360

[29] Halász, P., Timofeev, I. & Szűcs, A. Derailment of sleep homeostatic plasticity affects the most plastic brain systems and carries the risk of epilepsy. *J. Integr. Neurosci.* **22**, 111. 10.31083/j.jin2205111 (2023). 37735129 10.31083/j.jin2205111

