# Supplementary material for: Mapping nocturnal arousal across sleep and pain disorders
Source: Sci Rep. 2026 Mar 5;16:8668. doi: 10.1038/s41598-026-42639-0 (PMC12979684; doi:10.1038/s41598-026-42639-0)
Supplement: Supplementary file 2 — Supplementary Material 2 [file 41598_2026_42639_MOESM2_ESM.docx]

**Supplement**

This Supplement contains additional analyses.

**S1. Automated A‑phase detection.** A‑phase annotations (A1, A2, A3) were generated by a previously validated automated detector trained on expert‑scored CAP, with subject‑wise cross‑validation and performance reporting at the event level. The detector conforms to contemporary CAP scoring guidance, including duration constraints (2–60 s) and subtype definitions based on spectral composition. Automated labels were post‑filtered by manual sleep staging to exclude non‑NREM epochs and were exported as time‑stamped events for each EEG channel. Because B‑phases were not estimated, downstream metrics quantify A‑phase burden and not CAP cyclicity.

**S2. Definition of the A‑phase index (API).** For subtype k ∈ {A1, A2, A3} and a non‑overlapping 60‑s window w, the A‑phase index is the fraction of NREM time occupied by A‑phase k: APIₖ(w) = T_Aₖ,w / T_NREM,w, where T_Aₖ,w denotes the cumulative duration (s) of events of subtype k within w and T_NREM,w is the total duration (s) of NREM within w. Windows without NREM (T_NREM,w = 0) were omitted. Stage‑specific indices APIₖ(s) were computed by restricting both numerator and denominator to epochs staged as N1, N2 or N3.

**S3. Longitudinal alignment and smoothing.** To characterise nocturnal dynamics, windows were mapped to a normalised night coordinate x ∈ [0,1] from lights‑out to lights‑on. Subject‑level API trajectories were averaged within diagnostic groups at each x. For publication plots we applied light LOESS smoothing (span = 0.10; degree = 1) to the group‑mean curves to aid visual interpretation while preserving salient features; unsmoothed values were used for any statistics and numerical summaries. All ΔAPI/ΔΔAPI were computed from unsmoothed trajectories.

**S4. Statistical analysis. Primary group differences were assessed with two-sided Mann–Whitney U tests comparing each clinical cohort to controls for APIₖ within each NREM sleep stage and for stage-aggregated NREM sleep.** Bonferroni correction was applied across A1/A2/A3 within each stage family (N1, N2, N3) and separately for aggregated NREM sleep; adjusted p-values are reported together with U, z and rank-biserial r (effect size), plus 95% confidence intervals where appropriate. Longitudinal differences across the night were summarised descriptively from the trajectories and from group-level early- versus late-night contrasts. For trajectories and ΔΔAPI we did not perform time-resolved inferential testing. For ΔAPI, defined as the early–late difference between the first and last quartiles of the normalised night (Q1: t_norm ≤ 0.25; Q4: t_norm ≥ 0.75), we compared groups using Kruskal–Wallis tests for each subtype (A1–A3) and exploratory Mann–Whitney U tests versus controls, with rank-biserial r as the effect size (Supplementary Table S20). As a robustness check, we also applied Benjamini–Hochberg FDR across the full set of stage×subtype×channel tests per disorder; the key N2 A1/A2 reductions (iRBD, NT1, fibromyalgia), parasomnia N1 A1/A2 increases (C3), and iRBD N3 A3 (C3) remained significant at q < 0.05.

**S5. Robustness and sensitivity analyses.** We repeated the stage‑wise comparisons separately for C3 and C4 to probe hemispheric asymmetry and summarised paired C3–C4 differences using Wilcoxon signed‑rank tests within groups. We confirmed that the principal patterns, attenuated A1/A2 in iRBD, NT1 and FMS; preserved A3, were unchanged. Classifier outputs were visually spot‑checked against manual CAP‑A detections in a subset (iRBD, parasomnia), revealing no systematic subtype‑specific biases in API estimates.

**S6. Data sharing & reproducibility note.**
We deposit a de‑identified dataset of A‑phase index values (A1/A2/A3) for every NREM sleep stage and channel (no raw EEG). All inferential statistics are computed on unsmoothed values; LOESS smoothing (span = 0.10) appears only in trajectory displays.

## **S7. Key N2 A1/A2 contrasts.**

For each disorder and channel, we report Control and Group means (API), absolute and percent differences, Glass’s Δ (using the Control SD), and the Bonferroni-adjusted Mann–Whitney p-values drawn from the channel-specific supplementary tables.

| **Group** | **Channel** | **Stage** | **Subtype** | **Control Mean** | **Group Mean** | **Difference** | **% Difference** | **Glass's Δ (vs Ctrl)** | **Mann–Whitney p_adj** |
| --- | --- | --- | --- | --- | --- | --- | --- | --- | --- |
| Fibromyalgia | C3 | N2 | A1 | 0.028 | 0.011 | -0.017 | -60.7 | -0.9 | 0.001 |
| Fibromyalgia | C3 | N2 | A2 | 0.129 | 0.05 | -0.079 | -61.2 | -1.78 | 4.055e-05 |
| Fibromyalgia | C4 | N2 | A1 | 0.027 | 0.011 | -0.016 | -59.3 | -0.84 | 0.001 |
| Fibromyalgia | C4 | N2 | A2 | 0.126 | 0.05 | -0.076 | -60.3 | -1.72 | 7.769e-05 |
| NT1 | C3 | N2 | A1 | 0.028 | 0.013 | -0.015 | -53.6 | -0.79 | 0.002 |
| NT1 | C3 | N2 | A2 | 0.129 | 0.053 | -0.076 | -58.9 | -1.72 | 2.551e-06 |
| NT1 | C4 | N2 | A1 | 0.027 | 0.012 | -0.015 | -55.6 | -0.79 | 0.003 |
| NT1 | C4 | N2 | A2 | 0.126 | 0.06 | -0.066 | -52.4 | -1.49 | 5.327e-06 |
| iRBD | C3 | N2 | A1 | 0.028 | 0.014 | -0.014 | -50.0 | -0.74 | 0.001 |
| iRBD | C3 | N2 | A2 | 0.129 | 0.055 | -0.074 | -57.4 | -1.67 | 2.259e-06 |
| iRBD | C4 | N2 | A1 | 0.027 | 0.013 | -0.014 | -51.9 | -0.74 | 0.002 |
| iRBD | C4 | N2 | A2 | 0.126 | 0.053 | -0.073 | -57.9 | -1.65 | 1.68e-06 |

## **S8. N1 A1/A2 parasomnia signal (by channel).**

Channel-resolved light-sleep (N1) contrasts for NREM parasomnia vs Control. Values are computed from the published channel-specific tables; Glass’s Δ uses Control SD derived from its SE (n=40).

| **Group** | **Channel** | **Stage** | **Subtype** | **Control Mean** | **Group Mean** | **Difference** | **% Difference** | **Glass's Δ (vs Ctrl)** | **Mann–Whitney p_adj** |
| --- | --- | --- | --- | --- | --- | --- | --- | --- | --- |
| NREM parasomnia | C3 | N1 | A1 | 0.001 | 0.005 | 0.004 | 400.0 | 0.63 | 0.001023 |
| NREM parasomnia | C3 | N1 | A2 | 0.007 | 0.013 | 0.006 | 85.7 | 0.95 | 0.016 |
| NREM parasomnia | C4 | N1 | A1 | 0.001 | 0.003 | 0.002 | 200.0 | 0.32 | 0.167 |
| NREM parasomnia | C4 | N1 | A2 | 0.007 | 0.012 | 0.005 | 71.4 | 0.79 | 0.073 |

## **Data stub deposited for reproducibility**

We provide a machine-readable CSV of stage-wise group means (A1/A2/A3 for N1/N2/N3; C3/C4; all groups) derived from the manuscript’s supplementary tables. This enables re-plotting stage-stratified profiles and reproduces Supplementary Tables S7–S8 herein. File: api_stage_group_means.csv

**Supplementary Table S1.** Demographics and Sleep Macrostructure Parameters in Fibromyalgia and Control Groups according to AASM Scoring Guidelines

|  |  |  | **Control** | | |  |  | **Fibromyalgia** | | | **Statistics** | |
| --- | --- | --- | --- | --- | --- | --- | --- | --- | --- | --- | --- | --- |
|  |  |  |  | | |  |  |  | | |  | |
| **Gender (Male/Female)** | 20/20 |  |  |  | | 12/1 |  |  |  | |  |  |
|  | **n** |  |  | **%** |  | **n** |  |  | **%** |  | **χ^2^** | ***p*** |
| **Gender (Female)** | 20 |  |  | 50 |  | 12 |  |  | 92.3 |  | 7.341 | **.007*** |
|  | **Mean** | **SD** | **Median** | **IQR1** | **IQR3** | **Mean** | **SD** | **Median** | **IQR1** | **IQR3** | **U** | **P** |
|  |  |  |  |  |  |  |  |  |  |  |  |  |
| **Age (years)** | 46.175 | 16.389 | 54.000 | 27.000 | 59.750 | 44.539 | 6.118 | 44.000 | 41.500 | 49.500 | 203.500 | .242 |
| **TST (mins)** | 420.745 | 35.620 | 429.250 | 398.750 | 445.750 | 348.554 | 84.633 | 355.000 | 270.750 | 420.850 | 132.000 | **.008*** |
| **WASO (mins)** | 59.278 | 39.135 | 58.600 | 28.050 | 85.700 | 101.723 | 63.678 | 70.600 | 56.000 | 158.000 | 155.000 | **.030*** |
| **SO (mins)** | 21.555 | 13.007 | 19.650 | 10.875 | 29.250 | 27.754 | 35.954 | 15.900 | 4.200 | 39.250 | 233.000 | .577 |
| **SE (%)** | 84.370 | 8.239 | 83.650 | 79.325 | 91.075 | 72.915 | 15.605 | 80.100 | 55.200 | 85.450 | 151.000 | **.024*** |
| **SL to N1 (mins)** | 21.555 | 13.007 | 19.650 | 10.875 | 29.250 | 27.754 | 35.954 | 15.900 | 4.200 | 39.250 | 233.000 | .577 |
| **SL to N2 (mins)** | 26.080 | 13.548 | 24.350 | 15.950 | 32.475 | 41.946 | 46.227 | 21.500 | 11.000 | 53.000 | 247.000 | .788 |
| **SL to N3 (mins)** | 42.868 | 18.557 | 41.100 | 28.500 | 56.950 | 61.600 | 52.399 | 39.000 | 22.000 | 102.450 | 249.500 | .828 |
| **SL to REM (mins)** | 81.600 | 29.847 | 71.750 | 63.125 | 98.875 | 90.458 | 39.9223 | 85.000 | 58.875 | 101.000 | 221.500 | .688 |
| **AHI (Ev/Hr)** | 1.850 | 0.700 | 2.000 | 1.000 | 2.000 | 1.408 | 1.624 | 0.500 | 0.100 | 2.950 | 179.000 | .082 |
| **Arousal Index (/hr)** | 11.563 | 3.963 | 11.500 | 8.700 | 13.475 | 20.692 | 9.784 | 18.700 | 12.650 | 27.750 | 100.000 | **.001*** |
| **N1 % of TST** | 8.835 | 4.116 | 7.350 | 5.800 | 12.075 | 14.039 | 12.514 | 9.900 | 7.150 | 17.800 | 175.500 | .081 |
| **N2 % of TST** | 47.160 | 7.036 | 45.900 | 41.525 | 54.050 | 40.008 | 7.912 | 40.600 | 33.250 | 46.300 | 141.000 | **.014*** |
| **N3 % of TST** | 21.250 | 6.014 | 20.250 | 16.275 | 25.800 | 24.908 | 7.464 | 23.600 | 19.050 | 30.000 | 182.500 | .109 |
| **R % of TST** | 22.743 | 5.046 | 23.300 | 19.175 | 26.975 | 21.046 | 6.799 | 21.200 | 20.250 | 25.250 | 235.000 | .605 |
| **Mean SpO2 (%)** | - |  |  | - | - | 95.454 | 1.251 | 95.100 | 94.500 | 96.650 | - | - |
| **Nadir SpO2 (%)** | - |  |  | - | - | 91.462 | 2.470 | 91.000 | 90.500 | 93.500 | - | - |

***Notes****:* ***** (in bold) denotes statistically significant differences (p < 0.05). ***Abbreviations:*** **AHI**, apnoea hypopnea index; **IQR1**; first quartile, **IQR3,** third quartile; **N,** non-rapid eye movement; **ODI**, oxygen desaturation index; **R**, REM sleep; **REM,** rapid eye movement; **SD**, standard deviation, **SE**, sleep efficiency; **SL**, sleep latency; **SO,** sleep onset; **TST**, total sleep time; **WASO**, wakefulness after sleep onset. Comparison was performed with the Mann-Whitney U test. Comparison of the gender distribution between groups was analysed using Chi-Square test.

**Supplementary Table S2.** A-phase Index (API) across NREM sleep stages in fibromyalgia patients compared with healthy controls, channel C3

| **API**  **(C3)** |  | **Control** | | | | **Fibromyalgia** | | | | **Statistics** | | | | |
| --- | --- | --- | --- | --- | --- | --- | --- | --- | --- | --- | --- | --- | --- | --- |
|  |  | **Mean** | **SE** | **95% CI lower** | **95% CI upper** | **Mean** | **SE** | **95% CI lower** | **95% CI upper** | **U** | **z** | **r² (from Mann–Whitney z)** | **p** | **adj p** |
| **NREM 1** |  | | | | | | | | | | | | | |
| **A1** |  | 0.001 | 0.001 | 0.000 | 0.002 | 0.002 | 0.001 | 0.000 | 0.003 | 207.000 | -1.096 | 0.023 | .251 | .754 |
| **A2** |  | 0.007 | 0.001 | 0.004 | 0.009 | 0.007 | 0.002 | 0.002 | 0.011 | 259.000 | -0.021 | 0.000 | .992 | 1.000 |
| **A3** |  | **0.028** | **0.003** | **0.022** | **0.034** | **0.012** | **0.004** | **0.004** | **0.020** | **414.000** | **3.184** | **0.191** | **.002*** | **.005*** |
|  |  |  |  |  |  |  |  |  |  |  |  |  |  |  |
| **NREM 2** |  | | | | | | | | | | | | | |
| **A1** |  | **0.028** | **0.003** | **0.022** | **0.034** | **0.011** | **0.003** | **0.006** | **0.017** | **429.000** | **3.494** | **0.230** | **4.953E-04*** | **.001*** |
| **A2** |  | **0.129** | **0.007** | **0.115** | **0.144** | **0.050** | **0.011** | **0.026** | **0.075** | **471.000** | **4.362** | **0.359** | **1.352E-05*** | **4.055E-05*** |
| **A3** |  | 0.052 | 0.005 | 0.041 | 0.063 | 0.034 | 0.009 | 0.016 | 0.053 | 349.000 | 1.840 | 0.064 | .067 | .202 |
|  |  |  |  |  |  |  |  |  |  |  |  |  |  |  |
| **NREM 3** |  | | | | | | | | | | | | | |
| **A1** |  | **0.117** | **0.007** | **0.103** | **0.131** | **0.067** | **0.013** | **0.039** | **0.096** | **405.000** | **2.998** | **0.170** | **.003*** | **.008*** |
| **A2** |  | 0.030 | 0.004 | 0.021 | 0.038 | 0.025 | 0.005 | 0.013 | 0.037 | 280.000 | 0.413 | 0.003 | .687 | 1.000 |
| **A3** |  | 0.003 | 0.000 | 0.002 | 0.004 | 0.007 | 0.002 | 0.003 | 0.012 | 174.000 | -1.778 | 0.060 | .077 | .231 |
|  |  |  |  |  |  |  |  |  |  |  |  |  |  |  |
| **NREM** |  | | | | | | | | | | | | | |
| **A1** |  | **0.138** | **0.007** | **0.123** | **0.153** | **0.076** | **0.014** | **0.046** | **0.106** | **437.000** | **3.659** | **0.253** | **2.636E-04*** | **.001*** |
| **A2** |  | **0.151** | **0.008** | **0.135** | **0.166** | **0.073** | **0.013** | **0.044** | **0.102** | **452.000** | **3.969** | **0.297** | **7.534E-05*** | **2.260E-04*** |
| **A3** |  | 0.062 | 0.006 | 0.051 | 0.073 | 0.045 | 0.009 | 0.025 | 0.065 | 331.000 | 1.468 | 0.041 | .145 | .435 |

*Notes:* * (in bold) denotes statistically significant differences for adjusted p-values after the Bonferroni correction, *** p ≤ .05**.***Abbreviations:* adj p**, Bonferroni-adjusted p-value; **API,** A Phase Index; **C3,** channels’ codes in the standard 10-20% electroencephalography montage**; NREM,** non-rapid eye movement ;**SE,** standard error ; **U**, Mann–Whitney U test statistics; **z,** z-score; **r2,** effect size ; **95% CI lower/upper**, 95% confidence interval lower/upper. *r = z/√(n₁+n₂); r² reported.*

**Supplementary Table S3.** A-phase Index (API) across NREM sleep stages in fibromyalgia patients compared with healthy controls, channel C4

| **API**  **(C4)** |  | **Control** | | | | **Fibromyalgia** | | | | **Statistics** | | | | |
| --- | --- | --- | --- | --- | --- | --- | --- | --- | --- | --- | --- | --- | --- | --- |
|  |  | **Mean** | **SE** | **95% CI lower** | **95% CI upper** | **Mean** | **SE** | **95% CI lower** | **95% CI upper** | **U** | **z** | **r2** | **p** | **adj p** |
| **NREM 1** |  | | | | | | | | | | | | | |
| **A1** |  | 0.001 | 0.001 | 0.000 | 0.003 | 0.001 | 0.001 | 0.000 | 0.003 | 259.000 | -0.021 | 0.000 | .991 | 1.000 |
| **A2** |  | 0.007 | 0.001 | 0.004 | 0.009 | 0.008 | 0.003 | 0.002 | 0.013 | 264.500 | 0.093 | 0.000 | .934 | 1.000 |
| **A3** |  | **0.027** | **0.003** | **0.021** | **0.033** | **0.012** | **0.004** | **0.004** | **0.019** | **405.000** | **2.998** | **0.170** | **.003*** | **.008*** |
|  |  |  |  |  |  |  |  |  |  |  |  |  |  |  |
| **NREM 2** |  | | | | | | | | | | | | | |
| **A1** |  | **0.027** | **0.003** | **0.021** | **0.033** | **0.011** | **0.002** | **0.005** | **0.016** | **431.000** | **3.535** | **0.236** | **4.240E-04*** | **.001*** |
| **A2** |  | **0.126** | **0.007** | **0.112** | **0.140** | **0.050** | **0.012** | **0.024** | **0.076** | **464.000** | **4.217** | **0.336** | **2.590E-05*** | **7.769E-05*** |
| **A3** |  | 0.055 | 0.006 | 0.043 | 0.067 | 0.035 | 0.008 | 0.017 | 0.054 | 339.000 | 1.633 | 0.050 | .105 | .314 |
|  |  |  |  |  |  |  |  |  |  |  |  |  |  |  |
| **NREM 3** |  | | | | | | | | | | | | | |
| **A1** |  | **0.120** | **0.007** | **0.106** | **0.134** | **0.066** | **0.014** | **0.035** | **0.097** | **402.000** | **2.935** | **0.163** | **.003*** | **.010*** |
| **A2** |  | 0.028 | 0.004 | 0.019 | 0.036 | 0.030 | 0.008 | 0.013 | 0.047 | 240.000 | -0.413 | 0.003 | .687 | 1.000 |
| **A3** |  | 0.003 | 0.000 | 0.002 | 0.004 | 0.007 | 0.002 | 0.003 | 0.012 | 178.000 | -1.695 | 0.054 | .092 | .276 |
|  |  |  |  |  |  |  |  |  |  |  |  |  |  |  |
| **NREM** |  | | | | | | | | | | | | | |
| **A1** |  | **0.140** | **0.007** | **0.125** | **0.155** | **0.074** | **0.015** | **0.042** | **0.106** | **426.000** | **3.432** | **0.222** | **.001*** | **.002*** |
| **A2** |  | **0.147** | **0.007** | **0.132** | **0.161** | **0.078** | **0.016** | **0.042** | **0.113** | **435.000** | **3.618** | **0.247** | **3.093E-04*** | **.001*** |
| **A3** |  | 0.064 | 0.006 | 0.051 | 0.076 | 0.044 | 0.009 | 0.024 | 0.065 | 328.000 | 1.406 | 0.037 | .163 | .489 |

*Notes:* * (in bold) denotes statistically significant differences for adjusted p-values after the Bonferroni correction, *** p ≤ .05**. ***Abbreviations:* adj p**, Bonferroni-adjusted p-value; **API,** A Phase Index; **C4,** channels’ codes in the standard 10-20% electroencephalography montage**; NREM,** non-rapid eye movement ;**SE,** standard error ; **U**, Mann–Whitney U test statistics; **z,** z-score; **r2,** effect size; **95% CI lower/upper**, 95% confidence interval lower/upper

**Supplementary Table S4.** Summary of two paired Wilcoxon signed-rank tests comparing hemispheres within the fibromyalgia group

| **API** |  | **C3** | | | | **C4** | | | | **Statistics** | | | | |
| --- | --- | --- | --- | --- | --- | --- | --- | --- | --- | --- | --- | --- | --- | --- |
|  |  | **Mean** | **SE** | **95% CI lower** | **95% CI upper** | **Mean** | **SE** | **95% CI lower** | **95% CI upper** | **W** | **z** | **r2** | **p** | **adj p** |
| **NREM 1** |  | | | | | | | | | | | | | |
| **A1** |  | 0.002 | 0.001 | 0.000 | 0.003 | 0.001 | 0.001 | 0.000 | 0.003 | 14.000 | -2.201 | 0.186 | .575 | 1.000 |
| **A2** |  | 0.007 | 0.002 | 0.002 | 0.011 | 0.008 | 0.003 | 0.002 | 0.013 | 41.000 | -0.314 | 0.004 | .787 | 1.000 |
| **A3** |  | 0.012 | 0.004 | 0.004 | 0.020 | 0.012 | 0.004 | 0.004 | 0.019 | 36.000 | -0.664 | 0.017 | .542 | 1.000 |
|  |  |  |  |  |  |  |  |  |  |  |  |  |  |  |
| **NREM 2** |  | | | | | | | | | | | | | |
| **A1** |  | 0.011 | 0.003 | 0.006 | 0.017 | 0.011 | 0.002 | 0.005 | 0.016 | 28.000 | -1.223 | 0.058 | .388 | 1.000 |
| **A2** |  | 0.050 | 0.011 | 0.026 | 0.075 | 0.050 | 0.012 | 0.024 | 0.076 | 31.000 | -1.013 | 0.039 | .340 | 1.000 |
| **A3** |  | 0.034 | 0.009 | 0.016 | 0.053 | 0.035 | 0.008 | 0.017 | 0.054 | 45.000 | -0.035 | 0.000 | 1.000 | 1.000 |
|  |  |  |  |  |  |  |  |  |  |  |  |  |  |  |
| **NREM 3** |  | | | | | | | | | | | | | |
| **A1** |  | 0.067 | 0.013 | 0.039 | 0.096 | 0.066 | 0.014 | 0.035 | 0.097 | 40.000 | -0.384 | 0.006 | .735 | 1.000 |
| **A2** |  | 0.025 | 0.005 | 0.013 | 0.037 | 0.030 | 0.008 | 0.013 | 0.047 | 32.000 | -0.943 | 0.034 | .376 | 1.000 |
| **A3** |  | 0.007 | 0.002 | 0.003 | 0.012 | 0.007 | 0.002 | 0.003 | 0.012 | 42.000 | -0.245 | 0.002 | .839 | 1.000 |
|  |  |  |  |  |  |  |  |  |  |  |  |  |  |  |
| **NREM** |  | | | | | | | | | | | | | |
| **A1** |  | 0.076 | 0.014 | 0.046 | 0.106 | 0.074 | 0.015 | 0.042 | 0.106 | 37.000 | -0.594 | 0.014 | .588 | 1.000 |
| **A2** |  | 0.073 | 0.013 | 0.044 | 0.102 | 0.078 | 0.016 | 0.042 | 0.113 | 43.000 | -0.175 | 0.001 | .893 | 1.000 |
| **A3** |  | 0.045 | 0.009 | 0.025 | 0.065 | 0.044 | 0.009 | 0.024 | 0.065 | 45.000 | -0.035 | 0.000 | 1.000 | 1.000 |

*Notes:* * (in bold) denotes statistically significant differences for adjusted p-values after the Bonferroni correction, *** p ≤ .05**. ***Abbreviations:* adj p**, Bonferroni-adjusted p-value; [**C3, C4],** channels’ codes in the standard 10-20% electroencephalography montage; **NREM,** non-rapid eye movement ; **SE,** standard error ; **W,** Wilcoxon test statistic ;**z,** z-score; **r2,** effect size; **95% CI lower/upper**, 95% confidence interval lower/upper score

**Supplementary Table S5.** Demographics and Sleep Macrostructure Parameters in Narcolepsy and Control Groups according to AASM Scoring Guidelines

|  |  |  | **Control** | | |  |  | **Narcolepsy** | | | **Statistics** | |
| --- | --- | --- | --- | --- | --- | --- | --- | --- | --- | --- | --- | --- |
|  |  |  |  | | |  |  |  | | |  | |
| **Gender (Male/Female)** | 20/20 |  |  |  | | 9/10 |  |  |  | |  |  |
|  | **n** |  |  | **%** |  | **n** |  |  | **%** |  | **χ^2^** | ***p*** |
| **Gender (Female)** | 20 |  |  | 50 |  | 10 |  |  | 52.6 |  | 0.036 | .850 |
|  | **Mean** | **SD** | **Median** | **IQR1** | **IQR3** | **Mean** | **SD** | **Median** | **IQR1** | **IQR3** | **U** | **P** |
|  |  |  |  |  |  |  |  |  |  |  |  |  |
| **Age (years)** | 46.175 | 16.389 | 54.000 | 27.000 | 59.750 | 29.895 | 10.949 | 30.000 | 19.000 | 37.000 | 159.500 | **3.432E-04*** |
| **TST (mins)** | 420.745 | 35.620 | 429.250 | 398.750 | 445.750 | 413.363 | 45.322 | 409.500 | 380.000 | 458.500 | 328.500 | .403 |
| **WASO (mins)** | 59.278 | 39.135 | 58.600 | 28.050 | 85.700 | 60.147 | 27.206 | 58.700 | 31.500 | 82.600 | 353.500 | .667 |
| **SO (mins)** | 21.555 | 13.007 | 19.650 | 10.875 | 29.250 | 6.547 | 4.675 | 5.500 | 3.000 | 11.000 | 89.000 | **2.348E-06*** |
| **SE (%)** | 84.370 | 8.239 | 83.650 | 79.325 | 91.075 | 86.079 | 5.728 | 86.900 | 81.300 | 91.900 | 349.500 | .621 |
| **SL to N1 (mins)** | 21.555 | 13.007 | 19.650 | 10.875 | 29.250 | 6.547 | 4.675 | 5.500 | 3.000 | 11.000 | 89.000 | **2.348-06*** |
| **SL to N2 (mins)** | 26.080 | 13.548 | 24.350 | 15.950 | 32.475 | 10.837 | 7.222 | 9.500 | 5.000 | 18.500 | 114.000 | **1.593E-05*** |
| **SL to N3 (mins)** | 42.868 | 18.557 | 41.100 | 28.500 | 56.950 | 20.232 | 9.771 | 17.900 | 13.000 | 25.500 | 97.000 | **4.414E-06*** |
| **SL to REM (mins)** | 81.600 | 29.847 | 71.750 | 63.125 | 98.875 | 83.947 | 27.856 | 77.000 | 67.000 | 92.500 | 356.000 | .697 |
| **AHI (Ev/Hr)** | 1.850 | 0.700 | 2.000 | 1.0000 | 2.000 | 1.384 | 1.323 | 0.900 | 0.200 | 2.600 | 255.000 | **.037*** |
| **Arousal Index (/hr)** | 11.563 | 3.963 | 11.500 | 8.700 | 13.475 | 16.763 | 5.112 | 17.200 | 11.600 | 20.200 | 169.000 | **.001*** |
| **N1 % of TST** | 8.835 | 4.116 | 7.350 | 5.800 | 12.075 | 10.226 | 5.658 | 8.500 | 6.300 | 13.900 | 323.000 | .355 |
| **N2 % of TST** | 47.160 | 7.036 | 45.900 | 41.525 | 54.050 | 43.063 | 7.136 | 42.300 | 36.300 | 50.600 | 258.000 | **.048*** |
| **N3 % of TST** | 21.250 | 6.014 | 20.250 | 16.275 | 25.800 | 26.079 | 6.623 | 27.600 | 22.800 | 31.100 | 219.500 | **.009*** |
| **R % of TST** | 22.743 | 5.046 | 23.300 | 19.175 | 26.975 | 20.632 | 5.161 | 22.200 | 17.500 | 24.900 | 306.500 | .233 |
| **Mean SpO2 (%)** | - |  |  | - | - | 96.290 | 1.115 | 96.100 | 95.500 | 97.000 | - | - |
| **Nadir SpO2 (%)** | - |  |  | - | - | 90.947 | 4.223 | 92.000 | 90.000 | 94.000 | - | - |

***Notes****:* ***** (in bold) denotes statistically significant differences (p < 0.05). ***Abbreviations:*** **AHI**, apnoea hypopnea index; **IQR1**; first quartile, **IQR3,** third quartile; **N,** non-rapid eye movement; **ODI**, oxygen desaturation index; **R**, REM sleep; **REM,** rapid eye movement; **SD**, standard deviation, **SE**, sleep efficiency; **SL**, sleep latency; **SO,** sleep onset; **TST**, total sleep time; **WASO**, wakefulness after sleep onset. Comparison was performed with the Mann-Whitney U test. Comparison of the gender distribution between groups was analysed using Chi-Square test.

**Supplementary Table S6.** A-phase Index (API) across NREM sleep stages in narcolepsy patients compared with healthy controls, channel C3

| **API**  **(C3)** |  | **Control** | | | | **Narcolepsy** | | | | **Statistics** | | | | |
| --- | --- | --- | --- | --- | --- | --- | --- | --- | --- | --- | --- | --- | --- | --- |
|  |  | **Mean** | **SE** | **95% CI lower** | **95% CI upper** | **Mean** | **SE** | **95% CI lower** | **95% CI upper** | **U** | **z** | **r2** | **p** | **adj p** |
| **NREM 1** |  | | | | | | | | | | | | | |
| **A1** |  | 0.001 | 0.001 | 0.000 | 0.002 | 0.002 | 0.001 | 0.001 | 0.003 | 274.000 | -1.720 | 0.050 | .072 | .216 |
| **A2** |  | 0.007 | 0.001 | 0.004 | 0.009 | 0.005 | 0.001 | 0.002 | 0.007 | 448.000 | 1.103 | 0.021 | .274 | .821 |
| **A3** |  | **0.028** | **0.003** | **0.022** | **0.034** | **0.012** | **0.003** | **0.006** | **0.019** | **603.000** | **3.618** | **0.222** | **3.069E-04*** | **.001*** |
|  |  |  |  |  |  |  |  |  |  |  |  |  |  |  |
| **NREM 2** |  | | | | | | | | | | | | | |
| **A1** |  | **0.028** | **0.003** | **0.022** | **0.034** | **0.013** | **0.003** | **0.007** | **0.019** | **594.000** | **3.472** | **0.204** | **.001*** | **.002*** |
| **A2** |  | **0.129** | **0.007** | **0.115** | **0.144** | **0.053** | **0.009** | **0.035** | **0.071** | **684.000** | **4.932** | **0.412** | **8.504E-07*** | **2.551E-06*** |
| **A3** |  | 0.052 | 0.005 | 0.041 | 0.063 | 0.038 | 0.007 | 0.023 | 0.054 | 484.000 | 1.687 | 0.048 | .093 | .279 |
|  |  |  |  |  |  |  |  |  |  |  |  |  |  |  |
| **NREM 3** |  | | | | | | | | | | | | | |
| **A1** |  | 0.117 | 0.007 | 0.103 | 0.131 | 0.087 | 0.014 | 0.058 | 0.116 | 504.000 | 2.012 | 0.069 | **.045*** | .135 |
| **A2** |  | 0.030 | 0.004 | 0.021 | 0.038 | 0.030 | 0.007 | 0.016 | 0.045 | 380.000 | 0.000 | 0.000 | 1.000 | 1.000 |
| **A3** |  | 0.003 | 0.000 | 0.002 | 0.004 | 0.006 | 0.001 | 0.004 | 0.009 | 250.000 | -2.109 | 0.075 | **.036*** | .107 |
|  |  |  |  |  |  |  |  |  |  |  |  |  |  |  |
| **NREM** |  | | | | | | | | | | | | | |
| **A1** |  | **0.138** | **0.007** | **0.123** | **0.153** | **0.098** | **0.015** | **0.066** | **0.130** | **531.000** | **2.450** | **0.102** | **.015*** | **.044*** |
| **A2** |  | **0.151** | **0.008** | **0.135** | **0.166** | **0.080** | **0.013** | **0.053** | **0.106** | **637.000** | **4.169** | **0.295** | **3.169E-05*** | **9.507E-05*** |
| **A3** |  | 0.062 | 0.006 | 0.051 | 0.073 | 0.046 | 0.008 | 0.028 | 0.063 | 496.000 | 1.882 | 0.060 | .061 | .183 |

*Notes:* * (in bold) denotes statistically significant differences for adjusted p-values after the Bonferroni correction, *** p ≤ .05**. ***Abbreviations:* adj p**, Bonferroni-adjusted p-value; **API,** A Phase Index; **C3,** channels’ codes in the standard 10-20% electroencephalography montage**; NREM,** non-rapid eye movement ;**SE,** standard error ; **U**, Mann–Whitney U test statistics; **z,** z-score; **r2,** effect size ; **95% CI lower/upper**, 95% confidence interval lower/upper

**Supplementary Table S7.** A-phase Index (API) across NREM sleep stages in narcolepsy patients compared with healthy controls, channel C4

| **API**  **(C4)** |  | **Control** | | | | **Narcolepsy** | | | | **Statistics** | | | | |
| --- | --- | --- | --- | --- | --- | --- | --- | --- | --- | --- | --- | --- | --- | --- |
|  |  | **Mean** | **SE** | **95% CI lower** | **95% CI upper** | **Mean** | **SE** | **95% CI lower** | **95% CI upper** | **U** | **z** | **r2** | **p** | **adj p** |
| **NREM 1** |  | | | | | | | | | | | | | |
| **A1** |  | 0.001 | 0.001 | 0.000 | 0.003 | 0.003 | 0.001 | 0.000 | 0.005 | 251.000 | -2.093 | 0.074 | **.032*** | .097 |
| **A2** |  | 0.007 | 0.001 | 0.004 | 0.009 | 0.006 | 0.001 | 0.003 | 0.009 | 392.500 | 0.203 | 0.001 | .846 | 1.000 |
| **A3** |  | **0.027** | **0.003** | **0.021** | **0.033** | **0.014** | **0.003** | **0.007** | **0.020** | **561.000** | **2.936** | **0.146** | **.003*** | **.010*** |
|  |  |  |  |  |  |  |  |  |  |  |  |  |  |  |
| **NREM 2** |  | | | | | | | | | | | | | |
| **A1** |  | **0.027** | **0.003** | **0.021** | **0.033** | **0.012** | **0.003** | **0.007** | **0.018** | **586.000** | **3.342** | **0.189** | **.001*** | **.003*** |
| **A2** |  | **0.126** | **0.007** | **0.112** | **0.140** | **0.060** | **0.008** | **0.043** | **0.078** | **675.000** | **4.786** | **0.388** | **1.776E-06*** | **5.327E-06*** |
| **A3** |  | 0.055 | 0.006 | 0.043 | 0.067 | 0.036 | 0.007 | 0.022 | 0.051 | 500.000 | 1.947 | 0.064 | .053 | .158 |
|  |  |  |  |  |  |  |  |  |  |  |  |  |  |  |
| **NREM 3** |  | | | | | | | | | | | | | |
| **A1** |  | 0.120 | 0.007 | 0.106 | 0.134 | 0.087 | 0.014 | 0.058 | 0.115 | 520.000 | 2.271 | 0.087 | **.024*** | .071 |
| **A2** |  | 0.028 | 0.004 | 0.019 | 0.036 | 0.033 | 0.007 | 0.018 | 0.049 | 341.000 | -0.633 | 0.007 | .532 | 1.000 |
| **A3** |  | 0.003 | 0.000 | 0.002 | 0.004 | 0.006 | 0.002 | 0.003 | 0.010 | 288.000 | -1.492 | 0.038 | .138 | .413 |
|  |  |  |  |  |  |  |  |  |  |  |  |  |  |  |
| **NREM** |  | | | | | | | | | | | | | |
| **A1** |  | **0.140** | **0.007** | **0.125** | **0.155** | **0.097** | **0.014** | **0.067** | **0.128** | **545.000** | **2.677** | **0.121** | **.008*** | **.023*** |
| **A2** |  | **0.147** | **0.007** | **0.132** | **0.161** | **0.090** | **0.014** | **0.062** | **0.119** | **598.000** | **3.536** | **0.212** | **4.182E-04*** | **.001*** |
| **A3** |  | 0.064 | 0.006 | 0.051 | 0.076 | 0.045 | 0.008 | 0.028 | 0.062 | 497.000 | 1.898 | 0.061 | .059 | .176 |

*Notes:* * (in bold) denotes statistically significant differences for adjusted p-values after the Bonferroni correction, *** p ≤ .05*. Abbreviations:* adj p**, Bonferroni-adjusted p-value; **API,** A Phase Index; **C4,** channels’ codes in the standard 10-20% electroencephalography montage**; NREM,** non-rapid eye movement ;**SE,** standard error ; **U**, Mann–Whitney U test statistics; **z,** z-score; **r2,** effect size; **95% CI lower/upper**, 95% confidence interval lower/upper

**Supplementary** **Table S8.** Summary of two paired Wilcoxon signed-rank tests comparing hemispheres within the Narcolepsy group

| **API** |  | **C3** | | | | **C4** | | | | **Statistics** | | | | | |
| --- | --- | --- | --- | --- | --- | --- | --- | --- | --- | --- | --- | --- | --- | --- | --- |
|  |  | **Mean** | **SE** | **95% CI lower** | **95% CI upper** | **Mean** | **SE** | **95% CI lower** | **95% CI upper** | **W** | **z** | **r2** | | **p** | **adj p** |
| **NREM 1** |  | | | | | | | | | | | | | | |
| **A1** |  | 0.002 | 0.001 | 0.001 | 0.003 | 0.003 | 0.001 | 0.000 | 0.005 | 44.000 | -2.052 | 0.111 | | .594 | 1.000 |
| **A2** |  | 0.005 | 0.001 | 0.002 | 0.007 | 0.006 | 0.001 | 0.003 | 0.009 | 53.000 | -1.690 | 0.075 | | .157 | .471 |
| **A3** |  | 0.012 | 0.003 | 0.006 | 0.019 | 0.014 | 0.003 | 0.007 | 0.020 | 67.000 | -1.127 | 0.033 | | .420 | 1.000 |
|  |  |  |  |  |  |  |  |  |  |  |  |  | |  |  |
| **NREM 2** |  | | | | | | | | | | | | | | |
| **A1** |  | 0.013 | 0.003 | 0.007 | 0.019 | 0.012 | 0.003 | 0.007 | 0.018 | 93.000 | -0.080 | | 0.000 | .953 | 1.000 |
| **A2** |  | 0.053 | 0.009 | 0.035 | 0.071 | 0.060 | 0.008 | 0.043 | 0.078 | 57.000 | -1.529 | | 0.062 | .134 | .401 |
| **A3** |  | 0.038 | 0.007 | 0.023 | 0.054 | 0.036 | 0.007 | 0.022 | 0.051 | 74.000 | -0.845 | | 0.019 | .418 | 1.000 |
|  |  |  |  |  |  |  |  |  |  |  |  | |  |  |  |
| **NREM 3** |  | | | | | | | | | | | | | | |
| **A1** |  | 0.087 | 0.014 | 0.058 | 0.116 | 0.087 | 0.014 | 0.058 | 0.115 | 76.000 | -0.765 | | 0.015 | .465 | 1.000 |
| **A2** |  | 0.030 | 0.007 | 0.016 | 0.045 | 0.033 | 0.007 | 0.018 | 0.049 | 93.000 | -0.080 | | 0.000 | .953 | 1.000 |
| **A3** |  | 0.006 | 0.001 | 0.004 | 0.009 | 0.006 | 0.002 | 0.003 | 0.010 | 84.000 | -0.443 | | 0.005 | .679 | 1.000 |
|  |  |  |  |  |  |  |  |  |  |  |  | |  |  |  |
| **NREM** |  | | | | | | | | | | | | | | |
| **A1** |  | 0.098 | 0.015 | 0.066 | 0.130 | 0.097 | 0.014 | 0.067 | 0.128 | 89.000 | -0.241 | | 0.002 | .829 | 1.000 |
| **A2** |  | 0.080 | 0.013 | 0.053 | 0.106 | 0.090 | 0.014 | 0.062 | 0.119 | 54.000 | -1.650 | | 0.072 | .104 | .312 |
| **A3** |  | 0.046 | 0.008 | 0.028 | 0.063 | 0.045 | 0.008 | 0.028 | 0.062 | 85.000 | -0.402 | | 0.004 | .709 | 1.000 |

*Notes:* * (in bold) denotes statistically significant differences for adjusted p-values after the Bonferroni correction, *** p ≤ .05**. ***Abbreviations:* adj p**, Bonferroni-adjusted p-value; [**C3, C4],** channels’ codes in the standard 10-20% electroencephalography montage; **NREM,** non-rapid eye movement; **SE,** standard error ; **W,** Wilcoxon test statistic ;**z,** z-score; **r2,** effect size; **95% CI lower/upper**, 95% confidence interval lower/upper score

**Supplementary Table S9.** Demographics and Sleep Macrostructure Parameters in NREM Parasomnia and Control Groups according to AASM Scoring Guidelines

|  |  |  | **Control** | | |  |  | **NREM Parasomnia** | | | **Statistics** | |
| --- | --- | --- | --- | --- | --- | --- | --- | --- | --- | --- | --- | --- |
|  |  |  |  | | |  |  |  | | |  | |
| **Gender (Male/Female)** | 20/20 |  |  |  | | 8/10 |  |  |  | |  |  |
|  | **n** |  |  | **%** |  | **n** |  |  | **%** |  | **χ^2^** | ***p*** |
| **Gender (Female)** | 20 |  |  | 50 |  | 10 |  |  | 55.6 |  | 0.153 | .695 |
|  | **Mean** | **SD** | **Median** | **IQR1** | **IQR3** | **Mean** | **SD** | **Median** | **IQR1** | **IQR3** | **U** | **P** |
|  |  |  |  |  |  |  |  |  |  |  |  |  |
| **Age (years)** | 46.175 | 16.389 | 54.000 | 27.000 | 59.750 | 39.556 | 6.905 | 37.500 | 34.000 | 45.2500 | 282.500 | .192 |
| **TST (mins)** | 420.745 | 35.620 | 429.250 | 398.750 | 445.750 | 385.433 | 42.802 | 394.250 | 340.125 | 418.500 | 187.500 | **.004*** |
| **WASO (mins)** | 59.278 | 39.135 | 58.600 | 28.050 | 85.700 | 54.406 | 41.693 | 45.650 | 22.9750 | 77.550 | 324.000 | .545 |
| **SO (mins)** | 21.555 | 13.007 | 19.650 | 10.875 | 29.250 | 9.811 | 7.326 | 8.000 | 4.500 | 13.625 | 148.500 | **3.776E-04*** |
| **SE (%)** | 84.370 | 8.239 | 83.650 | 79.325 | 91.075 | 86.106 | 7.975 | 88.950 | 81.600 | 91.600 | 310.500 | .405 |
| **SL to N1 (mins)** | 21.555 | 13.007 | 19.650 | 10.875 | 29.250 | 9.811 | 7.326 | 8.000 | 4.500 | 13.625 | 148.500 | **3.776E-04*** |
| **SL to N2 (mins)** | 26.080 | 13.548 | 24.350 | 15.950 | 32.475 | 14.283 | 7.983 | 13.000 | 7.625 | 19.225 | 159.000 | **.001*** |
| **SL to N3 (mins)** | 42.868 | 18.557 | 41.100 | 28.500 | 56.950 | 28.117 | 12.787 | 23.650 | 18.000 | 34.850 | 181.500 | **.003*** |
| **SL to REM (mins)** | 81.600 | 29.847 | 71.750 | 63.125 | 98.875 | 86.222 | 22.210 | 81.750 | 75.750 | 101.6250 | 299.500 | .309 |
| **AHI (Ev/Hr)** | 1.850 | 0.700 | 2.000 | 1.000 | 2.000 | 1.217 | 1.822 | 0.500 | 0.300 | 1.525 | 145.000 | **2.027E-04*** |
| **Arousal Index (/hr)** | 11.563 | 3.963 | 11.500 | 8.700 | 13.475 | 15.383 | 6.441 | 14.700 | 11.475 | 19.450 | 216.500 | **.016*** |
| **N1 % of TST** | 8.835 | 4.116 | 7.350 | 5.800 | 12.075 | 8.706 | 2.800 | 9.300 | 7.175 | 10.350 | 337.000 | .699 |
| **N2 % of TST** | 47.160 | 7.036 | 45.900 | 41.525 | 54.050 | 48.128 | 8.405 | 49.800 | 42.850 | 56.400 | 320.500 | .507 |
| **N3 % of TST** | 21.250 | 6.014 | 20.250 | 16.275 | 25.800 | 21.572 | 8.22770 | 19.400 | 15.700 | 27.725 | 355.000 | .933 |
| **R % of TST** | 22.743 | 5.046 | 23.300 | 19.175 | 26.975 | 21.600 | 4.649 | 23.050 | 19.175 | 25.050 | 330.500 | .620 |
| **Mean SpO2 (%)** | - |  |  | - | - | 95.978 | 1.030 | 95.950 | 95.075 | 96.650 | - | - |
| **Nadir SpO2 (%)** | - |  |  | - | - | 92.389 | 1.195 | 92.500 | 91.750 | 93.000 | - | - |

***Notes****:* ***** (in bold) denotes statistically significant differences (p < 0.05). ***Abbreviations:*** **AHI**, apnoea hypopnea index; **IQR1**; first quartile, **IQR3,** third quartile; **N,** non-rapid eye movement; **ODI**, oxygen desaturation index; **R**, REM sleep; **REM,** rapid eye movement; **SD**, standard deviation, **SE**, sleep efficiency; **SL**, sleep latency; **SO,** sleep onset; **TST**, total sleep time; **WASO**, wakefulness after sleep onset. Comparison was performed with the Mann-Whitney U test. Comparison of the gender distribution between groups was analysed using Chi-Square test.

**Supplementary Table S10.** A-phase Index (API) across NREM sleep stages in NREM parasomnia patients compared with healthy controls, channel C3

| **API**  **(C3)** |  | **Control** | | | | **NREM Parasomnia** | | | | **Statistics** | | | | |
| --- | --- | --- | --- | --- | --- | --- | --- | --- | --- | --- | --- | --- | --- | --- |
|  |  | **Mean** | **SE** | **95% CI lower** | **95% CI upper** | **Mean** | **SE** | **95% CI lower** | **95% CI upper** | **U** | **z** | **r2** | **p** | **adj p** |
| **NREM 1** |  | | | | | | | | | | | | | |
| **A1** |  | **0.001** | **0.001** | **0.000** | **0.002** | **0.005** | **0.001** | **0.002** | **0.007** | **141.000** | **-3.471** | **0.211** | **3.411E-04*** | **1.023E-03*** |
| **A2** |  | **0.007** | **0.001** | **0.004** | **0.009** | **0.013** | **0.002** | **0.008** | **0.018** | **180.000** | **-2.791** | **0.137** | **.005*** | **.016*** |
| **A3** |  | 0.028 | 0.003 | 0.022 | 0.034 | 0.027 | 0.005 | 0.017 | 0.037 | 358.000 | 0.314 | 0.002 | .760 | 1.000 |
|  |  |  |  |  |  |  |  |  |  |  |  |  |  |  |
| **NREM 2** |  | | | | | | | | | | | | | |
| **A1** |  | 0.028 | 0.003 | 0.022 | 0.034 | 0.027 | 0.005 | 0.016 | 0.039 | 374.000 | 0.593 | 0.006 | .559 | 1.000 |
| **A2** |  | 0.129 | 0.007 | 0.115 | 0.144 | 0.106 | 0.011 | 0.083 | 0.129 | 447.000 | 1.866 | 0.061 | .063 | .190 |
| **A3** |  | 0.052 | 0.005 | 0.041 | 0.063 | 0.051 | 0.008 | 0.034 | 0.067 | 360.000 | 0.349 | 0.002 | .734 | 1.000 |
|  |  |  |  |  |  |  |  |  |  |  |  |  |  |  |
| **NREM 3** |  | | | | | | | | | | | | | |
| **A1** |  | 0.117 | 0.007 | 0.103 | 0.131 | 0.087 | 0.008 | 0.069 | 0.105 | 471.000 | 2.285 | 0.092 | **.023*** | .068 |
| **A2** |  | 0.030 | 0.004 | 0.021 | 0.038 | 0.038 | 0.008 | 0.021 | 0.055 | 296.000 | -0.767 | 0.010 | .448 | 1.000 |
| **A3** |  | 0.003 | 0.000 | 0.002 | 0.004 | 0.005 | 0.001 | 0.002 | 0.009 | 265.000 | -1.308 | 0.030 | .194 | .581 |
|  |  |  |  |  |  |  |  |  |  |  |  |  |  |  |
| **NREM** |  | | | | | | | | | | | | | |
| **A1** |  | **0.138** | **0.007** | **0.123** | **0.153** | **0.108** | **0.009** | **0.090** | **0.127** | **480.000** | **2.442** | **0.105** | **.015*** | **.045*** |
| **A2** |  | 0.151 | 0.008 | 0.135 | 0.166 | 0.135 | 0.013 | 0.107 | 0.163 | 399.000 | 1.029 | 0.019 | .308 | .923 |
| **A3** |  | 0.062 | 0.006 | 0.051 | 0.073 | 0.062 | 0.009 | 0.043 | 0.080 | 354.000 | 0.244 | 0.001 | .814 | 1.000 |

*Notes:* * (in bold) denotes statistically significant differences for adjusted p-values after the Bonferroni correction, *** p ≤ .05**. ***Abbreviations:* adj p**, Bonferroni-adjusted p-value; **API,** A Phase Index; **C3,** channels’ codes in the standard 10-20% electroencephalography montage**; NREM,** non-rapid eye movement ;**SE,** standard error ; **U**, Mann–Whitney U test statistics; **z,** z-score; **r2,** effect size ; **95% CI lower/upper**, 95% confidence interval lower/upper

**Supplementary Table S11.** A-phase Index (API) across NREM sleep stages in NREM parasomnia patients compared with healthy controls, channel C4

| **API**  **(C4)** |  | **Control** | | | | | **NREM Parasomnia** | | | | **Statistics** | | | | |
| --- | --- | --- | --- | --- | --- | --- | --- | --- | --- | --- | --- | --- | --- | --- | --- |
|  |  | **Mean** | **SE** | **95% CI lower** | | **95% CI upper** | **Mean** | **SE** | **95% CI lower** | **95% CI upper** | **U** | **z** | **r2** | **p** | **adj p** |
| **NREM 1** |  | | | | | | | | | | | | | | |
| **A1** |  | 0.001 | 0.001 | 0.000 | | 0.003 | 0.003 | 0.001 | 0.001 | 0.005 | 233.000 | -1.866 | 0.061 | .056 | .167 |
| **A2** |  | 0.007 | 0.001 | 0.004 | | 0.009 | 0.012 | 0.002 | 0.008 | 0.016 | 210.500 | -2.259 | 0.090 | **.024*** | .073 |
| **A3** |  | 0.027 | 0.003 | 0.021 | | 0.033 | 0.029 | 0.005 | 0.018 | 0.039 | 329.000 | -0.192 | 0.001 | .855 | 1.000 |
|  |  |  |  |  | |  |  |  |  |  |  |  |  |  |  |
| **NREM 2** |  | | | | | | | | | | | | | | |
| **A1** |  | 0.027 | 0.003 | | 0.021 | 0.033 | 0.024 | 0.005 | 0.013 | 0.036 | 376.000 | 0.628 | 0.007 | .536 | 1.000 |
| **A2** |  | 0.126 | 0.007 | | 0.112 | 0.140 | 0.099 | 0.014 | 0.069 | 0.129 | 448.000 | 1.884 | 0.062 | .061 | .182 |
| **A3** |  | 0.055 | 0.006 | | 0.043 | 0.067 | 0.059 | 0.011 | 0.035 | 0.083 | 329.000 | -0.192 | 0.001 | .855 | 1.000 |
|  |  |  |  | |  |  |  |  |  |  |  |  |  |  |  |
| **NREM 3** |  | | | | | | | | | | | | | | |
| **A1** |  | **0.120** | **0.007** | | **0.106** | **0.134** | **0.073** | **0.009** | **0.053** | **0.093** | **529.000** | **3.297** | **0.191** | **.001*** | **.003*** |
| **A2** |  | 0.028 | 0.004 | | 0.019 | 0.036 | 0.032 | 0.006 | 0.019 | 0.044 | 295.500 | -0.776 | 0.011 | .443 | 1.000 |
| **A3** |  | 0.003 | 0.000 | | 0.002 | 0.004 | 0.012 | 0.004 | 0.003 | 0.021 | 208.000 | -2.302 | 0.093 | **.022*** | .065 |
|  |  |  |  | |  |  |  |  |  |  |  |  |  |  |  |
| **NREM** |  | | | | | | | | | | | | | | |
| **A1** |  | **0.140** | **0.007** | | **0.125** | **0.155** | **0.090** | **0.012** | **0.065** | **0.116** | **526.000** | **3.244** | **0.185** | **.001*** | **.004*** |
| **A2** |  | 0.147 | 0.007 | | 0.132 | 0.161 | 0.123 | 0.013 | 0.096 | 0.151 | 419.000 | 1.378 | 0.033 | .171 | .513 |
| **A3** |  | 0.064 | 0.006 | | 0.051 | 0.076 | 0.076 | 0.014 | 0.046 | 0.107 | 300.000 | -0.698 | 0.009 | .491 | 1.000 |

*Notes:* * (in bold) denotes statistically significant differences for adjusted p-values after the Bonferroni correction, *** p ≤ .05**. ***Abbreviations:* adj p**, Bonferroni-adjusted p-value; **API,** A Phase Index; **C4,** channels’ codes in the standard 10-20% electroencephalography montage**; NREM,** non-rapid eye movement ;**SE,** standard error ; **U**, Mann–Whitney U test statistics; **z,** z-score; **r2,** effect size; **95% CI lower/upper**, 95% confidence interval lower/upper

**Supplementary Table S12.** Summary of two paired Wilcoxon signed-rank tests comparing hemispheres within NREM Parasomnias group

| **API** |  | **C3** | | | | **C4** | | | | **Statistics** | | | | |
| --- | --- | --- | --- | --- | --- | --- | --- | --- | --- | --- | --- | --- | --- | --- |
|  |  | **Mean** | **SE** | **95% CI lower** | **95% CI upper** | **Mean** | **SE** | **95% CI lower** | **95% CI upper** | **W** | **z** | **r2** | **p** | **adj p** |
| **NREM 1** |  | | | | | | | | | | | | | |
| **A1** |  | 0.005 | 0.001 | 0.002 | 0.007 | 0.003 | 0.001 | 0.001 | 0.005 | 23.000 | -2.533 | 0.189 | .064 | .192 |
| **A2** |  | 0.013 | 0.002 | 0.008 | 0.018 | 0.012 | 0.002 | 0.008 | 0.016 | 60.000 | -0.781 | 0.018 | .459 | 1.000 |
| **A3** |  | 0.027 | 0.005 | 0.017 | 0.037 | 0.029 | 0.005 | 0.018 | 0.039 | 52.000 | -1.160 | 0.040 | .263 | .790 |
|  |  |  |  |  |  |  |  |  |  |  |  |  |  |  |
| **NREM 2** |  | | | | | | | | | | | | | |
| **A1** |  | 0.027 | 0.005 | 0.016 | 0.039 | 0.024 | 0.005 | 0.013 | 0.036 | 62.000 | -0.686 | 0.014 | .517 | 1.000 |
| **A2** |  | 0.106 | 0.011 | 0.083 | 0.129 | 0.099 | 0.014 | 0.069 | 0.129 | 53.000 | -1.112 | 0.036 | .284 | .852 |
| **A3** |  | 0.051 | 0.008 | 0.034 | 0.067 | 0.059 | 0.011 | 0.035 | 0.083 | 46.000 | -1.444 | 0.061 | .159 | .478 |
|  |  |  |  |  |  |  |  |  |  |  |  |  |  |  |
| **NREM 3** |  | | | | | | | | | | | | | |
| **A1** |  | 0.087 | 0.008 | 0.069 | 0.105 | 0.073 | 0.009 | 0.053 | 0.093 | 55.000 | -1.018 | 0.030 | .329 | .987 |
| **A2** |  | 0.038 | 0.008 | 0.021 | 0.055 | 0.032 | 0.006 | 0.019 | 0.044 | 69.000 | -0.355 | 0.004 | .747 | 1.000 |
| **A3** |  | 0.005 | 0.001 | 0.002 | 0.009 | 0.012 | 0.004 | 0.003 | 0.021 | 31.000 | -2.154 | 0.136 | .056 | .167 |
|  |  |  |  |  |  |  |  |  |  |  |  |  |  |  |
| **NREM** |  | | | | | | | | | | | | | |
| **A1** |  | 0.108 | 0.009 | 0.090 | 0.127 | 0.090 | 0.012 | 0.065 | 0.116 | 47.000 | -1.396 | 0.057 | .174 | .523 |
| **A2** |  | 0.135 | 0.013 | 0.107 | 0.163 | 0.123 | 0.013 | 0.096 | 0.151 | 61.000 | -0.734 | 0.016 | .487 | 1.000 |
| **A3** |  | 0.062 | 0.009 | 0.043 | 0.080 | 0.076 | 0.014 | 0.046 | 0.107 | 35.000 | -1.965 | 0.114 | .051 | .152 |

*Notes:* * (in bold) denotes statistically significant differences for adjusted p-values after the Bonferroni correction, *** p ≤ .05**. ***Abbreviations:* adj p**, Bonferroni-adjusted p-value; [**C3, C4],** channels’ codes in the standard 10-20% electroencephalography montage; **NREM,** non-rapid eye movement; **SE,** standard error ; **W,** Wilcoxon test statistic ;**z,** z-score; **r2,** effect size; **95% CI lower/upper**, 95% confidence interval lower/upper score

**Supplementary Table S13.** Demographics and Sleep Macrostructure Parameters in RBD and Control Groups according to AASM Scoring Guidelines.

|  |  |  | **Control** | | |  |  | **iRBD** | | | **Statistics** | |
| --- | --- | --- | --- | --- | --- | --- | --- | --- | --- | --- | --- | --- |
|  |  |  |  | | |  |  |  | | |  | |
| **Gender (Male/Female)** | 20/20 |  |  |  | | 17/2 |  |  |  | |  |  |
|  | **n** |  |  | **%** |  | **n** |  |  | **%** |  | **χ^2^** | ***p*** |
| **Gender (Female)** | 20 |  |  | 50 |  | 2 |  |  | 10.5 |  | 8.583 | **.003*** |
|  | **Mean** | **SD** | **Median** | **IQR1** | **IQR3** | **Mean** | **SD** | **Median** | **IQR1** | **IQR3** | **U** | **P** |
|  |  |  |  |  |  |  |  |  |  |  |  |  |
| **Age (years)** | 46.175 | 16.389 | 54.000 | 27.000 | 59.750 | 60.526 | 9.680 | 60.000 | 53.000 | 70.000 | 200.500 | **.004*** |
| **TST (mins)** | 420.745 | 35.620 | 429.250 | 398.750 | 445.750 | 364.237 | 55.657 | 375.000 | 314.000 | 405.500 | 148.500 | **1.730E-04*** |
| **WASO (mins)** | 59.278 | 39.135 | 58.600 | 28.050 | 85.700 | 91.358 | 47.619 | 79.700 | 56.200 | 122.800 | 234.000 | **.018*** |
| **SO (mins)** | 21.555 | 13.007 | 19.650 | 10.875 | 29.250 | 22.695 | 25.968 | 13.900 | 7.000 | 24.000 | 302.000 | .206 |
| **SE (%)** | 84.370 | 8.239 | 83.650 | 79.325 | 91.075 | 76.416 | 11.921 | 80.900 | 63.900 | 86.600 | 242.000 | **.025*** |
| **SL to N1 (mins)** | 21.555 | 13.007 | 19.650 | 10.875 | 29.250 | 22.695 | 25.968 | 13.900 | 7.000 | 24.000 | 302.000 | .206 |
| **SL to N2 (mins)** | 26.080 | 13.548 | 24.350 | 15.950 | 32.475 | 28.011 | 27.050 | 18.400 | 9.000 | 30.500 | 315.500 | .295 |
| **SL to N3 (mins)** | 42.868 | 18.557 | 41.100 | 28.500 | 56.950 | 38.537 | 28.920 | 30.500 | 15.000 | 40.900 | 285.500 | .125 |
| **SL to REM**  **(mins)** | 81.600 | 29.847 | 71.750 | 63.125 | 98.875 | 104.947 | 74.553 | 66.500 | 59.500 | 107.500 | 367.500 | .839 |
| **AHI (Ev/Hr)** | 1.850 | 0.700 | 2.000 | 1.000 | 2.000 | 3.458 | 3.011 | 2.800 | 1.100 | 4.300 | 262.000 | **.049*** |
| **Arousal Index**  **(/hr)** | 11.563 | 3.963 | 11.500 | 8.700 | 13.475 | 20.484 | 8.013 | 19.400 | 13.800 | 25.000 | 117.500 | **2.053E-05*** |
| **N1 % of TST** | 8.835 | 4.116 | 7.350 | 5.800 | 12.075 | 11.990 | 5.502 | 11.300 | 7.700 | 16.300 | 245.000 | **.028*** |
| **N2 % of TST** | 47.160 | 7.036 | 45.900 | 41.525 | 54.050 | 42.416 | 8.765 | 42.000 | 33.400 | 50.300 | 259.000 | .050 |
| **N3 % of TST** | 21.250 | 6.014 | 20.250 | 16.275 | 25.800 | 24.821 | 6.708 | 26.400 | 20.700 | 27.900 | 254.000 | **.041*** |
| **R % of TST** | 22.743 | 5.046 | 23.300 | 19.175 | 26.975 | 20.795 | 6.446 | 20.300 | 17.000 | 24.600 | 308.500 | .246 |
| **Mean SpO2 (%)** | - |  |  | - | - | 95.200 | 1.752 | 95.400 | 93.500 | 96.300 | - | - |
| **Nadir SpO2 (%)** | - |  |  | - | - | 91.053 | 3.308 | 91.000 | 89.000 | 94.000 | - | - |

***Notes****:* ***** (in bold) denotes statistically significant differences (p < 0.05). ***Abbreviations:*** **AHI**, apnoea hypopnea index; **IQR1**; first quartile, **IQR3,** third quartile; i**RBD,** idiopathic rapid eye movement sleep behaviour disorder; **N,** non-rapid eye movement; **ODI**, oxygen desaturation index; **R**, REM sleep; **REM,** rapid eye movement; **SD**, standard deviation, **SE**, sleep efficiency; **SL**, sleep latency; **SO,** sleep onset; **TST**, total sleep time; **WASO**, wakefulness after sleep onset. Comparison was performed with the Mann-Whitney U test. Comparison of the gender distribution between groups was analysed using Chi-Square test.

**Supplementary Table S14.** A-phase Index (API) across NREM sleep stages in iRBD patients compared with healthy controls, channel C3

| **API**  **(C3)** |  | **Control** | | | | **RBD** | | | | **Statistics** | | | | |
| --- | --- | --- | --- | --- | --- | --- | --- | --- | --- | --- | --- | --- | --- | --- |
|  |  | **Mean** | **SE** | **95% CI lower** | **95% CI upper** | **Mean** | **SE** | **95% CI lower** | **95% CI upper** | **U** | **z** | **r2** | **p** | **adj p** |
| **NREM 1** |  | | | | | | | | | | | | | |
| **A1** |  | 0.001 | 0.001 | 0.000 | 0.002 | 0.003 | 0.001 | 0.001 | 0.006 | 329.000 | -0.827 | 0.012 | .379 | 1.000 |
| **A2** |  | 0.007 | 0.001 | 0.004 | 0.009 | 0.008 | 0.002 | 0.004 | 0.012 | 359.000 | -0.341 | 0.002 | .739 | 1.000 |
| **A3** |  | 0.028 | 0.003 | 0.022 | 0.034 | 0.027 | 0.007 | 0.012 | 0.042 | 461.000 | 1.314 | 0.029 | .192 | .575 |
|  |  |  |  |  |  |  |  |  |  |  |  |  |  |  |
| **NREM 2** |  | | | | | | | | | | | | | |
| **A1** |  | **0.028** | **0.003** | **0.022** | **0.034** | **0.014** | **0.004** | **0.005** | **0.023** | **596.000** | **3.504** | **0.208** | **4.724E-04*** | **.001*** |
| **A2** |  | **0.129** | **0.007** | **0.115** | **0.144** | **0.055** | **0.010** | **0.035** | **0.075** | **672.000** | **4.737** | **0.380** | **2.259E-06*** | **6.777E-06*** |
| **A3** |  | 0.052 | 0.005 | 0.041 | 0.063 | 0.061 | 0.011 | 0.038 | 0.084 | 362.000 | -0.292 | 0.001 | .776 | 1.000 |
|  |  |  |  |  |  |  |  |  |  |  |  |  |  |  |
| **NREM 3** |  | | | | | | | | | | | | | |
| **A1** |  | **0.117** | **0.007** | **0.103** | **0.131** | **0.051** | **0.009** | **0.033** | **0.070** | **653.000** | **4.429** | **0.332** | **9.834E-06*** | **2.950E-05*** |
| **A2** |  | 0.030 | 0.004 | 0.021 | 0.038 | 0.031 | 0.005 | 0.020 | 0.043 | 349.000 | -0.503 | 0.004 | .621 | 1.000 |
| **A3** |  | **0.003** | **0.000** | **0.002** | **0.004** | **0.008** | **0.002** | **0.005** | **0.012** | **201.000** | **-2.904** | **0.143** | **.004*** | **.011*** |
|  |  |  |  |  |  |  |  |  |  |  |  |  |  |  |
| **NREM** |  | | | | | | | | | | | | | |
| **A1** |  | **0.138** | **0.007** | **0.123** | **0.153** | **0.063** | **0.011** | **0.040** | **0.086** | **654.000** | **4.445** | **0.335** | **9.130E-06*** | **2.739E-05*** |
| **A2** |  | **0.151** | **0.008** | **0.135** | **0.166** | **0.083** | **0.012** | **0.057** | **0.108** | **621.000** | **3.910** | **0.259** | **9.563E-05*** | **2.869E-04*** |
| **A3** |  | 0.062 | 0.006 | 0.051 | 0.073 | 0.076 | 0.013 | 0.048 | 0.104 | 347.000 | -0.535 | 0.005 | .598 | 1.000 |

*Notes:* * (in bold) denotes statistically significant differences for adjusted p-values after the Bonferroni correction, *** p ≤ .05**. ***Abbreviations:* adj p**, Bonferroni-adjusted p-value; **API,** A Phase Index; **C3,** channels’ codes in the standard 10-20% electroencephalography montage**; NREM,** non-rapid eye movement ;i**RBD,** idiopathic rapid eye movement sleep behaviour disorder; **SE,** standard error ; **U**, Mann–Whitney U test statistics; **z,** z-score; **r2,** effect size ; **95% CI lower/upper**, 95% confidence interval lower/upper

**Supplementary Table S15.** A-phase Index (API) across NREM sleep stages in iRBD patients compared with healthy controls, channel C4.

| **API**  **(C4)** |  | **Control** | | | | **iRBD** | | | | **Statistics** | | | | |
| --- | --- | --- | --- | --- | --- | --- | --- | --- | --- | --- | --- | --- | --- | --- |
|  |  | **Mean** | **SE** | **95% CI lower** | **95% CI upper** | **Mean** | **SE** | **95% CI lower** | **95% CI upper** | **U** | **z** | **r2** | **p** | **adj p** |
| **NREM 1** |  | | | | | | | | | | | | | |
| **A1** |  | 0.001 | 0.001 | 0.000 | 0.003 | 0.003 | 0.001 | 0.000 | 0.005 | 337.000 | -0.698 | 0.008 | .475 | 1.000 |
| **A2** |  | 0.007 | 0.001 | 0.004 | 0.009 | 0.007 | 0.001 | 0.004 | 0.010 | 367.500 | -0.203 | 0.001 | .846 | 1.000 |
| **A3** |  | 0.027 | 0.003 | 0.021 | 0.033 | 0.026 | 0.007 | 0.012 | 0.040 | 466.000 | 1.395 | 0.033 | .165 | .496 |
|  |  |  |  |  |  |  |  |  |  |  |  |  |  |  |
| **NREM 2** |  | | | | | | | | | | | | | |
| **A1** |  | **0.027** | **0.003** | **0.021** | **0.033** | **0.013** | **0.004** | **0.004** | **0.021** | **592.000** | **3.439** | **0.200** | **.001*** | **.002*** |
| **A2** |  | **0.126** | **0.007** | **0.112** | **0.140** | **0.053** | **0.009** | **0.034** | **0.071** | **689.000** | **5.013** | **0.426** | **5.600E-07*** | **1.680E-06*** |
| **A3** |  | 0.055 | 0.006 | 0.043 | 0.067 | 0.060 | 0.011 | 0.036 | 0.084 | 378.000 | -0.032 | 0.000 | .981 | 1.000 |
|  |  |  |  |  |  |  |  |  |  |  |  |  |  |  |
| **NREM 3** |  | | | | | | | | | | | | | |
| **A1** |  | **0.120** | **0.007** | **0.106** | **0.134** | **0.049** | **0.009** | **0.029** | **0.068** | **669.000** | **4.688** | **0.373** | **2.868E-06*** | **8.603E-06*** |
| **A2** |  | 0.028 | 0.004 | 0.019 | 0.036 | 0.031 | 0.005 | 0.020 | 0.042 | 333.000 | -0.762 | 0.010 | .451 | 1.000 |
| **A3** |  | 0.003 | 0.000 | 0.002 | 0.004 | 0.008 | 0.002 | 0.004 | 0.012 | 233.000 | -2.385 | 0.096 | **.017*** | .052 |
|  |  |  |  |  |  |  |  |  |  |  |  |  |  |  |
| **NREM** |  | | | | | | | | | | | | | |
| **A1** |  | **0.140** | **0.007** | **0.125** | **0.155** | **0.059** | **0.011** | **0.036** | **0.083** | **668.000** | **4.672** | **0.370** | **3.103E-06*** | **9.310E-06*** |
| **A2** |  | **0.147** | **0.007** | **0.132** | **0.161** | **0.080** | **0.012** | **0.055** | **0.106** | **626.000** | **3.991** | **0.270** | **6.818E-05*** | **2.046E-04*** |
| **A3** |  | 0.064 | 0.006 | 0.051 | 0.076 | 0.073 | 0.014 | 0.045 | 0.102 | 368.000 | -0.195 | 0.001 | .852 | 1.000 |

*Notes:* * (in bold) denotes statistically significant differences for adjusted p-values after the Bonferroni correction, *** p ≤ .05**. ***Abbreviations:* adj p**, Bonferroni-adjusted p-value; **API,** A Phase Index; **C4,** channels’ codes in the standard 10-20% electroencephalography montage**; NREM,** non-rapid eye movement ;i**RBD,** idiopathic rapid eye movement sleep behaviour disorder; **SE,** standard error ; **U**, Mann–Whitney U test statistics; **z,** z-score; **r2,** effect size; **95% CI lower/upper**, 95% confidence interval lower/upper

**Supplementary Table S16.** Summary of two paired Wilcoxon signed-rank tests comparing hemispheres within iRBD group.

| **API** |  | **C3** | | | | **C4** | | | | | **Statistics** | | | | |
| --- | --- | --- | --- | --- | --- | --- | --- | --- | --- | --- | --- | --- | --- | --- | --- |
|  |  | **Mean** | **SE** | **95% CI lower** | **95% CI upper** | **Mean** | **SE** | | **95% CI lower** | **95% CI upper** | **W** | **z** | **r2** | **p** | **adj p** |
| **NREM 1** |  | | | | | | | | | | | | | | |
| **A1** |  | 0.003 | 0.001 | 0.001 | 0.006 | 0.003 | 0.001 | 0.000 | | 0.005 | 25.000 | -2.817 | 0.209 | .477 | 1.000 |
| **A2** |  | 0.008 | 0.002 | 0.004 | 0.012 | 0.007 | 0.001 | 0.004 | | 0.010 | 46.000 | -1.972 | 0.102 | .149 | .446 |
| **A3** |  | 0.027 | 0.007 | 0.012 | 0.042 | 0.026 | 0.007 | 0.012 | | 0.040 | 80.000 | -0.604 | 0.010 | .568 | 1.000 |
|  |  |  |  |  |  |  |  |  | |  |  |  |  |  |  |
| **NREM 2** |  | | | | | | | | | | | | | | |
| **A1** |  | 0.014 | 0.004 | 0.005 | 0.023 | 0.013 | 0.004 | | 0.004 | 0.021 | 56.000 | -1.569 | 0.065 | .332 | .995 |
| **A2** |  | 0.055 | 0.010 | 0.035 | 0.075 | 0.053 | 0.009 | | 0.034 | 0.071 | 72.000 | -0.926 | 0.023 | .557 | 1.000 |
| **A3** |  | 0.061 | 0.011 | 0.038 | 0.084 | 0.060 | 0.011 | | 0.036 | 0.084 | 85.000 | -0.402 | 0.004 | .709 | 1.000 |
|  |  |  |  |  |  |  |  | |  |  |  |  |  |  |  |
| **NREM 3** |  | | | | | | | | | | | | | | |
| **A1** |  | 0.051 | 0.009 | 0.033 | 0.070 | 0.049 | 0.009 | | 0.029 | 0.068 | 84.000 | -0.443 | 0.005 | .948 | 1.000 |
| **A2** |  | 0.031 | 0.005 | 0.020 | 0.043 | 0.031 | 0.005 | | 0.020 | 0.042 | 78.000 | -0.684 | 0.012 | .515 | 1.000 |
| **A3** |  | 0.008 | 0.002 | 0.005 | 0.012 | 0.008 | 0.002 | | 0.004 | 0.012 | 59.000 | -1.449 | 0.055 | .156 | .469 |
|  |  |  |  |  |  |  |  | |  |  |  |  |  |  |  |
| **NREM** |  | | | | | | | | | | | | | | |
| **A1** |  | 0.063 | 0.011 | 0.040 | 0.086 | 0.059 | 0.011 | | 0.036 | 0.083 | 77.000 | -0.724 | 0.014 | .711 | 1.000 |
| **A2** |  | 0.083 | 0.012 | 0.057 | 0.108 | 0.080 | 0.012 | | 0.055 | 0.106 | 81.000 | -0.563 | 0.008 | .595 | 1.000 |
| **A3** |  | 0.076 | 0.013 | 0.048 | 0.104 | 0.073 | 0.014 | | 0.045 | 0.102 | 76.000 | -0.765 | 0.015 | .465 | 1.000 |

*Notes:* * (in bold) denotes statistically significant differences for adjusted p-values after the Bonferroni correction, *** p ≤ .05**. ***Abbreviations:* adj p**, Bonferroni-adjusted p-value; [**C3, C4],** channels’ codes in the standard 10-20% electroencephalography montage; **NREM,** non-rapid eye movement; i**RBD** , idiopathics rapid -eye movement sleep behaviour disorder; **SE,** standard error ; **W,** Wilcoxon test statistic ;**z,** z-score; **r2,** effect size; **95% CI lower/upper**, 95% confidence interval lower/upper score

**Supplementary Table S17.** Summary of two paired Wilcoxon signed-rank tests comparing hemispheres within healthy controls.

| **API** |  | **C3** | | | | **C4** | | | | **Statistics** | | | | |
| --- | --- | --- | --- | --- | --- | --- | --- | --- | --- | --- | --- | --- | --- | --- |
|  |  | **Mean** | **SE** | **95% CI lower** | **95% CI upper** | **Mean** | **SE** | **95% CI lower** | **95% CI upper** | **W** | **z** | **r2** | **p** | **adj p** |
| **NREM 1** |  | | | | | | | | | | | | | |
| **A1** |  | 0.001 | 0.001 | 0.000 | 0.002 | 0.001 | 0.001 | 0.000 | 0.003 | 160.000 | -3.360 | 0.141 | .486 | 1.000 |
| **A2** |  | 0.007 | 0.001 | 0.004 | 0.009 | 0.007 | 0.001 | 0.004 | 0.009 | 394.000 | -0.215 | 0.001 | .837 | 1.000 |
| **A3** |  | 0.028 | 0.003 | 0.022 | 0.034 | 0.027 | 0.003 | 0.021 | 0.033 | 326.000 | -1.129 | 0.016 | .265 | .795 |
|  |  |  |  |  |  |  |  |  |  |  |  |  |  |  |
| **NREM 2** |  | | | | | | | | | | | | | |
| **A1** |  | 0.028 | 0.003 | 0.022 | 0.034 | 0.027 | 0.003 | 0.021 | 0.033 | 327.000 | -1.116 | 0.016 | .271 | .812 |
| **A2** |  | 0.129 | 0.007 | 0.115 | 0.144 | 0.126 | 0.007 | 0.112 | 0.140 | 244.000 | -2.231 | 0.062 | **.025*** | .075 |
| **A3** |  | 0.052 | 0.005 | 0.041 | 0.063 | 0.055 | 0.006 | 0.043 | 0.067 | 299.000 | -1.492 | 0.028 | .139 | .416 |
|  |  |  |  |  |  |  |  |  |  |  |  |  |  |  |
| **NREM 3** |  | | | | | | | | | | | | | |
| **A1** |  | 0.117 | 0.007 | 0.103 | 0.131 | 0.120 | 0.007 | 0.106 | 0.134 | 300.000 | -1.479 | 0.027 | .142 | .427 |
| **A2** |  | 0.030 | 0.004 | 0.021 | 0.038 | 0.028 | 0.004 | 0.019 | 0.036 | 329.000 | -1.089 | 0.015 | .283 | .848 |
| **A3** |  | 0.003 | 0.000 | 0.002 | 0.004 | 0.003 | 0.000 | 0.002 | 0.004 | 344.000 | -0.887 | 0.010 | .910 | .730 |
|  |  |  |  |  |  |  |  |  |  |  |  |  |  |  |
| **NREM** |  | | | | | | | | | | | | | |
| **A1** |  | 0.138 | 0.007 | 0.123 | 0.153 | 0.140 | 0.007 | 0.125 | 0.155 | 381.000 | -0.390 | 0.002 | .705 | 1.000 |
| **A2** |  | 0.151 | 0.008 | 0.135 | 0.166 | 0.147 | 0.007 | 0.132 | 0.161 | 284.000 | -1.694 | 0.036 | .092 | .275 |
| **A3** |  | 0.062 | 0.006 | 0.051 | 0.073 | 0.064 | 0.006 | 0.051 | 0.076 | 304.000 | -1.425 | 0.025 | .158 | .473 |

*Notes:* * (in bold) denotes statistically significant differences for adjusted p-values after the Bonferroni correction, *** p ≤ .05**. ***Abbreviations:* adj p**, Bonferroni-adjusted p-value; [**C3, C4],** channels’ codes in the standard 10-20% electroencephalography montage; **NREM,** non-rapid eye movement , **SE,** standard error ; **W,** Wilcoxon test statistic ;**z,** z-score; **r2,** effect size; **95% CI lower/upper**, 95% confidence interval lower/upper score

**Supplementary Table S18.** Summary of the two paired Wilcoxon signed-rank tests between manual and automatic scoring of A phase Index in NREM parasomnia patients .

| **NREM**  **Parasomnia**  **(n=10)** |  | **Manual** | | | | **Automatic** | | | | **Statistics** | | | | |
| --- | --- | --- | --- | --- | --- | --- | --- | --- | --- | --- | --- | --- | --- | --- |
|  |  | **Mean** | **SE** | **95% CI lower** | **95% CI upper** | **Mean** | **SE** | **95% CI lower** | **95% CI upper** | **W** | **z** | **r2** | **p** | **adj p** |
| **NREM** |  | | | | | | | | | | | | | |
| **A1** |  | 0.057 | 0.009 | 0.038 | 0.077 | 0.089 | 0.017 | 0.051 | 0.128 | 15.000 | -1.274 | 0.081 | .232 | .697 |
| **A2** |  | 0.073 | 0.008 | 0.054 | 0.092 | 0.102 | 0.019 | 0.058 | 0.146 | 18.000 | -0.968 | 0.047 | .375 | 1.000 |
| **A3** |  | 0.034 | 0.004 | 0.025 | 0.042 | 0.051 | 0.008 | 0.032 | 0.069 | 9.000 | -1.886 | 0.178 | .064 | .193 |

*Notes:* * (in bold) denotes statistically significant differences for adjusted p-values after the Bonferroni correction, *** p ≤ .05**. ***Abbreviations:* adj p**, Bonferroni-adjusted p-value**; NREM,** non-rapid eye movement ;**SE,** standard error ; **W,** Wilcoxon test statistic ;**z,** z-score; **r2,** effect size; **α,** α-value; ; **95% CI lower/upper**, 95% confidence interval lower/upper

**Supplementary Table S19.** Summary of the two paired Wilcoxon signed-rank tests between manual and automatic scoring of A phase Index in iRBD patients.

| **RBD**  **(n=6)** |  | **Manual** | | | | **Automatic** | | | | **Statistics** | | | | |
| --- | --- | --- | --- | --- | --- | --- | --- | --- | --- | --- | --- | --- | --- | --- |
|  |  | **Mean** | **SE** | **95% CI lower** | **95% CI upper** | **Mean** | **SE** | **95% CI lower** | **95% CI upper** | **W** | **z** | **r2** | **p** | **adj p** |
| **NREM** |  | | | | | | | | | | | | | |
| **A1** |  | 0.046 | 0.010 | 0.021 | 0.071 | 0.066 | 0.016 | 0.026 | 0.106 | 3.000 | -1.572 | 0.206 | .156 | .469 |
| **A2** |  | 0.050 | 0.006 | 0.036 | 0.064 | 0.081 | 0.024 | 0.019 | 0.142 | 4.000 | -1.363 | 0.155 | .219 | .656 |
| **A3** |  | 0.034 | 0.004 | 0.024 | 0.044 | 0.073 | 0.019 | 0.023 | 0.122 | 3.000 | -1.572 | 0.206 | .156 | .469 |

*Notes:* * (in bold) denotes statistically significant differences for adjusted p-values after the Bonferroni correction, *** p ≤ .05**. ***Abbreviations:* adj p**, Bonferroni-adjusted p-value**;NREM,** non-rapid eye movement ; i**RBD,** idiopathic rapid eye movement sleep behaviour disorder; **SE,** standard error ; **W,** Wilcoxon test statistic ; **z,** z-score; **r2,** effect size; **α,** α-value; ; **95% CI lower/upper**, 95% confidence interval lower/upper

**Control**


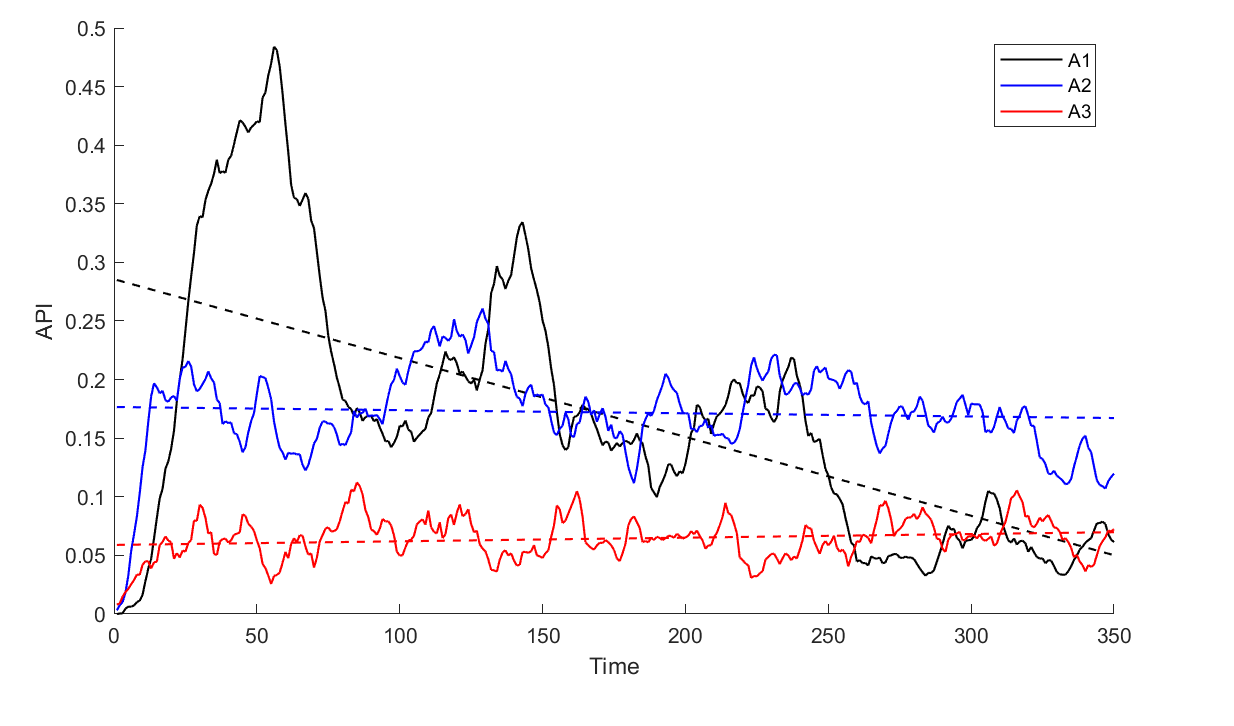


**Fibromyalgia**


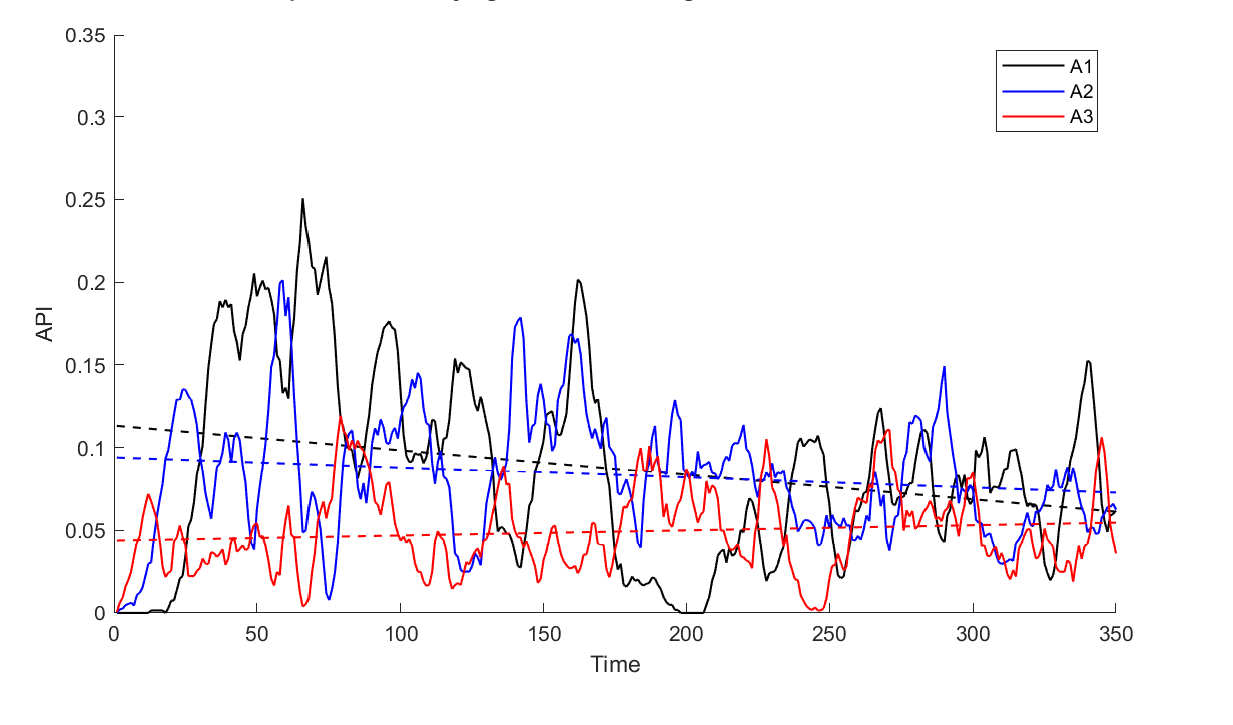


**Supplementary Figure S1**. Sleep stability profile of Control and Fibromyalgia patients visualised by the automatic API model in the C3 channel. ***Abbreviations*:** **API,** A-phase Index [$\sum_{i}^{t} =0$^A(i)^/ $\sum_{i}^{t} =0$*^N(j)^*];**C3,** channels’ codes in the standard 10-20% electroencephalography montage.

**Control**


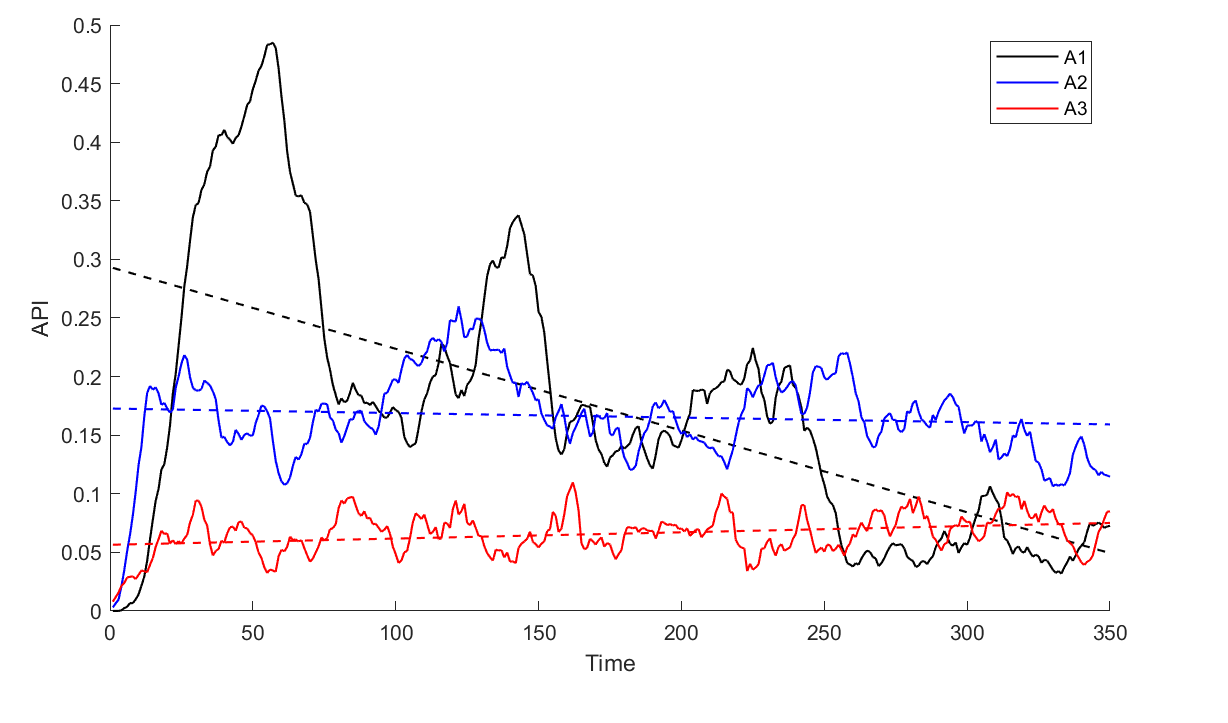


**Fibromyalgia**


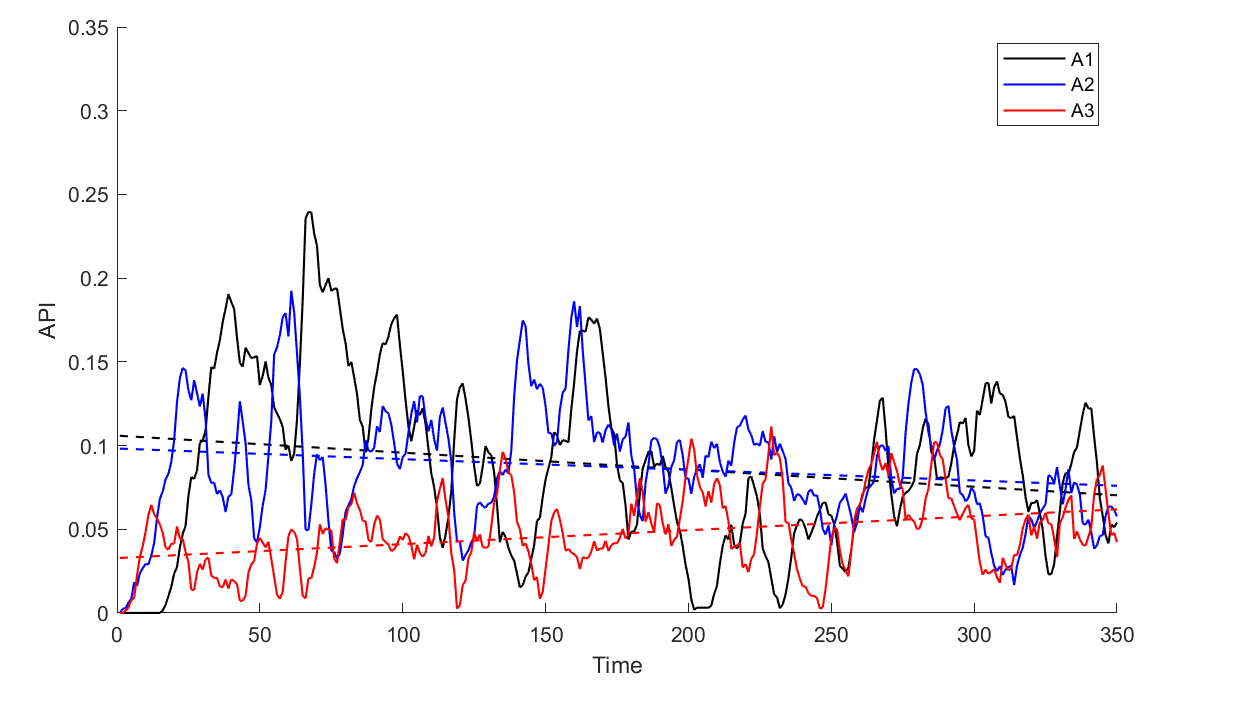


**Supplementary Figure S2.** Sleep stability profile of Control and Fibromyalgia patients visualised by the automatic API model in the C4 channel. ***Abbreviations*:** **API,** A-phase Index [$\sum_{i}^{t} =0$^A(i)^/ $\sum_{i}^{t} =0$*^N(j)^*];**C4,** channels’ codes in the standard 10-20% electroencephalography montage.

**Control**


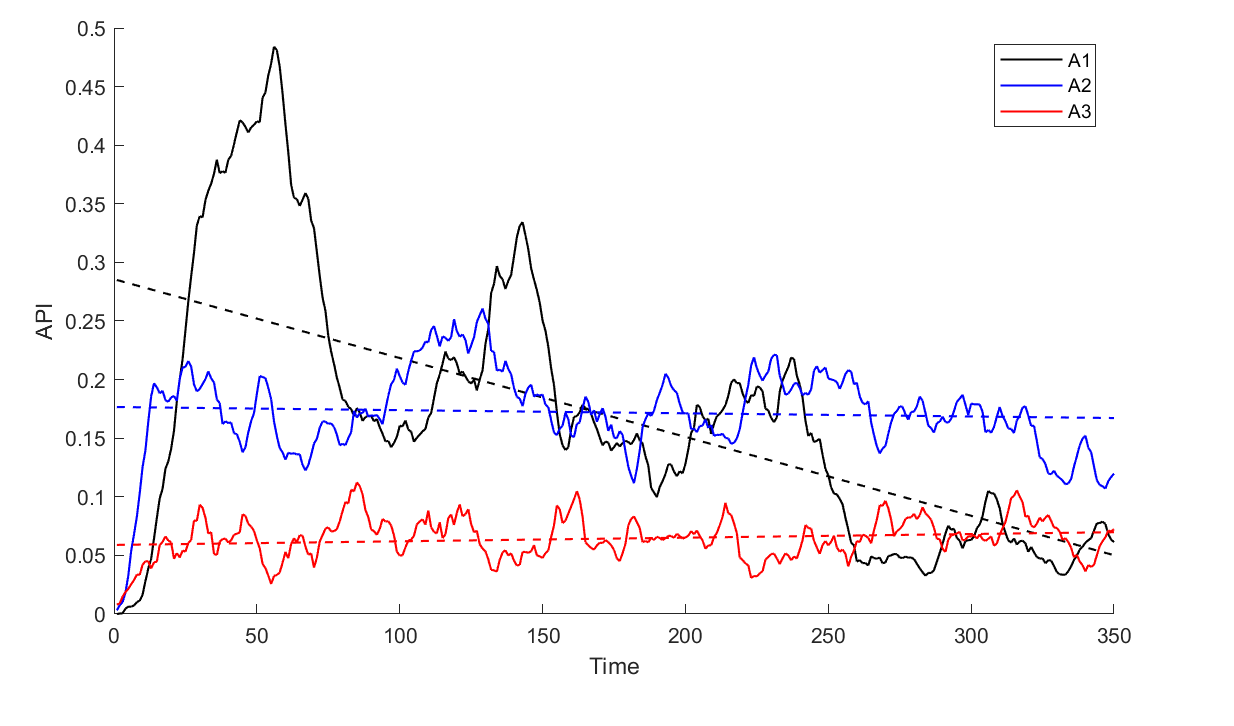


**Narcolepsy Type 1**


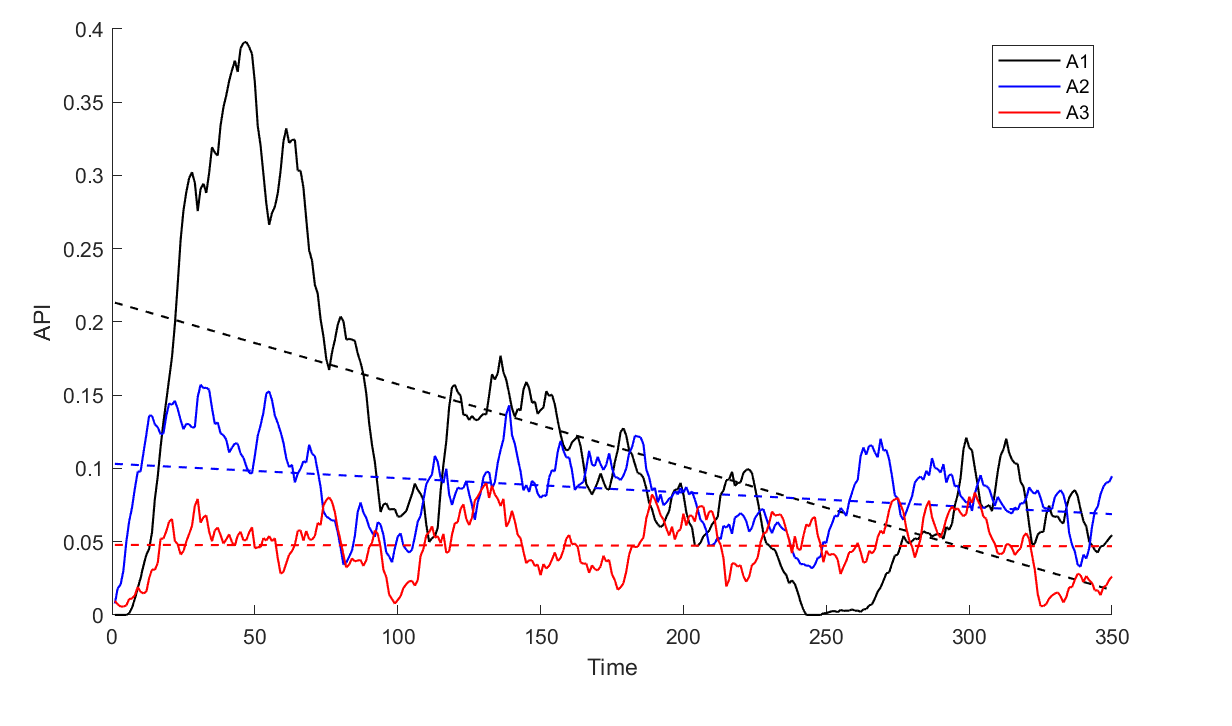


**Supplementary Figure S3.** Sleep Stability profile of Control and patients with Narcolepsy visualised by the automatic API model in the C3 channel. ***Abbreviations*:** **API,** A-phase Index: A-phase Index[$\sum_{i}^{t} =0$^A(i)^/ $\sum_{i}^{t} =0$*^N(j)^*];**C3**, channels’ codes in the standard 10-20% electroencephalography montage.

**Control**


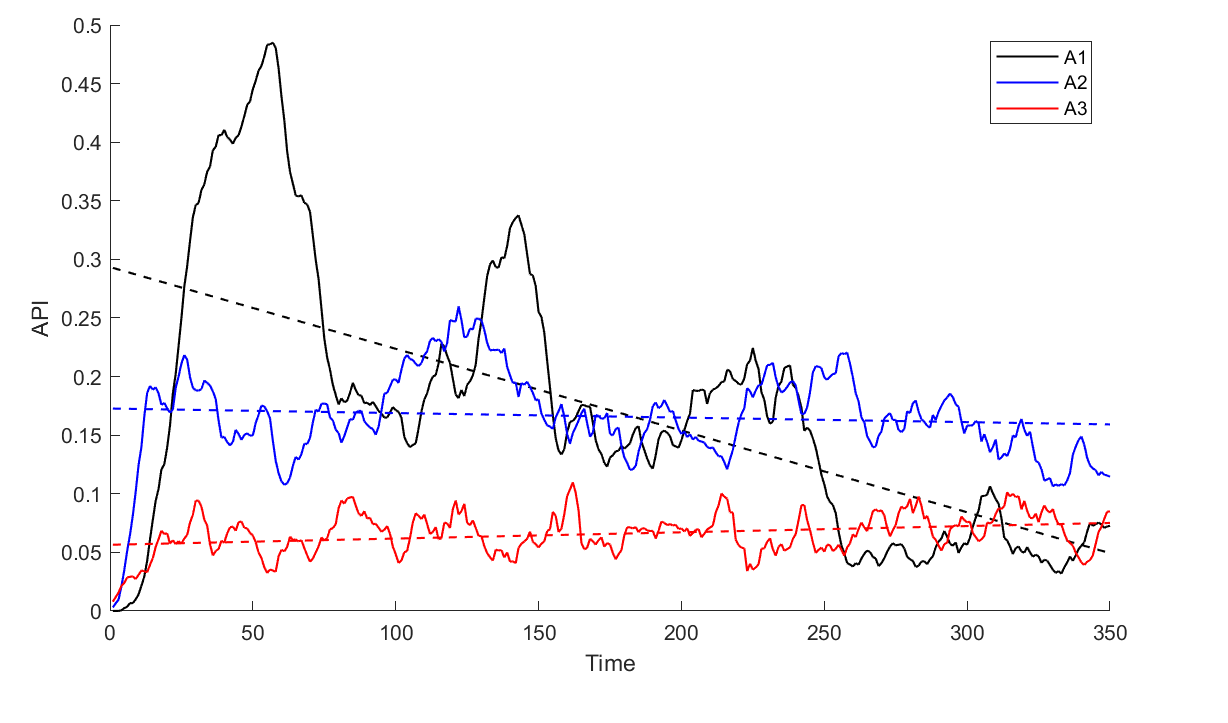


**Narcolepsy type 1**


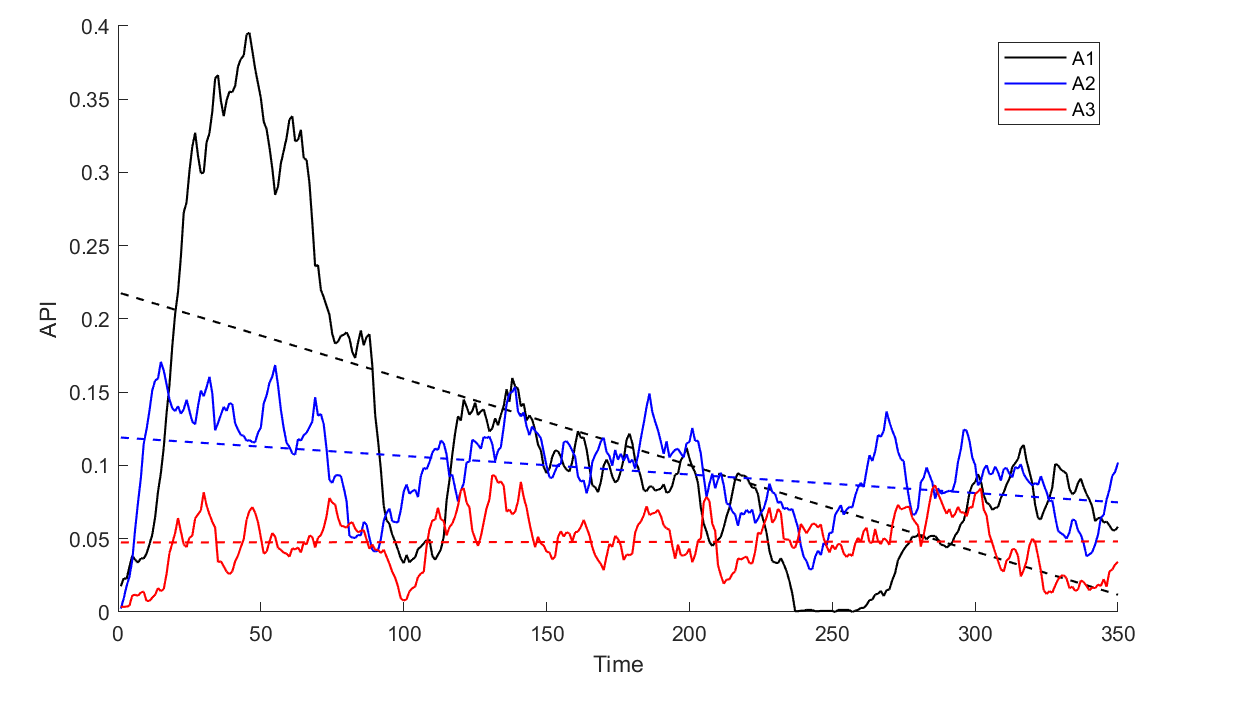


**Supplementary Figure S4.** Sleep Stability profile of Control and patients with Narcolepsy visualised by the automatic API model in the C4 channel. ***Abbreviations*:** **API,** A-phase Index: [$\sum_{i}^{t} =0$^A(i)^/ $\sum_{i}^{t} =0$*^N(j)^*];**C4**, channels’ codes in the standard 10-20% electroencephalography montage.

**Control**


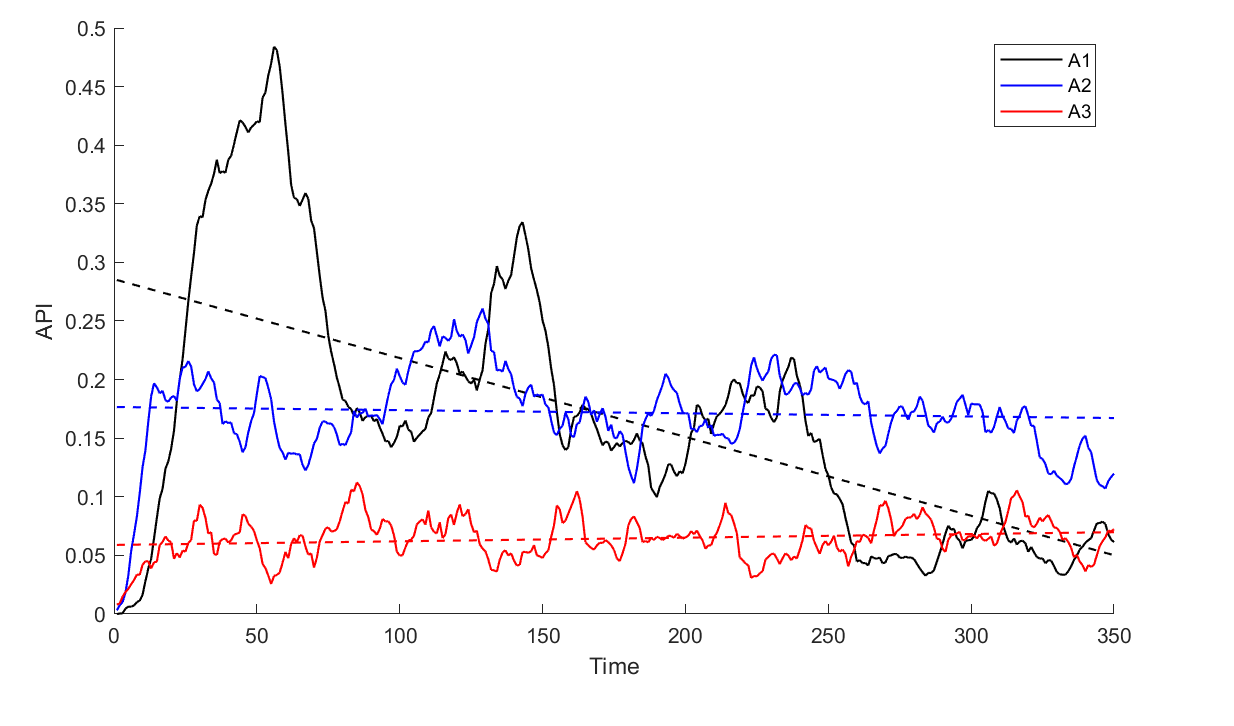


**NREM Parasomnia**


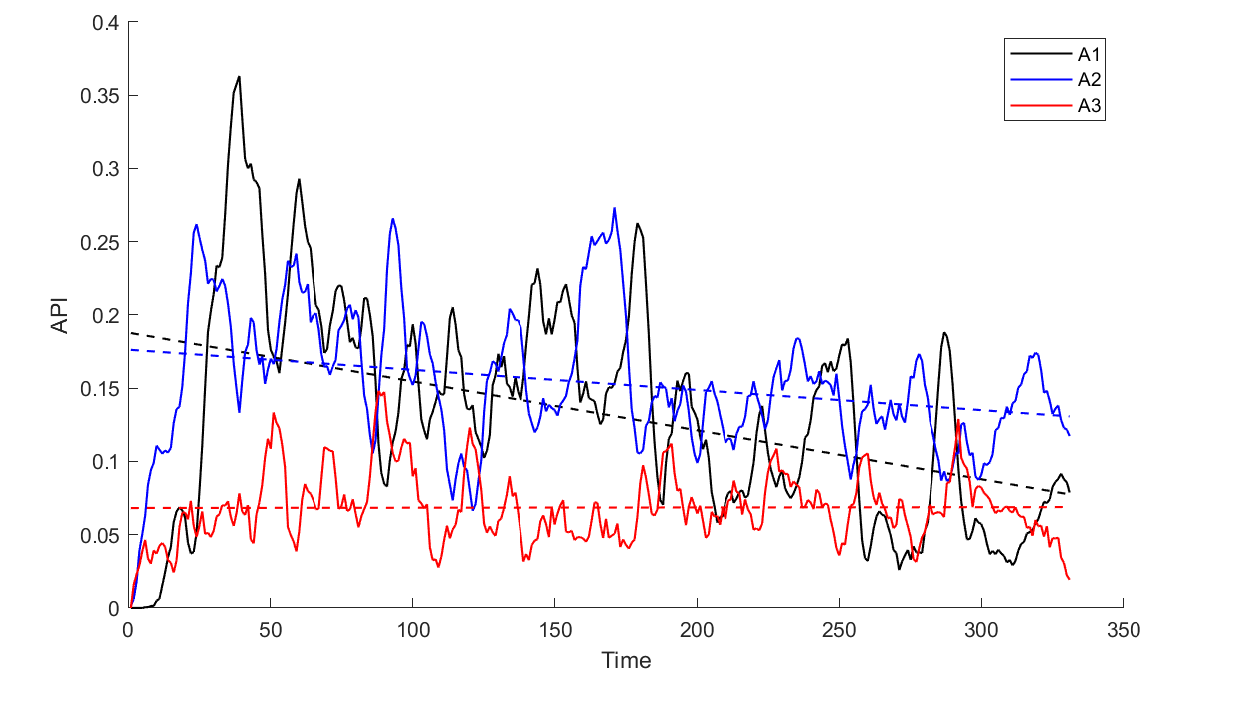


**Supplementary Figure S5.** Sleep Stability profile of Control and patients with NREM Parasomnia visualised by the automatic API model in the C3 channel. ***Abbreviations*:** **API,** A-phase Index [$\sum_{i}^{t} =0$^A(i)^/ $\sum_{i}^{t} =0$*^N(j)^*];**C3,** channels’ codes in the standard 10-20% electroencephalography montage; **NREM Parasomnia,** non-rapid eye movement sleep parasomnia

**Control**


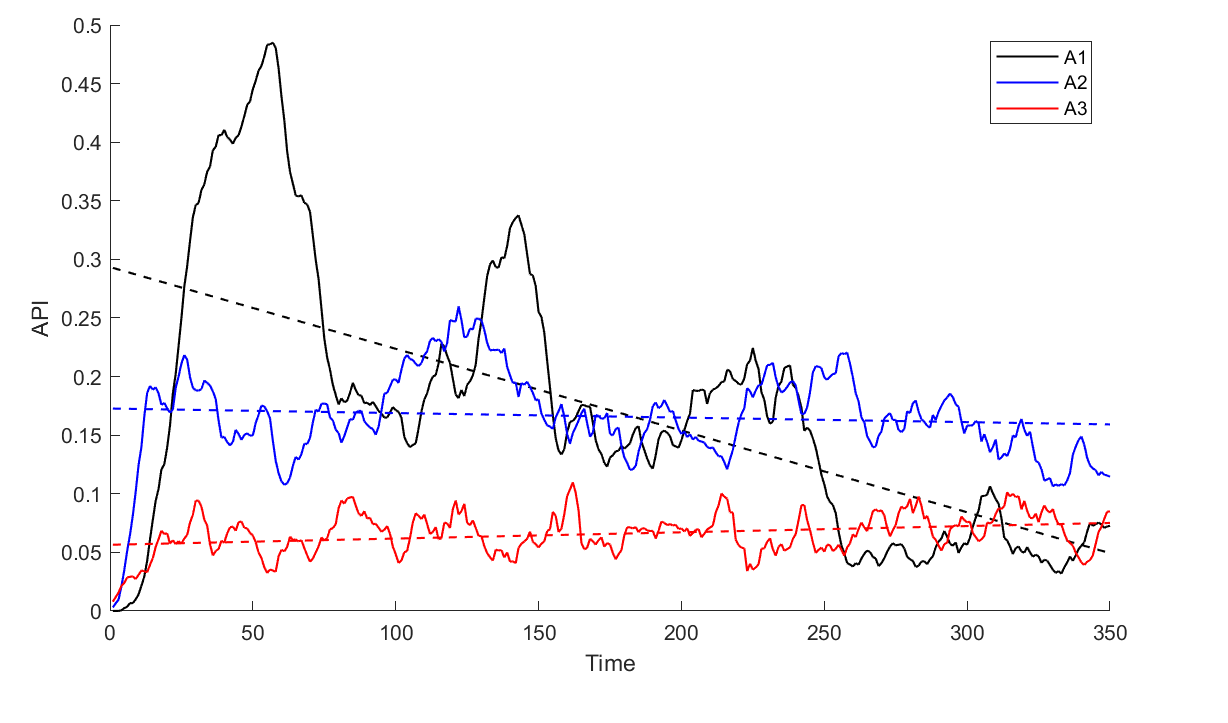


**NREM Parasomnia**


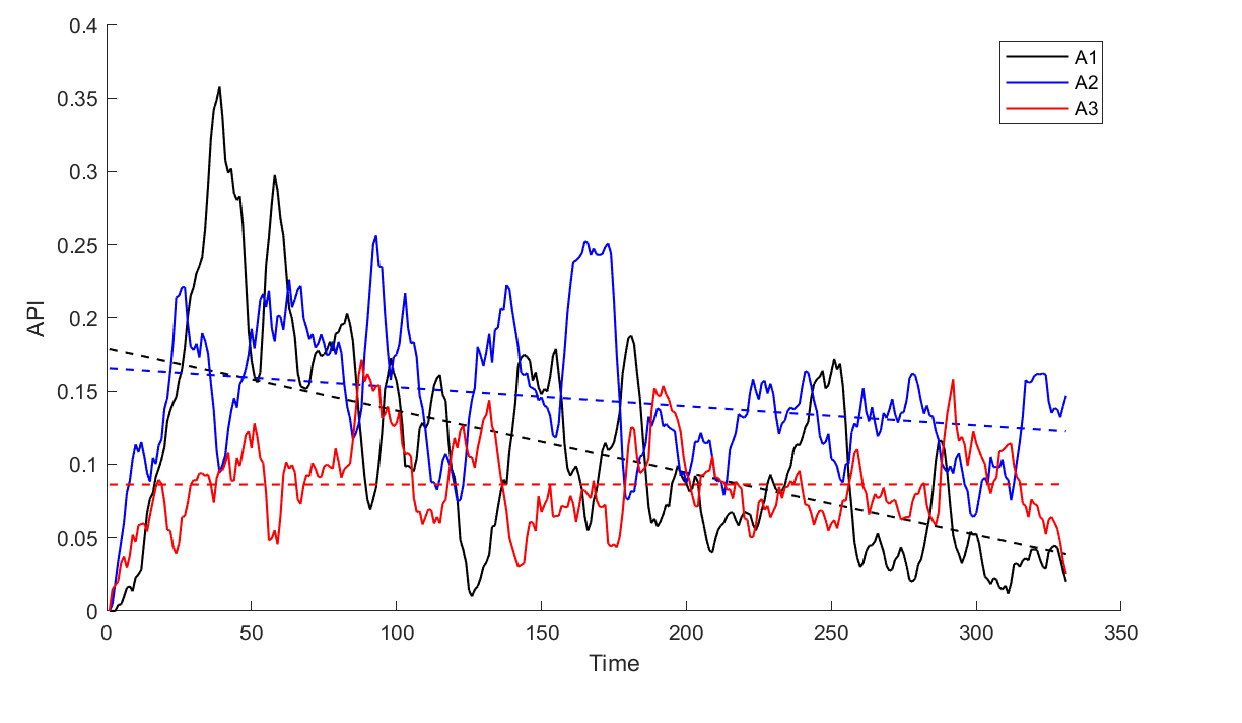


**Supplementary Figure S6.** Sleep Stability profile of Control and patients with NREM Parasomnia visualised by the automatic API model in the C4 channel. ***Abbreviations*:** **API,** A-phase Index [$\sum_{i}^{t} =0$^A(i)^/ $\sum_{i}^{t} =0$*^N(j)^*];**C4,** channels’ codes in the standard 10-20% electroencephalography montage; **NREM Parasomnia,** non-rapid eye movement sleep parasomnia

**Control**


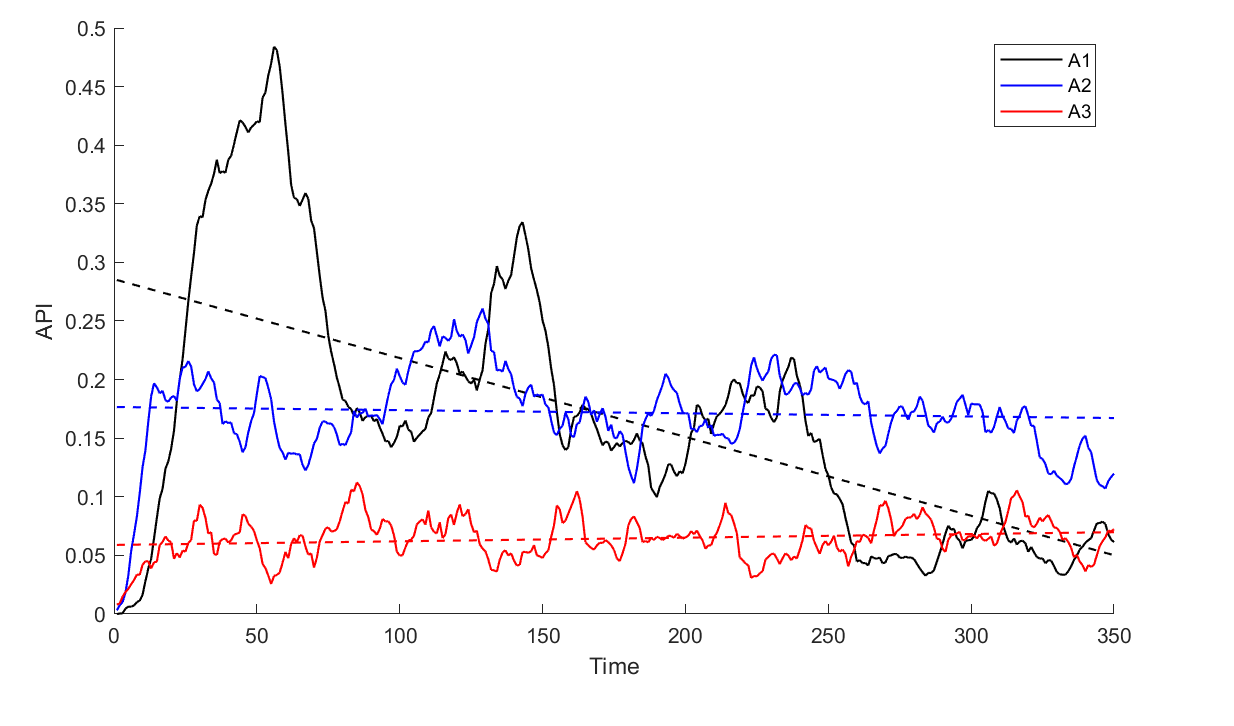


**iRBD**


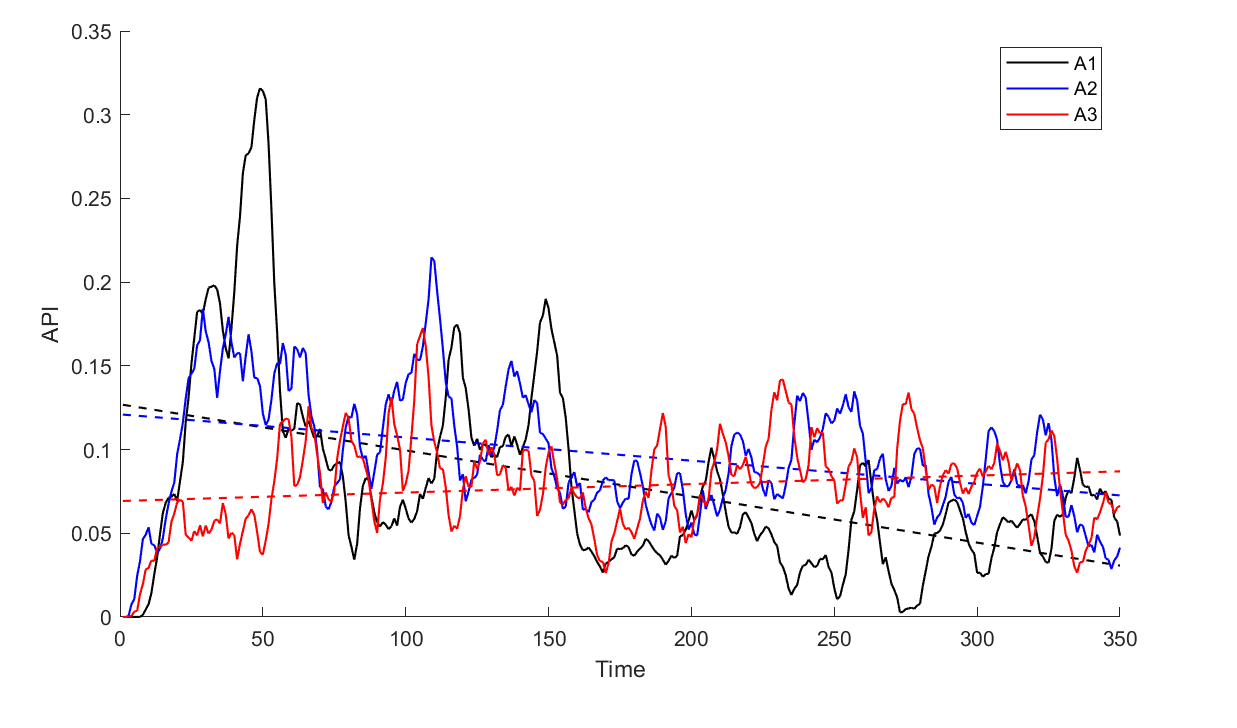


**Supplementary Figure S7.** Sleep Stability profile of Control and iRBD patients visualised by the automatic API model in the C3 channel. ***Abbreviations*:** **API,** A-phase Index [$\sum_{i}^{t} =0$^A(i)^/ $\sum_{i}^{t} =0$*^N(j)^*];**C3,** channels’ codes in the standard 10-20% electroencephalography montage; **iRBD**, idiopathic rapid -eye movement sleep behaviour disorder.

**Control**


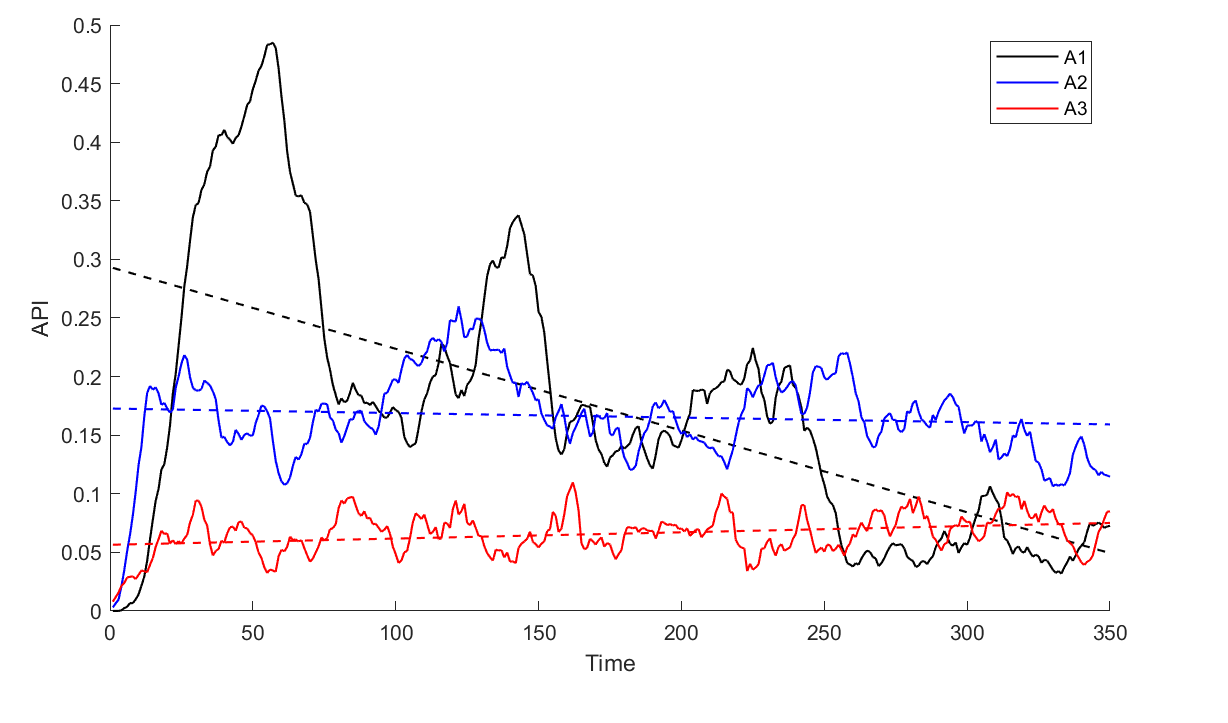


**iRBD**


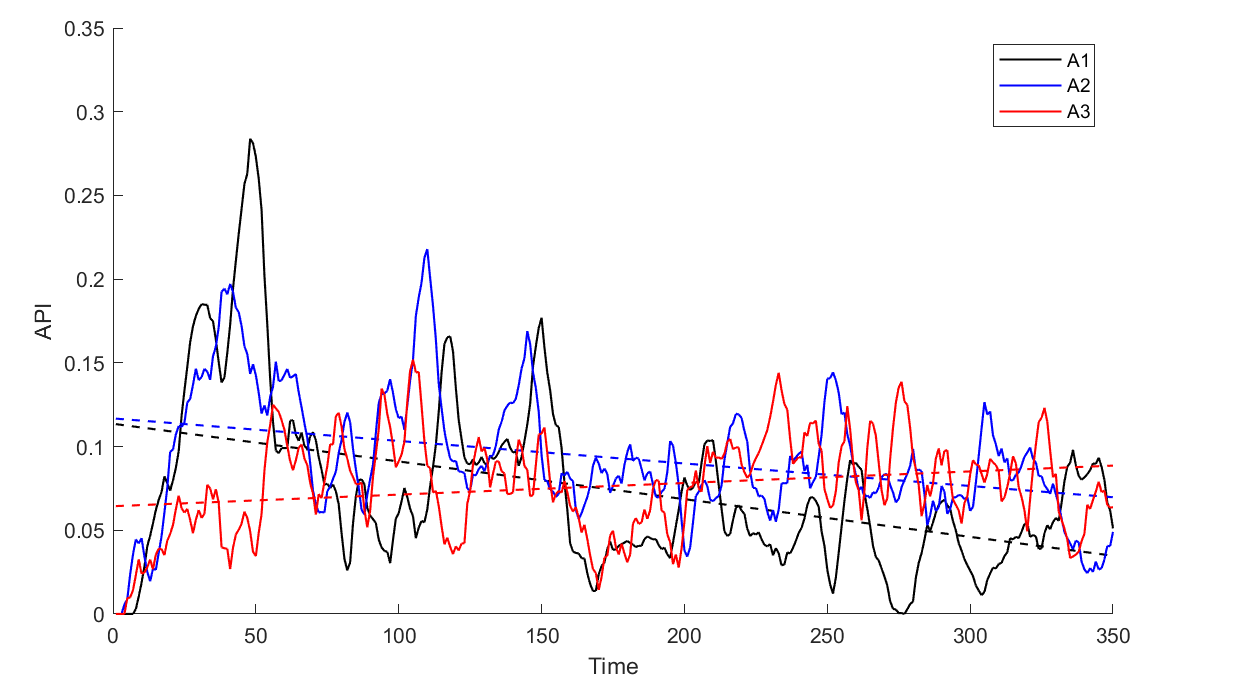


**Supplementary Figure S8.** Sleep Stability profile of Control and RBD patients visualised by the automatic API model in the C4 channel. ***Abbreviations*:** **API,** A-phase Index [$\sum_{i}^{t} =0$^A(i)^/ $\sum_{i}^{t} =0$*^N(j)^*];**C4,** channels’ codes in the standard 10-20% electroencephalography montage; **iRBD**, idiopathic rapid -eye movement sleep behaviour disorder.


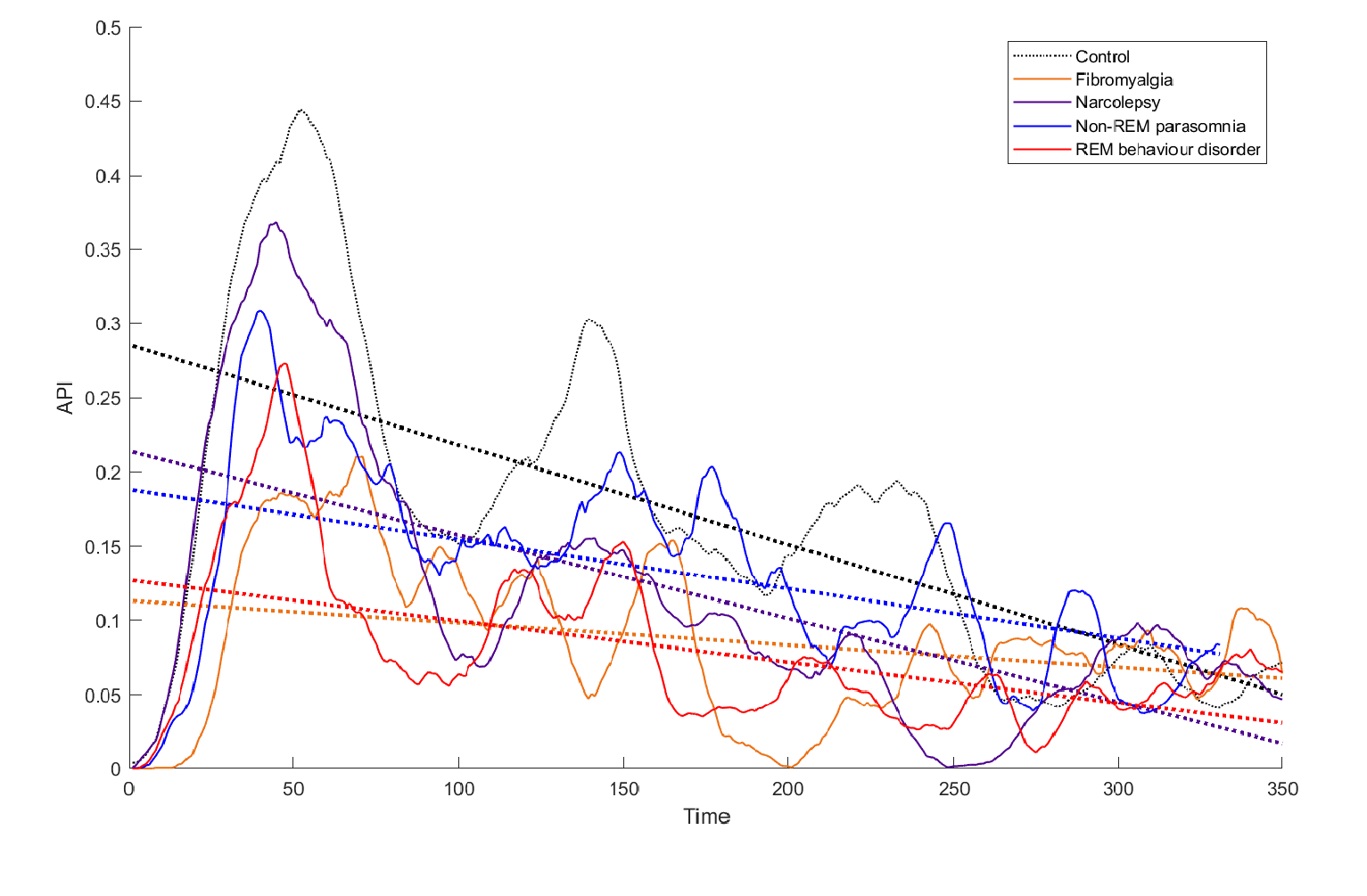
**Supplementary Figure S9.** A1 phase index profile across NREM sleep stages in all participant groups in the C3 channel. ***Abbreviations*:** **API,** A-phase Index[$\sum_{i}^{t} =0$^A(i)^/ $\sum_{i}^{t} =0$*^N(j)^*];**C3,** channels’ codes in the standard 10-20% electroencephalography montage; **NREM**, non-rapid eye movement **;REM** , rapid -eye movement sleep.


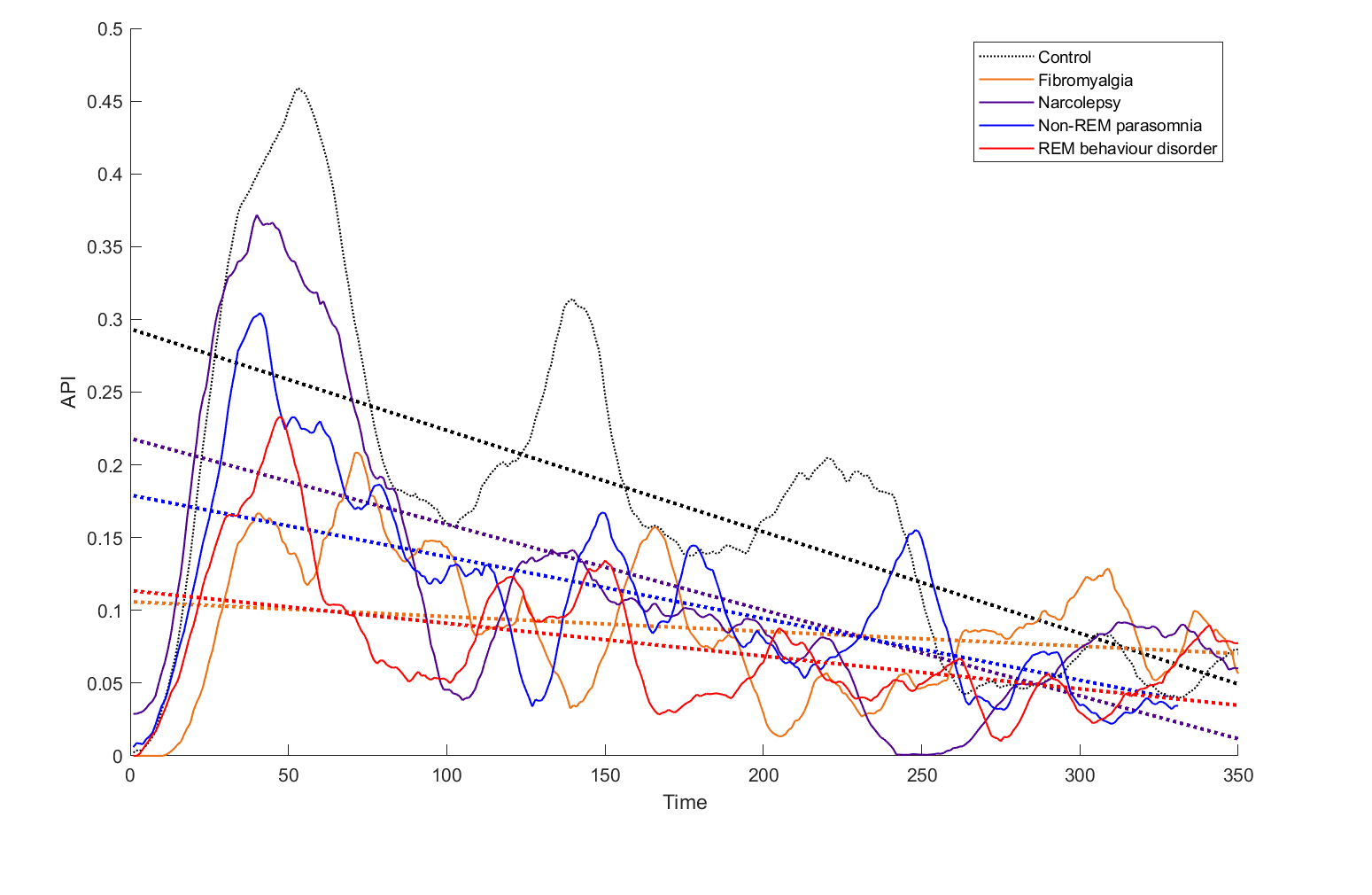


**Supplementary Figure S10.** A1 phase index profile across NREM sleep stages in all participant groups in the C4 channel. ***Abbreviations*:** **API,** A-phase Index[$\sum_{i}^{t} =0$^A(i)^/ $\sum_{i}^{t} =0$*^N(j)^*];**C4,** channels’ codes in the standard 10-20% electroencephalography montage; **NREM**, non-rapid eye movement **; iRBD** , idiopathic rapid -eye-movement sleep.


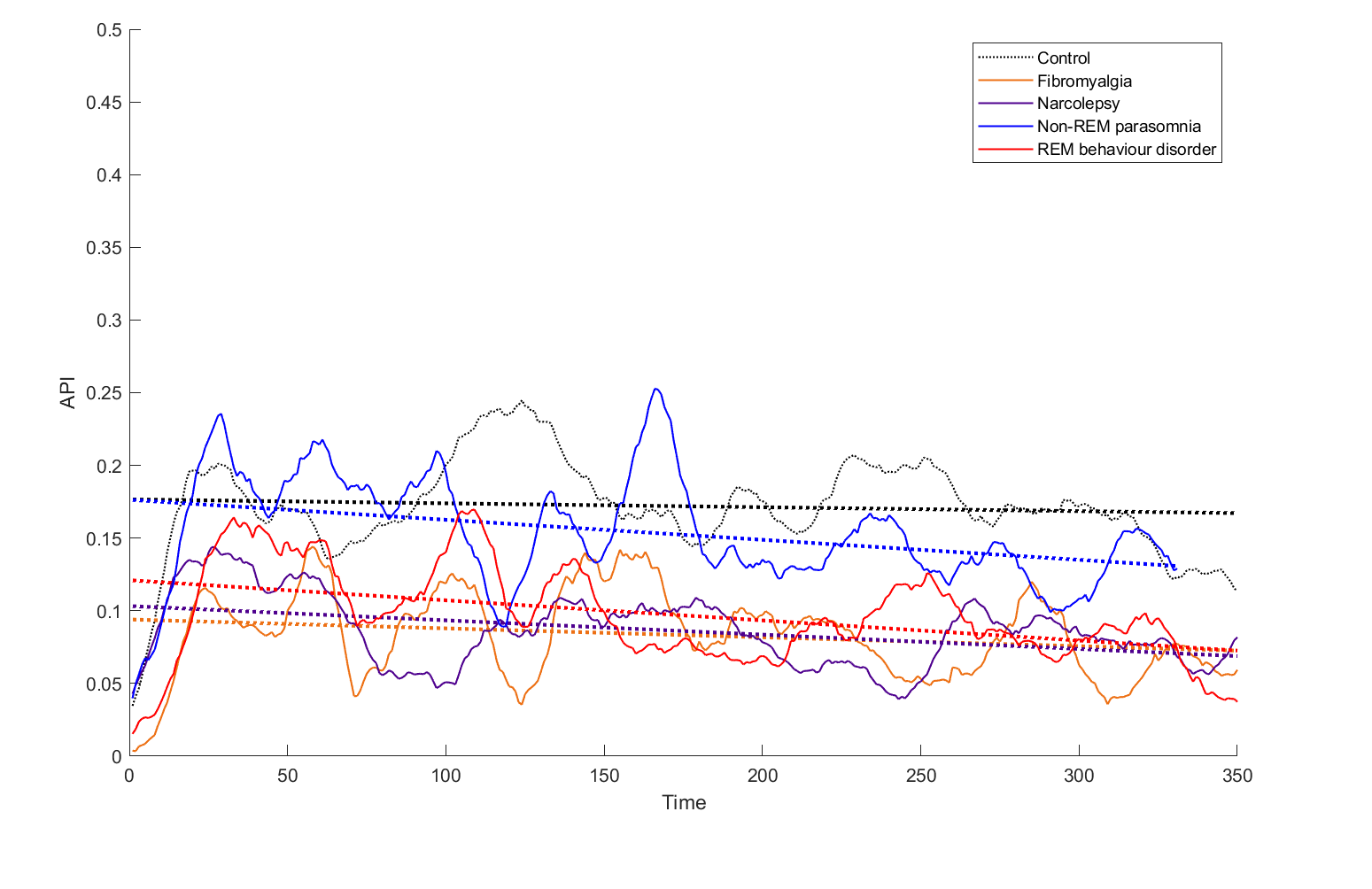


**Supplementary Figure S11.** A2 phase index profile across NREM sleep stages in all participant groups in the C3 channel. ***Abbreviations*:** **API,** A-phase Index[$\sum_{i}^{t} =0$^A(i)^/ $\sum_{i}^{t} =0$*^N(j)^*];**C3,** channels’ codes in the standard 10-20% electroencephalography montage; **NREM**, non-rapid eye movement **; iRBD** , idiopathic rapid -eye movement sleep.


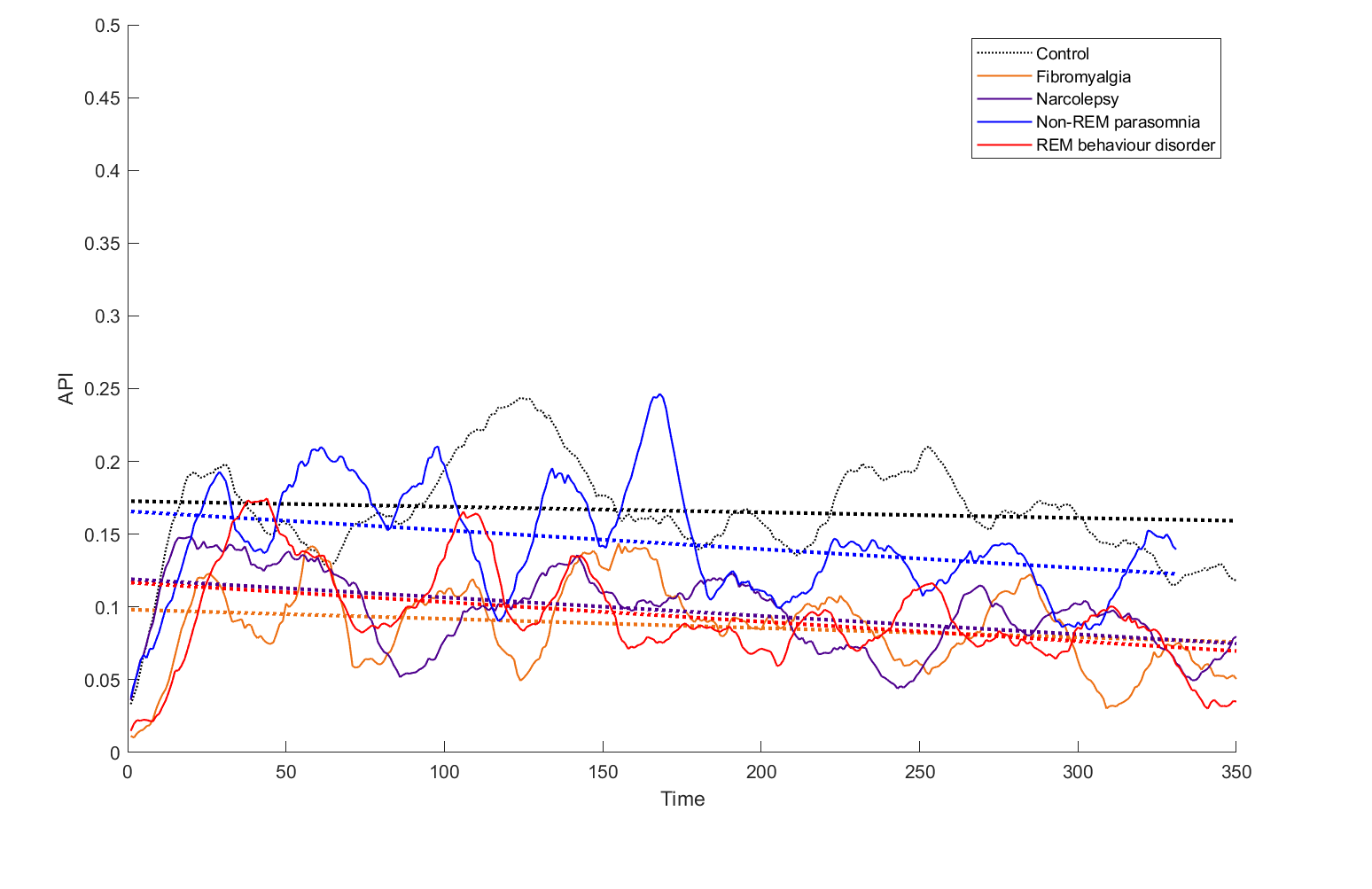


**Supplementary Figure S12.** A2 phase index profile across NREM sleep stages in all participant groups in the C4 channel. ***Abbreviations*:** **API,** A-phase Index[$\sum_{i}^{t} =0$^A(i)^/ $\sum_{i}^{t} =0$*^N(j)^*];**C4,** channels’ codes in the standard 10-20% electroencephalography montage; **NREM**, non-rapid eye movement **; iRBD** , idiopathic rapid-eye-movement sleep.


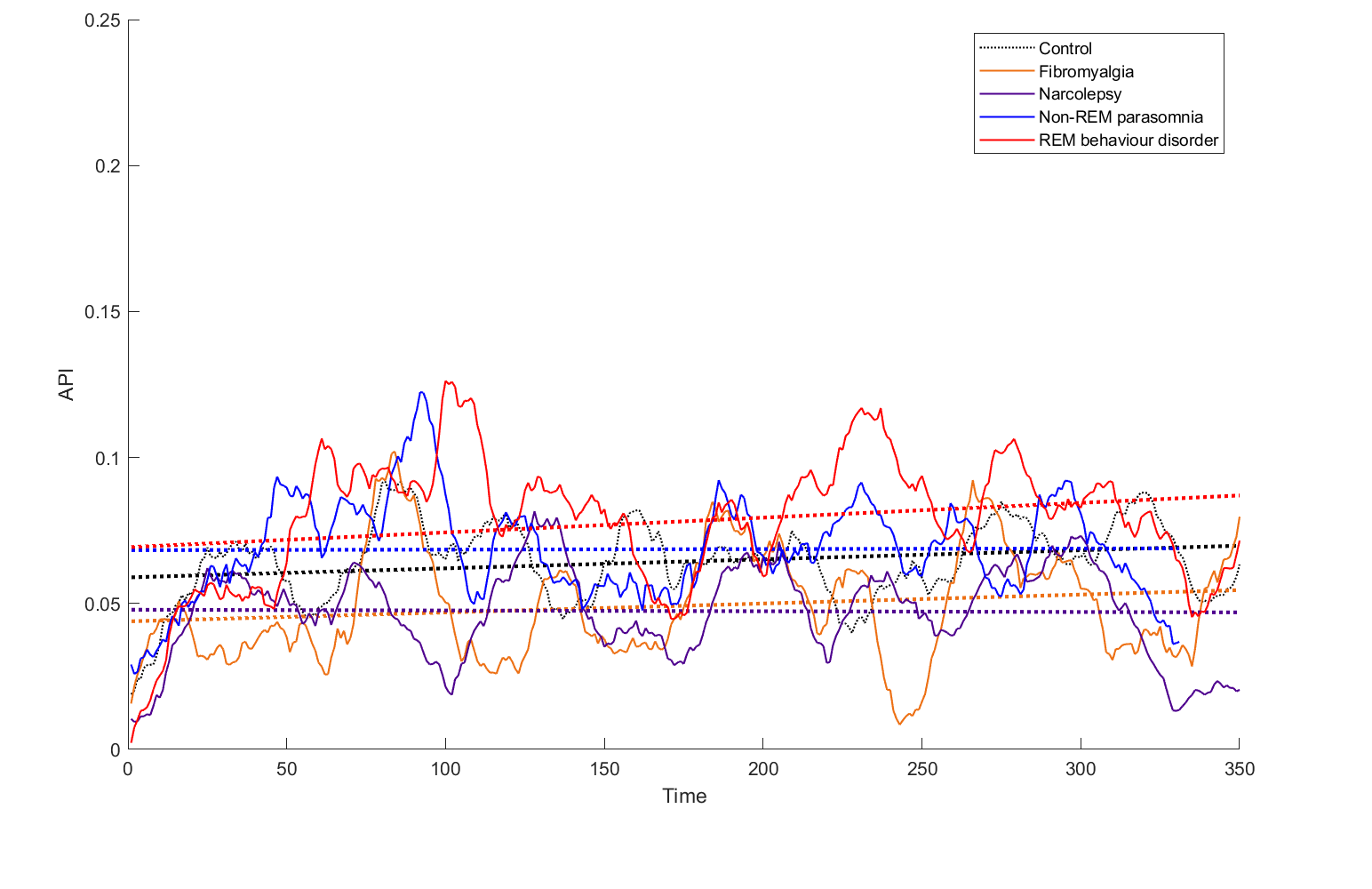


**Supplementary Figure S13.** A3 phase index profile across NREM sleep stages in all participant groups in the C3 channel. ***Abbreviations*:** **API,** A-phase Index[$\sum_{i}^{t} =0$^A(i)^/ $\sum_{i}^{t} =0$*^N(j)^*]; **C3,** channels’ codes in the standard 10-20% electroencephalography montage; **NREM**, non-rapid eye movement **; iRBD**, idiopathic rapid-eye-movement sleep.


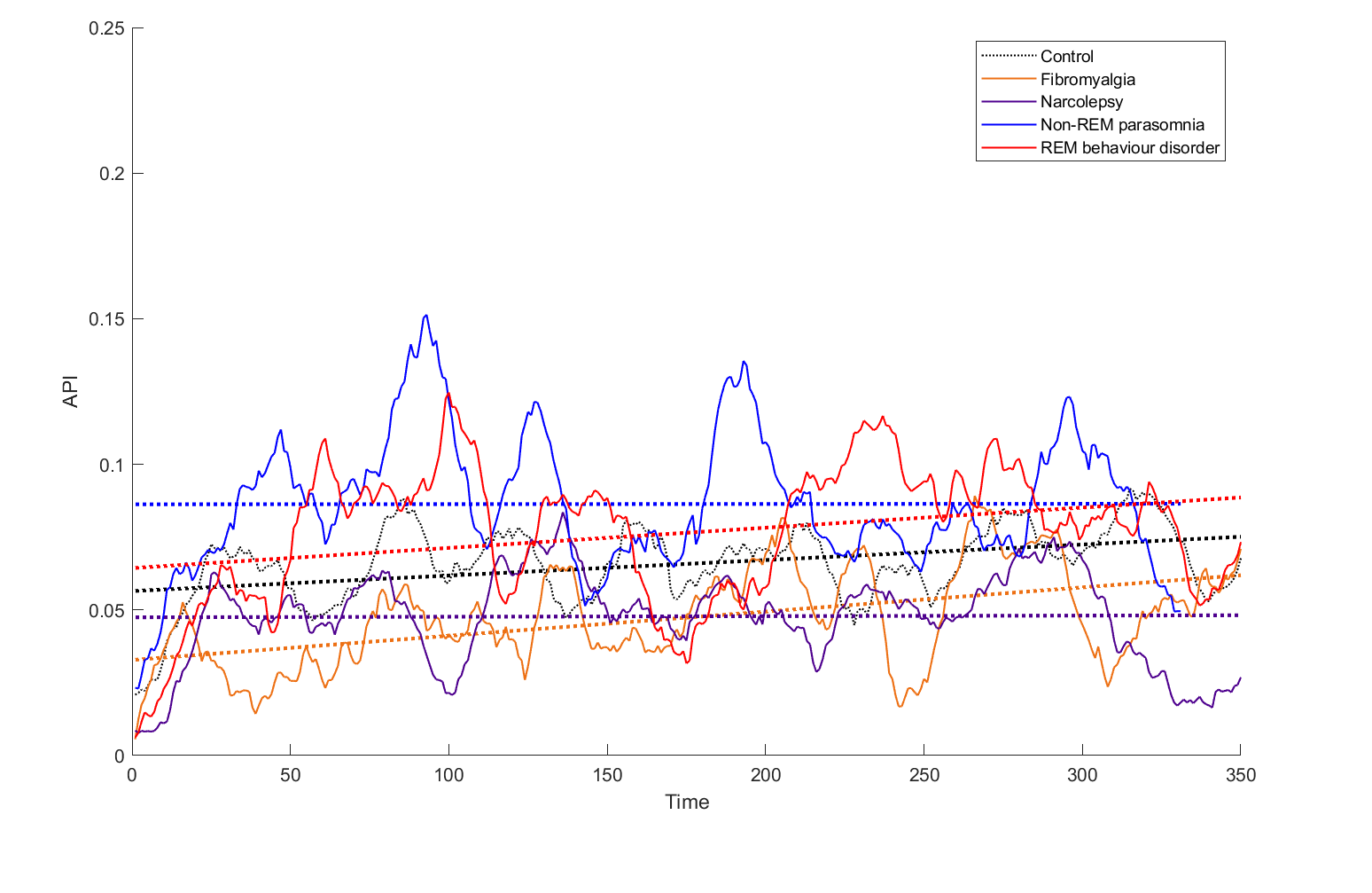


**Supplementary Figure S14.** A3 phase index profile across NREM sleep stages in all participant groups in the C4 channel. ***Abbreviations*:** **API,** A-phase Index[$\sum_{i}^{t} =0$^A(i)^/ $\sum_{i}^{t} =0$*^N(j)^*];**C4,** channels’ codes in the standard 10-20% electroencephalography montage; **NREM**, non-rapid eye movement **; iRBD**, idiopathic rapid -eye-movement sleep.

# **Supplementary Table S20. Early–late ΔAPI by group.**

ΔAPI was defined for each subject and A-phase subtype (A1–A3) as the difference between early-night and late-night NREM A-phase burden (ΔAPIₖ = APIₖ,early − APIₖ,late), where APIₖ,early and APIₖ,late are the mean APIₖ across NREM epochs with normalised time t_norm ≤ 0.25 and t_norm ≥ 0.75, respectively. At each minute, API values were first averaged across EEG channels (C3, C4). Values are reported as mean ± SD and median per group. Global group differences in ΔAPI were assessed using Kruskal–Wallis tests for each subtype: A1: H = 19.20, p = 0.0007; A2: H = 8.64, p = 0.0707; A3: H = 4.84, p = 0.3046. Pairwise Mann–Whitney U tests compare each patient group with controls; p-values are uncorrected and rank-biserial r is oriented such that positive values indicate smaller ΔAPI in the patient group than in controls.

| **Group** | **Subtype** | **n** | **ΔAPI mean ± SD** | **ΔAPI median** | **U vs Control** | **p (uncorrected)** | **rank-biserial r** |
| --- | --- | --- | --- | --- | --- | --- | --- |
| Control | A1 | 40 | 0.198 ± 0.124 | 0.199 |  |  |  |
| Fibro | A1 | 13 | 0.069 ± 0.072 | 0.086 | 98 | 0.0008 | 0.623 |
| iRBD | A1 | 26 | 0.087 ± 0.094 | 0.091 | 249 | 0.0004 | 0.521 |
| NT1 | A1 | 19 | 0.156 ± 0.124 | 0.171 | 308 | 0.2461 | 0.189 |
| NREM | A1 | 17 | 0.128 ± 0.095 | 0.125 | 220 | 0.0371 | 0.353 |
| Control | A2 | 40 | 0.073 ± 0.078 | 0.074 |  |  |  |
| Fibro | A2 | 13 | 0.041 ± 0.041 | 0.025 | 189 | 0.1450 | 0.273 |
| iRBD | A2 | 27 | 0.079 ± 0.094 | 0.050 | 541 | 0.9949 | -0.002 |
| NT1 | A2 | 19 | 0.033 ± 0.047 | 0.019 | 253 | 0.0402 | 0.334 |
| NREM | A2 | 17 | 0.081 ± 0.067 | 0.066 | 381 | 0.4799 | -0.121 |
| Control | A3 | 40 | -0.002 ± 0.042 | 0.002 |  |  |  |
| Fibro | A3 | 13 | 0.009 ± 0.025 | 0.002 | 292 | 0.5149 | -0.123 |
| iRBD | A3 | 26 | 0.012 ± 0.063 | 0.010 | 600 | 0.2968 | -0.154 |
| NT1 | A3 | 19 | 0.009 ± 0.032 | 0.009 | 444 | 0.3030 | -0.168 |
| NREM | A3 | 17 | 0.032 ± 0.047 | 0.013 | 463 | 0.0326 | -0.362 |

***References***

1 Scarpetta, S. *et al.* Criticality of neuronal avalanches in human sleep and their relationship with sleep macro- and micro-architecture. *iScience* **26**, 107840, doi:10.1016/j.isci.2023.107840 (2023).

2 Halasz, P., Terzano, M., Parrino, L. & Bodizs, R. The nature of arousal in sleep. *J Sleep Res.* **13**, 1-23 (2004).

3 Parrino, L., Ferri, R., Bruni, O. & Terzano, M. G. Cyclic alternating pattern (CAP): the marker of sleep instability. *Sleep medicine reviews* **16**, 27-45, doi:10.1016/j.smrv.2011.02.003 (2012).

4 Halász, P., Terzano, M., Parrino, L. & Bódizs, R. The nature of arousal in sleep. *Journal of sleep research* **13**, 1-23, doi:10.1111/j.1365-2869.2004.00388.x (2004).

5 Terzano, M. G. *et al.* The cyclic alternating pattern as a physiologic component of normal NREM sleep. *Sleep.* **8**, 137-145 (1985).

6 Parrino, L., Smerieri, A., Rossi, M. & Terzano, M. G. Relationship of slow and rapid EEG components of CAP to ASDA arousals in normal sleep. *Sleep.* **24**, 881-885 (2001).

7 Terzano, M. G. *et al.* Atlas, rules, and recording techniques for the scoring of cyclic alternating pattern (CAP) in human sleep. *Sleep Med.* **2**, 537-553 (2001).

8 Parrino, L. *et al.* ATLAS AND UPDATED RULES FOR THE SCORING OF CYCLIC ALTERNATING PATTERN (CAP) IN HUMAN SLEEP. A CONSENSUS REPORT BY A TASKFORCE OF THE EUROPEAN SLEEP RESEARCH SOCIETY. *Journal of sleep research* (2025).

9 Mendonça, F., Mostafa, S. S., Morgado-Dias, F., Ravelo-García, A. G. & Rosenzweig, I. Towards automatic EEG cyclic alternating pattern analysis: a systematic review. *Biomedical engineering letters* **13**, 273-291, doi:10.1007/s13534-023-00303-w (2023).

10 Troester, M. M. *et al.* *The AASM Manual for the Scoring of Sleep and Associated Events: Rules, Terminology and Technical Specifications. Version 3.*, (American Academy of Sleep Medicine, 2023).

11 Lüthi, A. & Nedergaard, M. Anything but small: Microarousals stand at the crossroad between noradrenaline signaling and key sleep functions. *Neuron* **113**, 509-523, doi:10.1016/j.neuron.2024.12.009 (2025).

12 Ferri, R. *et al.* Effects of long-term use of clonazepam on nonrapid eye movement sleep patterns in rapid eye movement sleep behavior disorder. *Sleep medicine* **14**, 399-406, doi:10.1016/j.sleep.2013.01.007 (2013).

13 Dagay, A. *et al.* Cyclic Alternating Pattern Dynamics in Individuals at Risk for Developing Parkinson's Disease. *Annals of neurology* **98**, 136-146, doi:10.1002/ana.27217 (2025).

14 Kutlu, A., Işeri, P., Selekler, M., Benbir, G. & Karadeniz, D. Cyclic alternating pattern analysis in REM sleep behavior disorder. *Sleep & breathing = Schlaf & Atmung* **17**, 209-215, doi:10.1007/s11325-012-0675-5 (2013).

15 Melpignano, A. *et al.* Isolated rapid eye movement sleep behavior disorder and cyclic alternating pattern: is sleep microstructure a predictive parameter of neurodegeneration? *Sleep* **42**, doi:10.1093/sleep/zsz142 (2019).

16 Berwick, R., Barker, C., Goebel, A. & on behalf of the guideline development, g. The diagnosis of fibromyalgia syndrome. *Clinical Medicine* **22**, 570-574, doi:<https://doi.org/10.7861/clinmed.2022-0402> (2022).

17 O'Reilly, C., Gosselin, N., Carrier, J. & Nielsen, T. Montreal Archive of Sleep Studies: an open-access resource for instrument benchmarking and exploratory research. *Journal of sleep research* **23**, 628-635, doi:10.1111/jsr.12169 (2014).

18 Sateia, M. J. International classification of sleep disorders-third edition: highlights and modifications. *Chest* **146**, 1387-1394, doi:10.1378/chest.14-0970 (2014).

19 Treede, R. D. *et al.* Chronic pain as a symptom or a disease: the IASP Classification of Chronic Pain for the International Classification of Diseases (ICD-11). *Pain* **160**, 19-27, doi:10.1097/j.pain.0000000000001384 (2019).

20 World Medical, A. World Medical Association Declaration of Helsinki: ethical principles for medical research involving human subjects. *JAMA* **310**, 2191-2194, doi:10.1001/jama.2013.281053 (2013).

21 Council, E. P. a. o. t. Vol. Regulation (EU) 2016/679 (ed EU) (Official Journal of the European Union, 2016).

22 Parrino, L. & Vaudano, A. E. The resilient brain and the guardians of sleep: New perspectives on old assumptions. *Sleep medicine reviews* **39**, 98-107, doi:10.1016/j.smrv.2017.08.003 (2018).

23 Poryazova, R., Werth, E., Parrino, L., Terzano, M. G. & Bassetti, C. L. Cyclic alternating pattern in narcolepsy patients and healthy controls after partial and total sleep deprivation. *Clinical neurophysiology : official journal of the International Federation of Clinical Neurophysiology* **122**, 1788-1793, doi:10.1016/j.clinph.2011.02.028 (2011).

24 Terzano, M. G. *et al.* Cyclic alternating pattern (CAP) alterations in narcolepsy. *Sleep medicine* **7**, 619-626, doi:10.1016/j.sleep.2005.12.003 (2006).

25 Guilleminault, C., Kirisoglu, C., da Rosa, A. C., Lopes, C. & Chan, A. Sleepwalking, a disorder of NREM sleep instability. *Sleep medicine* **7**, 163-170, doi:10.1016/j.sleep.2005.12.006 (2006).

26 Rizzi, M. *et al.* Cyclic alternating pattern: a new marker of sleep alteration in patients with fibromyalgia? *The Journal of rheumatology* **31**, 1193-1199 (2004).

27 Rosenblum, Y. *et al.* Fractal cycles of sleep, a new aperiodic activity-based definition of sleep cycles. *eLife* **13**, doi:10.7554/eLife.96784 (2025).

28 Halász, P., Timofeev, I. & Szűcs, A. Derailment of Sleep Homeostatic Plasticity Affects the Most Plastic Brain Systems and Carries the Risk of Epilepsy. *Journal of integrative neuroscience* **22**, 111, doi:10.31083/j.jin2205111 (2023).
